# Supplementary material for: EpiGePT: a pretrained transformer-based language model for context-specific human epigenomics
Source: Genome Biol. 2024 Dec 18;25:310. doi: 10.1186/s13059-024-03449-7 (PMC11657395; doi:10.1186/s13059-024-03449-7)
Supplement: Supplementary file 1 — Additional file 1: Text S1: Data splitting strategy for model training. Text S2: Data processing for ChromHMM annotation data. Text S3: Details of the comparison matrix in functional chromatin status prediction experiments. Text S4: Implementation of Enformer model and Enformer+. Text S5: Description and comparison of EpiGePT with different settings. Text S6: Guidance on how to use EpiGePT in two scenarios without tissue-specific TFs data. Text S7: System design and implementation of the web server. Text S8: Case application of the EpiGePT-online. Text S9: Conditions and data preparation for retraining the EpiGePT model on other species. Text S10: Running time of the EpiGePT and baseline methods. Text S11: Computational resources used to train the model. Fig S1: Model architecture of EpiGePT for multiple epigenomic signals prediction. Fig. S2: Three data partitioning strategies for model training and testing. Fig. S3: EpiGePT's performance in predicting DNase-seq and other epigenetic signals. Fig. S4: Comparison of cross-cell-type chromatin accessibility (DNase-seq profiles) prediction performance between EpiGePT and baseline methods. Fig. S5: Performance of EpiGePT and baseline methods on chromatin states classification and causal variants classification. Fig. S6: The average predictive performance of the remaining methods when each model serves as the ground truth. Fig. S7: The performance comparison of retrained Enformer with different numbers of encoder layers for predicting chromatin accessibility. Fig. S8: Performance of EpiGePT in cross-cell-type prediction. Fig. S9: Zero-shot learning of EpiGePT on chromatin accessibility (DNase-seq) prediction on mouse data. Fig. S10: Model fine-tuning EpiGePT on chromatin accessibility (DNase-seq) prediction on mouse data. Fig. S11: The cross-chromosome predictive performance of pretrained EpiGePT and EpiGePT-seq after fine-tuning on mouse data. Fig. S12: The performance (auROC) of attention score of EpiGePT in distinguish [file 13059_2024_3449_MOESM1_ESM.pdf]

# Supplementary Information

# EpiGePT: a pretrained transformer-based language model for context-specific human epigenomics

Zijing Gao[1,#], Qiao Liu[2,#,*], Wanwen Zeng[2], Rui Jiang[1,*] and Wing Hung Wong[2,3,*]

[1] Ministry of Education Key Laboratory of Bioinformatics, Bioinformatics Division at the Beijing National Research Center for Information Science and Technology, Center for Synthetic and Systems Biology, Department of Automation, Tsinghua University, Beijing 100084, China;

[2] Department of Statistics, Stanford University, Stanford, CA 94305, USA;

[3] Department of Biomedical Data Science, Bio-X Program, Center for Personal Dynamic Regulomes, Stanford University, Stanford, CA 94305, USA;

* To whom correspondence should be addressed.

[#] The first two authors contributed equally.

E-mail:

Zijing Gao: gzj21@mails.tsinghua.edu.cn

Qiao Liu: liuqiao@stanford.edu

Wanwen Zeng: wanwen@stanford.edu

Rui Jiang: ruijiang@tsinghua.edu.cn

Wing Hung Wong: whwong@stanford.edu

# Contents


Supplementary Texts ........ 4

Text S1. Data splitting strategy for model training. ........ 4

Text S2. Data processing for ChromHMM annotation data. ........ 5

Text S3. Details of the comparison matrix in functional chromatin status prediction experiments. ........ 6

Text S4. Implementation of Enformer model and Enformer+. ........ 8

Text S5. Description and comparison of EpiGePT with different settings. ........ 10

Text S6. Guidance on how to use EpiGePT in two scenarios without tissue-specific TFs data. ........ 12

Text S7. System design and implementation of the web server. ........ 14

Text S8. Case application of the EpiGePT-online. ........ 15

Text S9. Conditions and data preparation for retraining the EpiGePT model on other species. ........ 16

Text S10. Running time of the EpiGePT and baseline methods. ........ 19

Text S11. Computational resources used to train the model. ........ 20

Supplementary Figures ........ 21

Fig. S1 ........ 21

Fig. S2 ........ 23

Fig. S3 ........ 24

Fig. S4 ........ 26

Fig. S5 ........ 27

Fig. S6 ........ 28

Fig. S7 ........ 29

Fig. S8 ........ 30

Fig. S9 ........ 31

Fig. S10 ........ 32

Fig. S11 ........ 33

Fig. S12 ........ 35

Fig. S13 ........ 36

Fig. S15 .... 38
Fig. S16 .... 40
Fig. S17 .... 41
Fig. S18 .... 42
Fig. S19 .... 43
Fig. S20 .... 44
Fig. S21 .... 45
Fig. S22 .... 46
Fig. S23 .... 47
Fig. S24 .... 48
Fig. S25 .... 49
Fig. S26 .... 50
Fig. S27 .... 51
Fig. S28 .... 52
Fig. S29 .... 53
Fig. S30 .... 54
Fig. S31 .... 55
Fig. S32 .... 56
Fig. S33 .... 57
References .... 58

# Supplementary Texts

## Text S1. Data splitting strategy for model training.

To comprehensively validate the performance of EpiGePT in predicting chromatin accessibility, we adopted three different data splitting strategies in the DNase-seq profiles (1) prediction experiment to verify the model's prediction ability when facing new genomic regions and cell types, which can meet researchers' usage needs to the maximum extent. Firstly, cross-cell type prediction refers to splitting the training and testing sets according to cell types in the same genomic region, where the cell types in the testing set have not appeared in the training set (Figs. S1b). Secondly, cross-genomic region prediction refers to splitting the training and testing sets according to genomic regions in the same cell type (Figs. S1a). Thirdly, simultaneous cross-cell type and genomic region prediction, where the prediction can be performed in completely novel cell types and genomic regions with the expression of transcription factors in that cell type. The training set needs to subset both cell types and genomic regions (Figs. S1c). To complete the latter two auxiliary predictions, we also split the data into 5 folds according to both cell types and genomic regions, so that both cross-validation can be performed in one round of training, but this will also reduce the amount of training and testing data.

## Text S2. Data processing for ChromHMM annotation data.

We downloaded the 15-state ChromHMM (2) annotations across 127 epigenomes from the ROADMAP project. The state of chromatin is annotated for each 200bp bin in a specific cell type. RNA-seq data of TFs across 56 cell types were download and extracted from the ROADMAP (3) project (Supplementary table S10 and S11). Subsequently, we mapped the 711 transcription factors to the downloaded RNA-seq data, resulting in the identification of RNA-seq data for 642 transcription factors. In the subsequent experiments, we utilized the expression data of these 642 transcription factors. We finally calculated the normalized TPM values of the 642 TFs on 56 cell types we extracted for the using in the classification model. For coarse grain chromatin state prediction, we took the state 'Quies' as low signal regions and other states as signal regions. For fine grain chromatin state prediction, we extracted the state 'TssA', 'TssAFlnk', 'TssBiv' and 'BivFlnk' as TSS regions, state 'EnhG', 'Enh' and 'EnhBiv' as enhancer regions, 'Quies' as low signal regions and other state as other regions. To balance the number of different chromatin states, we downsampled the low signal regions and obtained 921,074 bin each cell line finally.

## Text S3. Details of the comparison matrix in functional chromatin status prediction experiments.

Regarding the experimental setup for the additional benchmarking. Based on the unsupervised nature of ChromHMM, we used ChromHMM's annotations as the ground truth to train EpiGePT, ChromDragoNN, and DeepCAGE. We then converted their predicted results (probabilities) into categorical labels and used these labels as ground truth to evaluate the performance of other methods. For ChromHMM, we downloaded its posterior probabilities from https://egg2.wustl.edu/roadmap/web_portal/chr_state_learning.html to obtain its predicted categories. Consequently, we computed the results for the four methods as ground truth through five-fold cross-validation across 56 cell types. For metric selection, we used accuracy, auROC (area under the Receiver Operating Characteristic Curve), auPRC (Area Under the Precision-Recall Curve), and F1 score.

Next, we conducted comprehensive experiments here by treating the predicted results from one method as ground truth and evaluating the predicted results of other methods. As shown in Fig. S28, we plotted four heatmap matrices, each denoting the results of using different methods as ground truth across the four metrics. Specifically, each row of the matrix represents the performance of the remaining methods using a given method as the ground truth, with the values in each position reflecting the average metric across the 56 cell types in five-fold cross-validation. The diagonal elements of the matrix are 1, indicating the performance metrics computed with itself as the ground truth. From the figure, it can be observed that EpiGePT consistently outperforms the other three methods across most metrics when using DeepCAGE and ChromDragoNN as ground truth. When using ChromHMM as the ground truth, EpiGePT achieves the highest score across all metrics. The results demonstrate that EpiGePT has a stronger ability to learn from the chromatin state information contained in the ground truth labels and performs better in predictions when ground truth is available. Additionally, we found that EpiGePT continues to achieve best performance when using DeepCAGE as the ground truth. However, when using ChromDragoNN as the ground truth,

EpiGePT is only slightly worse than DeepCAGE but better than other methods. These results suggest that the predicted labels of EpiGePT are less similar to those of ChromDragoNN compared to DeepCAGE. In other words, compared to ChromDragoNN and DeepCAGE, EpiGePT can predict many common functional chromatin regions, while also correctly identifying regions that the other two methods misclassified. This comprehensive evaluation of the predictive performance across models not only illustrates the differences between them but also emphasizes the superior accuracy and robustness of EpiGePT across various metrics and cell types.

## Text S4. Implementation of Enformer model and Enformer+.

To ensure a fair comparison between models and prevent the possibility of information leakage, we implemented the Enformer (4) model ourselves and trained it on our own collected data. Due to differences in dataset size and partitioning compared to Enformer, we reduced the number of encoder layers in Enformer to prevent overfitting and reduce computational resource requirements during retraining, without compromising performance. We also retrained the Enformer with 3, 6, 9, and 12 encoder layers under the same 103 cellular contexts and validated the model's prediction performance on 26 test cell types, observing no significant changes in the average PCC and SCC. In fact, the 3-layer model exhibited slightly better performance in terms of median scores, although the highest improvement in PCC was only about 0.7% (Fig. S31). Specifically, the average PCC for the 3-layer model was 0.724, slightly lower than the 9-layer model at 0.726, but higher than the 12-layer model at 0.718. Thus, we reduced the number of encoder layers in Enformer to 3. Additionally, we introduced Enformer+ to enable a fair comparison between EpiGePT and Enformer in bin-level prediction. As Enformer takes only the DNA sequence as input, it tends to predict the same values for the same locus in different cell types, resulting in a loss of locus-level prediction ability. To address this, we incorporated the binding status and expression of the same transcription factors in Enformer+, and compared it to EpiGePT's performance on the same tasks. The purpose of designing Enformer+ is to enable locus-level prediction capability. We compared EpiGePT and retrained Enformer to demonstrate that the model architecture of EpiGePT offers stronger predictive accuracy than Enformer when trained on the same dataset (Fig.S3c, Fig. S31). Specifically, 1) the comparison between EpiGePT and the retrained Enformer shows that using Enformer's pure DNA sequence architecture makes it difficult to accurately predict epigenomic signals in unseen cell types. This is because the DNA sequences are identical across different cell types, so even when trained in various cellular contexts, the retrained Enformer cannot distinguish the same region under different contexts and can only predict an averaged epigenomic signal. 2) the comparison between EpiGePT and the

retrained Enformer+ shows that the convolution tower and other operations applied to the DNA sequence in Enformer did not improve predictive accuracy. **3)** By comparing the retrained Enformer+ with the retrained Enformer, we can also confirm the improvement in predictive accuracy brought by the incorporation of reference TF binding profiles.

## Text S5. Description and comparison of EpiGePT with different settings.

We have organized the models with different settings mentioned by the reviewer into a table, categorizing them into Epigenomic Profiles Prediction and Chromatin Interactions Prediction based on their respective tasks. As shown in Table S19, our models and different settings are divided into two parts: EpiGePT and EpiGePT-seq are models for predicting epigenomic profiles, while the other settings, A-C, are designed to explore the model's ability to capture chromatin interactions.

For epigenomic profiles prediction, we need to firstly emphasize that for future usage, we provide users with the pretrained EpiGePT as the base model. The main purpose of designing EpiGePT-seq is to perform an ablation study to validate the improvement brought by incorporating TF profiles into the model, rather than to offer it as a pretrained model for users. Therefore, we have sufficiently compared the performance of EpiGePT with EpiGePT-seq, which uses only DNA sequence as input. As shown in Fig. 2a and Fig. S3b, EpiGePT consistently achieved better results across three tasks related to predicting epigenomic signals, such as chromatin accessibility measured by DNase-seq. Additionally, EpiGePT outperformed EpiGePT-seq under various numbers of training cellular contexts with cross-cell-type predictions on DNase-seq (Fig. S26-S27). Furthermore, we fine-tuned the models on data from three mouse cell types using EpiGePT and EpiGePT-seq, which were pretrained on 100 cellular contexts. The results showed that in brain, kidney, and lung cellular contexts, EpiGePT achieved superior performance even with limited mouse data (Fig. S17). These findings demonstrate that EpiGePT, with the integration of the TF module, not only improves performance on epigenomic profiles prediction tasks but also brings the model with the capability for predicting in unseen cell types. This is the primary distinction between EpiGePT and current methods based solely on DNA sequences.

Additionally, we provided a performance comparison for cross-cell-type prediction on DNase-seq profiles (hg19 reference genome) (Fig. S31), which includes EpiGePT, EpiGePT-seq,

BIRD, ChromDragoNN, Enformer, and Enformer+. It is evident that EpiGePT achieves the best performance, and this figure clearly demonstrates the improvement that EpiGePT brings over baseline methods, particularly in our key task of cross-cell-type prediction. In summary, the performance comparison for the epigenomic profiles prediction task was conducted among EpiGePT, EpiGePT-seq, and baseline methods.

For chromatin interaction prediction, we presented three settings based on the same model architecture with identical forward propagation processes and inputs. The difference is in the approaches: settings A and B utilize self-attention scores to prioritize chromatin interactions, while setting C replaces the output components with a new MLP classifier to predict interaction relationships between anchors.

Overall, the three settings are intended for two different application scenarios. First, in situations where real label data is lacking, such as cell types without 3D genome data, settings A and B can be used. For these two models, we recommend using EpiGePT-3D (setting B) for chromatin interaction prediction. In the manuscript, we have demonstrated through the comparison of setting A and setting B that, when training the model on the same data, EpiGePT-3D exhibits more biologically meaningful self-attention scores than EpiGePT, allowing it to better prioritize real chromatin interactions (Fig. 3d-e). Second, in scenarios where real label data is available but there is a need to predict additional chromatin interaction pairs, the finetuned model is a more accurate choice. However, the output of the finetuned EpiGePT is not epigenomic profiles, nor is it designed to predict epigenomic signals; therefore, it is unnecessary to compare it with EpiGePT in the epigenomic profiles prediction task. In the manuscript, we compared finetuned EpiGePT with baseline methods (DeepTACT, Kmer-MLP) and demonstrated that finetuned EpiGePT outperforms these baselines. Additionally, using data lifted to hg38, we showed that EpiGePT-3D provides performance improvements over both EpiGePT and baseline methods (Fig. S29-S30, with weight $\alpha$ for 3d genome loss chosen as 1). Overall, in both application scenarios and under the same training conditions, EpiGePT-3D consistently outperforms EpiGePT, making it the recommended choice for chromatin interaction prediction.

## Text S6. Guidance on how to use EpiGePT in two scenarios without tissue-specific TFs data.

**Scenario 1** involves users, especially those with biological background, who have a cell line/tissue/celluar context of interest and have already conducted gene expression experiment (e.g., RNA-seq) of this context. Note that the expense of RNA-seq is much lower than those epigenomic sequencing experiments. If they want to use EpiGePT to make predictions of epigenomic profiles for that specific cell type and infer the gene regulatory mechanism. In this case, users simply need to follow the tutorial provided on our website (http://health.tsinghua.edu.cn/epigept/tutorial.php) and prepare the model input data for inference. Our website provides the online inference if users uploaded their gene expression data and the genomic regions of interests. Since users have conducted their own sequencing experiments, there will be no issue of lacking TF expression data.

**Scenario 2** involves users who have not conducted gene expression sequencing experiments but want to explore the epigenetic signals of a particular cell line or tissue using public data. To facilitate this issue, we have tried our best to provide a comprehensive TF reference dataset, which covers the major cell types that are publicly available. In most cases, users can easily find the corresponding expression data. For example, in the ENCODE project (5) has aggregated a vast amount of bulk sequencing data, we compiled data that includes polyA plus RNA-seq and total RNA-seq experiments. We analyzed the distribution of experiments across tissues, cell lines, primary cells, and in vitro differentiated cells, and presented the results in a pie chart (Fig. S20). In the human reference genome GRCh37, there are a total of 575 different experimental datasets covering 218 cell lines or tissues. In the reference genome GRCh38, there are 1,015 different experimental datasets that encompass 281 unique cell lines and tissues. Existing research indicates that there are more than 400 cell types in the human body (6). Therefore, we believe that the bulk sequencing data available on ENCODE project has already covered most cell types in human body.

In addition, with the rapid advancement of single-cell sequencing technology, the scale of single-cell RNA sequencing (scRNA-seq) data has grown rapidly. Expression data from different cell types covered by single-cell data, when processed into pseudo-bulk data, can also be used as model input. For instance, Cellxgene database (7) has already collected scRNA-seq data from over 91.7 million human cells. Our statistical analysis of the data, as of September, 2024, shows that human data covers 280 tissues and 721 cell types (Fig. S21 and Fig. S22). Even in the mouse data, the cell count exceeds 42 million, covering 98 tissues and 424 cell types. Therefore, in actual usage scenarios, gene expression data (TPM value) for most cell types can be found. If a perfectly matching dataset is not available, users can also select a similar cell type from reference data based on the cell type description to serve as input for the TF module and utilize the model accordingly.

## Text S7. System design and implementation of the web server.

EpiGePT-online runs on a Linux-based Apache web server (https://www.apache.org) and utilizes the Bootstrap v3.3.7 framework (https://getbootstrap.com/docs/3.3/) for its web-frontend display. The backend of the server uses PHP v7.4.5 (http://www.php.net). The platform is compatible with the majority of mainstream web browsers, including Google Chrome, Firefox, Microsoft Edge, and Apple Safari.

## Text S8. Case application of the EpiGePT-online.

The online prediction web service of EpiGePT enables users to predict eight types of epigenomic signals using EpiGePT without the need for setting up environments, writing code, or computational resources. In this section, we describe a usage scenario of EpiGePT-online for epigenomic signals prediction (Fig. S13). Users are provided with the flexibility to annotate either multiple genomic regions or a single locus at their discretion. Assuming an algorithmic researcher is interested in determining the potential regulatory role of a specific chromatin region based on its epigenetic modifications. In this case, the researcher can utilize EpiGePT-online to calculate the epigenetic signals on this region, to obtain references for assessing the potential regulatory role of the region. The submission prerequisites encompass two essential components. 1) The expression profiles of 711 TFs, which facilitate EpiGePT in acquiring precise cell type or tissue information. 2) The specific location of a locus on the genome or uploading of a bed file containing the information of genomic regions. It is worth noting that each line in the uploaded BED file should correspond to a 128kbp region to comply with the input length requirement of EpiGePT. If users select a specific locus, we will provide the predicted results for the region spanning 128kbp upstream and downstream of that locus. The web server allows users to upload expression values of 711 TFs in either numpy or comma-separated values (CSV) format. When predicting for $N$ genomic regions, users can obtain a downloadable matrix stored in CSV format with dimensions $(N \times 1000, 8)$. Each row denotes a 128bp genomic bin, and each column denotes an epigenetic profile. The specific referents of each row and column are provided in the downloadable table. This allows users to perform downstream analyses, such as related analyses in the areas of gene regulation and human disease.

## Text S9. Conditions and data preparation for retraining the EpiGePT model on other species.

For the necessary conditions, the data on new species must meet: (i) a high-quality and complete reference genome and (ii) a high degree of conservation in transcription factors (TFs) compared to humans. These two conditions are prerequisites for fine-tuning the model. This is because EpiGePT's core modeling is focused on learning the relationship between key human TFs and their genomic region binding. If there is low functional and structural conservation of TFs across species, the model cannot effectively learn from this. Therefore, cross-species transfer is only possible when these two conditions are met. For example, organisms such as mouse and chimpanzee are more applicable for cross-species model transfer.

For data preparation, researchers need to prepare data from three aspects: experimental epigenomic data, DNA module input, and TF module input.

**1) Experimental epigenomic data preparation**. In the corresponding species, researchers need to collect bulk sequencing data from the relevant cell line or tissue. Using DNase-seq as an example, researchers need to first collect the BAM sequencing files and use samtools and bedtools to calculate the read count of the sequencing files over the specified genomic regions (BED file) (refer to the tutorial provided in our GitHub: https://github.com/ZjGaothu/EpiGePT). As for the selection of regions, researchers can either split the entire genome and filter for regions that overlap with epigenomic signals from multiple cell types, similar to our approach, or select specific regions based on their research interests. It is important to note that the length and resolution of the regions need to be consistent with the pretrained human model. For example, the DNase-seq prediction model mentioned earlier uses 10kbp regions with 200bp resolution, while pretrained model for predicting multiple epigenomic profiles uses 128kbp regions with 128bp resolution. Finally, by applying a logarithmic transformation to the read counts, the corresponding ground truth data can be obtained. The ground truth for the *i*-

th epigenomic track in the *j*-th region can be obtained using the following mathematical expression:

$$Epi\ profile_{i,j} = \log\left(readcount_{i,j} + 1\right)$$

**2) DNA module input preparation**. For the DNA module input, users only need to collect the reference genome fasta file for the corresponding species. For instance, in the case of mouse, the GRCm38 (mm10) version can be used. It is important to note that the fasta file must match the reference genome used in the sequencing experiments (Epigenomic files and RNA-seq files). During model training, the DNA sequence can be read from the reference genome using Pyfasta and then converted into an $L \times 4$ one-hot matrix.

**3) TF module input preparation**. For the TF module, the input includes expression data for TFs and their binding status to the genomic regions. For the expression data, researchers need to collect RNA-seq data corresponding to the same cell line or tissue associated with the ground truth epigenomic data. If fine-tuning is performed on a specific cell line or tissue, the TPM (Transcripts Per Million) value can be directly utilized and quantile normalized (using the reference TF expression file we provided) to a 711-dimensional TF expression vector. Detailed tutorials on processing these data are available on our GitHub and website (https://github.com/ZjGaothu/EpiGePT, http://health.tsinghua.edu.cn/epigept/tutorial.php). It is important to note that other species may not have a one-to-one correspondence with these 711 human TFs. For missing TF expressions, we can set them to zero or we can replace them with the mean expression of the corresponding TF from the human reference expression profile. For the TF binding status, the DNA binding specificity of TFs is generally highly conserved between related organisms. In mammalian (or even vertebrate) organisms, the human motif files can be used for substitution, utilizing the Homer tool (8) to scan the TF binding status in the corresponding regions. However, for different sets of organisms, such as fruit flies, yeast, or plants, which often contain entirely different transcription factors, it is necessary to locate the corresponding motif database and then use the Homer tool for scanning (8). Then, the binding status features for the 711 corresponding TFs can be obtained.

By performing an element-wise product of these two features, the input for the TF module during model training can be generated.

If the conditions are met and the three types of data can be obtained, users can proceed to transfer the model to a new species for fine-tuning.

In species like plants or fungi, where gene expression and regulatory patterns can differ significantly, the primary challenge lies in identifying a set of TFs to model the binding status of TFs to genomic regions across different cell types. Specifically, we collected gene annotation files for widely studied plants from the Ensembl Plants project (9). Taking *Arabidopsis thaliana* and *Oryza sativa*, two commonly studied species, as examples, we identified only 26 and 9 corresponding TFs out of the 711 TFs. Overall, among the gene annotation files of the 83 plant species provided by the Ensembl Plants project (9), we found overlap with our selected 711 TFs in only four species, with arabidopsis thaliana exhibiting the highest overlapping proportion of only 3.66% (Fig. S18a-b). The small shared TF gene regulation mechanism indicates that transferring human model to plant data is challenging. Fungi exhibit even lower similarity to human TFs in terms of both sequence and function. For example, from FungiDB (10, 11), we retrieved the gene annotation file for *Aspergillus aculeatus ATCC 16872*, which showed 0% overlap with the 711 human TFs, making it unable to transfer the pre-trained human model to this species. In practice, the degree of overlap in TFs can be used as one criterion for determining the feasibility of model transfer. For organisms such as plants and fungi, where the overlap in TFs is less than 5%, fine-tuning the model is highly challenging. In contrast, vertebrate organisms such as mouse exhibit a high degree of conservation in their overall gene expression profiles compared to humans (12), which provides a strong foundation for transferring EpiGePT to these species.

## Text S10. Running time of the EpiGePT and baseline methods.

To demonstrate the computational efficiency of our model, we recorded the runtime of EpiGePT and baseline methods for one epoch on two sets of experiments, with different data sizes and input sequence lengths. Firstly, in the DNase signal prediction experiment on 129 cell types, with an input sequence length of 10kbp and using the same training data, Enformer requires approximately 3 hours and 4 minutes to complete one epoch, while EpiGePT only takes 2 hours and 17 minutes. In contrast, ChromDragoNN (13), which uses a genomic bin rather than a long region as the model input, requires 24 hours for pre-training and 8 hours for fine-tuning. In this case, the batch size of ChromDragoNN was set to 1024, which is equivalent to EpiGePT using a batch size of around 20. This modeling and computation approach presents challenges in terms of computational efficiency when dealing with large amounts of data. DeepCAGE (14) faces similar efficiency issues using the same approach. Even with a batch size of 256 on a single GPU, it still takes nearly 10 hours to complete one epoch of training. Secondly, we also recorded the running time of the models under larger-scale data and longer input sequences. When the number of input genomic bins increased from 50 to 1000, which corresponds to an input sequence length of approximately 128k, EpiGePT took approximately 3 hours to complete one epoch of training on 20 cell lines and 13,300 genomic regions, while Enformer required approximately 27 hours to train one epoch, as it required a longer input sequence of approximately 190kbp. Furthermore, EpiGePT without TF module (EpiGePT-seq) had approximately 1/4 of the parameters of Enformer and took approximately 2 hours and 40 minutes to train. In terms of performance, EpiGePT-seq performed similarly to Enformer on this dataset. This also explains why we chose to simplify the pure sequence model rather than directly adding a TF module to Enformer.

## Text S11. Computational resources used to train the model.

The total parameter size of EpiGePT is 71.3M, making it feasible to train and make inference on a single GPU. We also tracked the performance across three hardware configurations (NVIDIA GeForce RTX 3090, RTX 4090, and NVIDIA RTX A6000), each using a single GPU (Table S12-S15, Fig. S19). For example, on a single NVIDIA RTX A6000 (48GB memory), peak GPU memory usage reached 31.8GB with a batch size of 16, and training one epoch took around 14 hours. The training dataset comprised 85 cell lines or tissues and 15,870 regions of 128kbp, totaling roughly 15 million bins. Training the full model on a single A6000 GPU would take approximately 2–3 weeks. However, this process can be significantly accelerated by utilizing multiple GPUs in parallel, employing either Data Parallel (DP) or Distributed Data Parallel (DDP) strategies. Regarding memory usage, a GPU with 48GB or 24GB of memory is sufficient, and the peak memory usage reached 341.5GB when loading all motif scores and labels for 104 cell lines/tissues into memory to enhance dataloader efficiency. To lower the requirement for memory usage during training, sample data can be stored on disk and loaded in batches, rather than loading all data into memory at once. This approach significantly reduces memory usage, making it feasible to train the model on most standard computing systems.

# Supplementary Figures

## Fig. S1

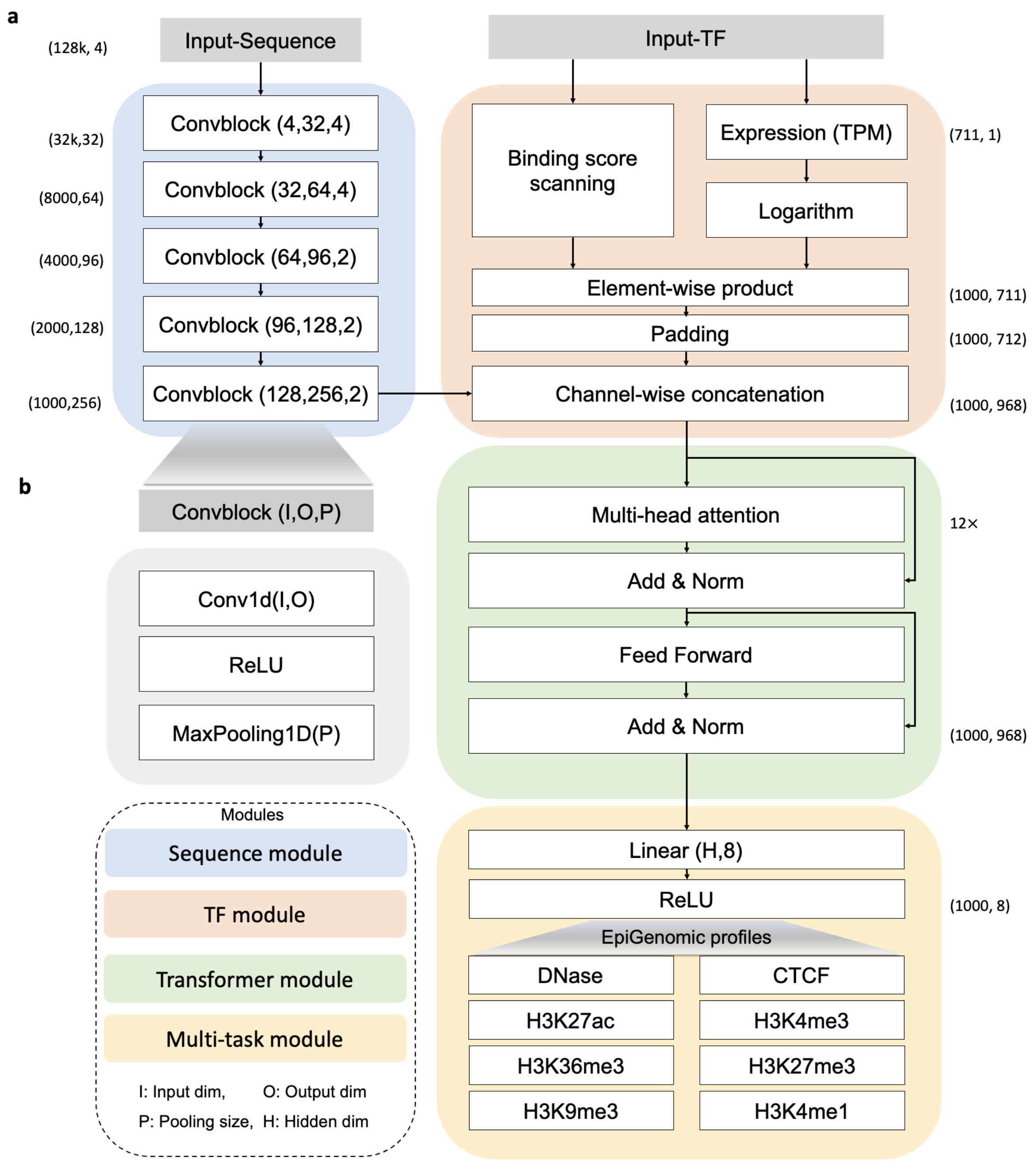


**Fig S1. Model architecture of EpiGePT for multiple epigenomic signals prediction. a**, The computational process of EpiGePT. The sequence module employs a stack of five convolutional layers followed by pooling operations, resulting in representations that capture sequence patterns. The TF module integrates motif binding information and gene expression data to represent cell-specific

information. The Transformer module takes the genomic bin sequences mentioned above as input and learns the interaction relationships between bins, capturing the interactions among them. Finally, the obtained embeddings are mapped to the eight types of epigenomic signals through a fully connected layer. **b**, Specific details of the convolutional block involve the fusion of 1D convolution, ReLU activation function, and max pooling operation to achieve changes in the feature dimension O and extract bin-level features.

## Fig. S2

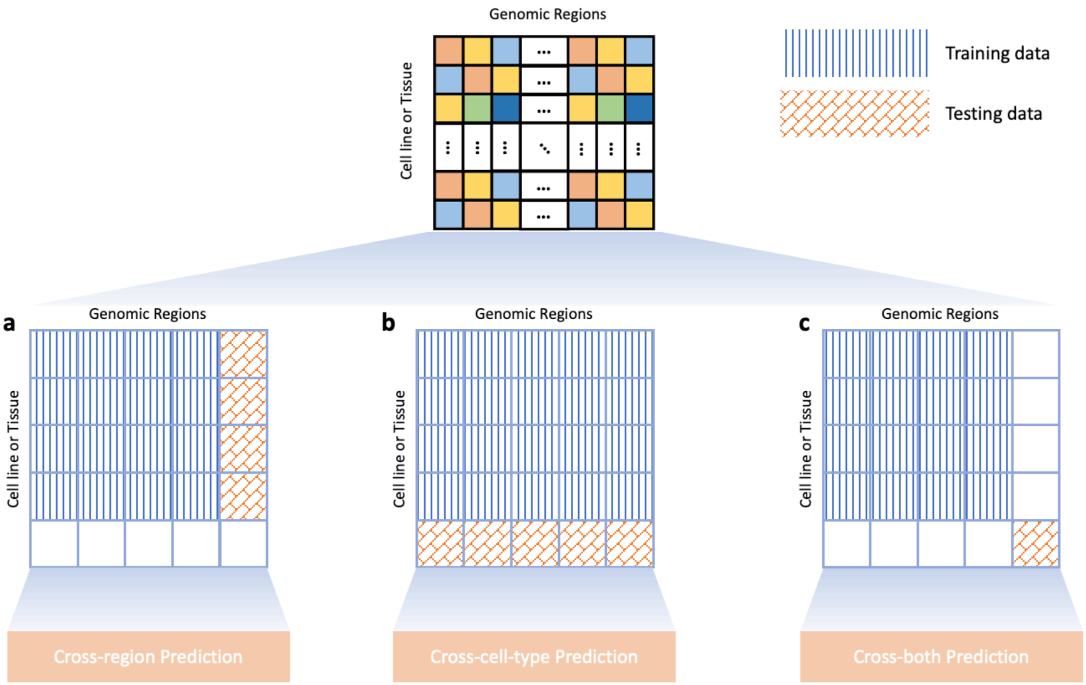


**Fig. S2. Three data partitioning strategies for model training and testing. a**, Cross genomic region prediction. The training and testing datasets utilized the expression profiles of identical cell types, but were evaluated on novel genomic regions for prediction. **b**, Cross cell type prediction. The training and testing datasets utilized the same genomic regions, but were evaluated on novel cell types for prediction. **c**, Cross genomic region and cell type prediction. The cell types and genomic regions used in the training and test sets were both different.

# Fig. S3

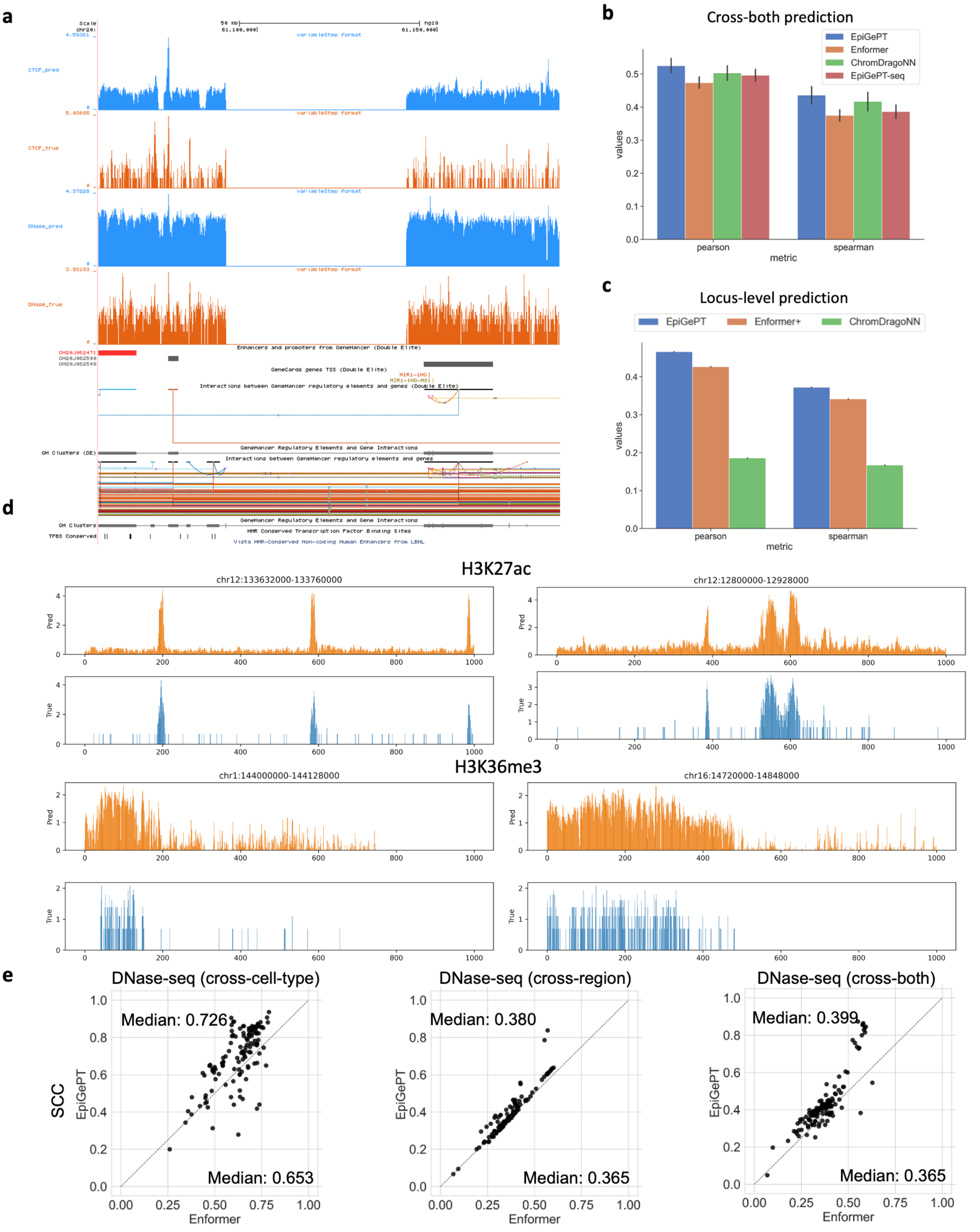


**Fig. S3. EpiGePT's performance in predicting DNase-seq and other epigenetic signals is demonstrated in a**, through visualization of predicted results for DNase and CTCF signals. EpiGePT

is able to make accurate predictions for these signals, as well as for the regulatory relationships within a genomic region of 20th chromosome ranging from 61,100,000 to 61,150,000. **b**, EpiGePT and baseline methods were compared for their performance in predicting epigenetic signals in new cell types and genomic regions (cross-both prediction). The left panel shows the Pearson correlation coefficient, and the right panel shows the Spearman correlation coefficient. **c**, Locus level prediction of DNase signal. We predicted a value for each genomic locus, and calculated the correlation coefficient between the predicted values and true values for the same locus in different cell types. **d**, Visualization of predicted signals, such as the comparison between predicted and true values in a 128kbp region (from 133,632,000 to 133,760,000) on chromosome 12, shows that the presence of a large number of zeros in both the true and predicted signals can limit the correlation between the two signals. **e**, Comparison of EpiGePT and Enformer performance. Each point in the scatter plot represents the performance of Enformer on the data of a specific cell type (x-axis) compared to the performance of EpiGePT (y-axis). The three graphs represent the prediction of continuous DNase-seq signals (spearman correlation coefficient).

## Fig. S4

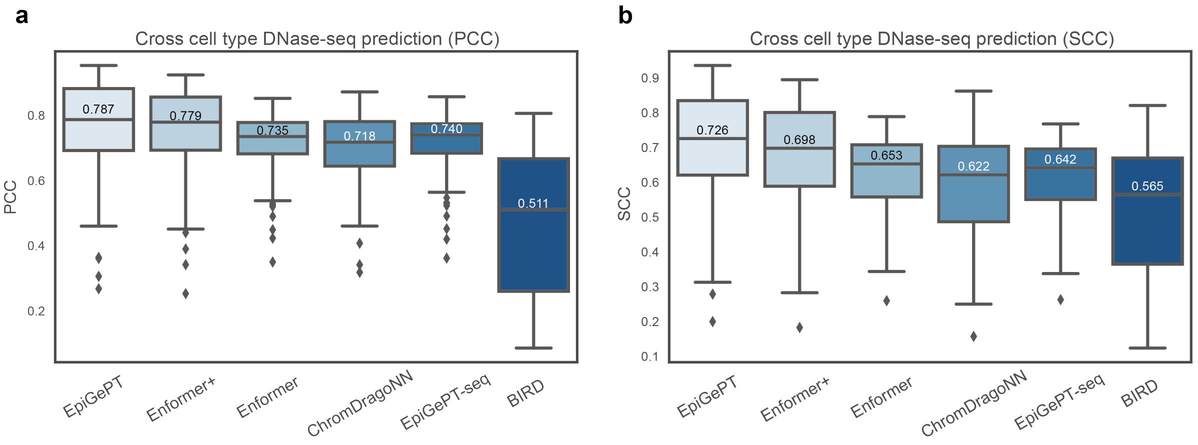


**Fig. S4. Comparison of cross-cell-type chromatin accessibility (DNase-seq profiles) prediction performance between EpiGePT and baseline methods. a**, Box plot of Pearson correlation coefficients (PCC) of the 5-fold cross-validation results. **b**, Box plot of Spearman correlation coefficients (SCC) of the 5-fold cross-validation results.

# Fig. S5

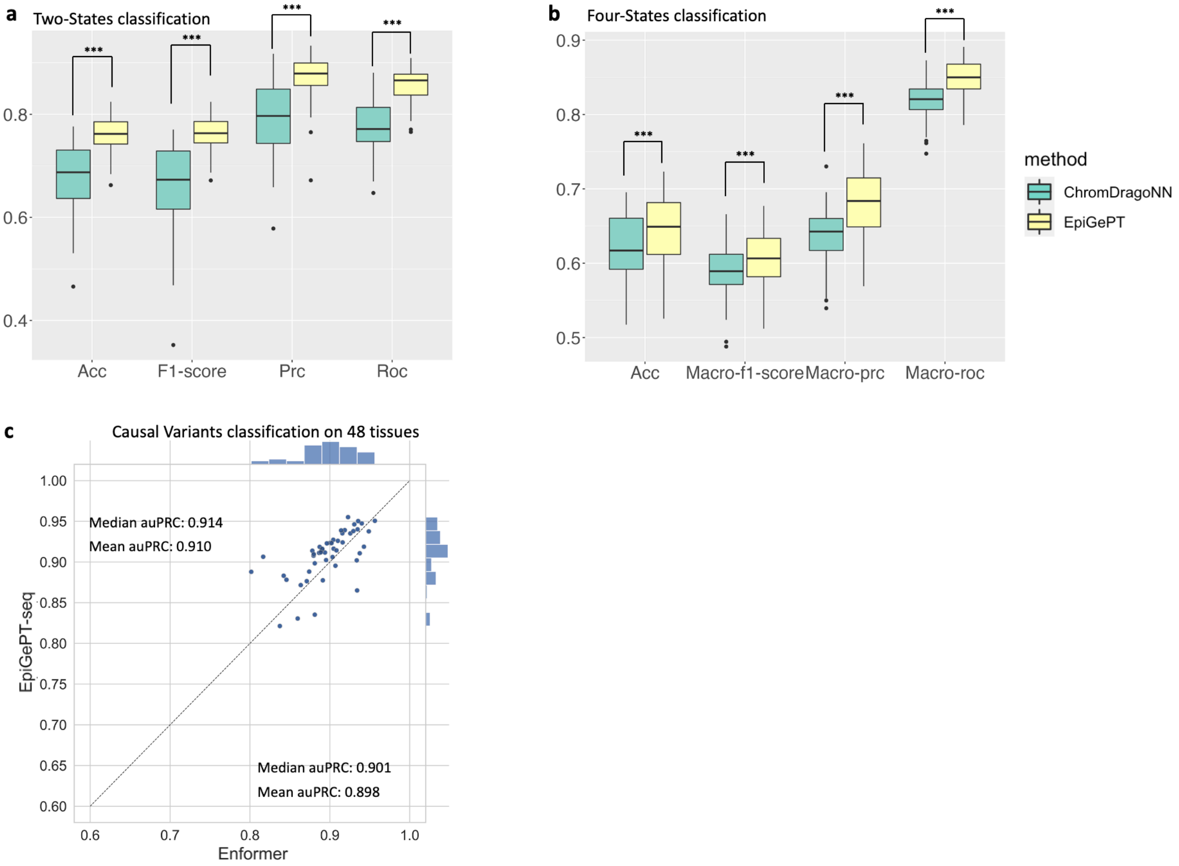


**Fig. S5. Performance of EpiGePT and baseline methods on chromatin states classification and causal variants classification. a**, Binary classification of chromatin states for distinguishing functional regions on the chromatin based on the annotation data from ChromHMM-15-states. **b**, Four-class chromatin state classification is used to distinguish functional regions on the chromatin, including TSS, potential enhancers, other functional regions, and non-functional regions based on the annotation data from ChromHMM-15-states. *** indicates that the *p*-value is less than 1e-3 under one-sided Wilcoxon signed rank test. **c**, The performance of EpiGePT and Enformer in discriminating causal eQTLs across 48 tissues, each dot representing the average auPRC obtained from 5-fold cross-validation on a specific tissue.

## Fig. S6

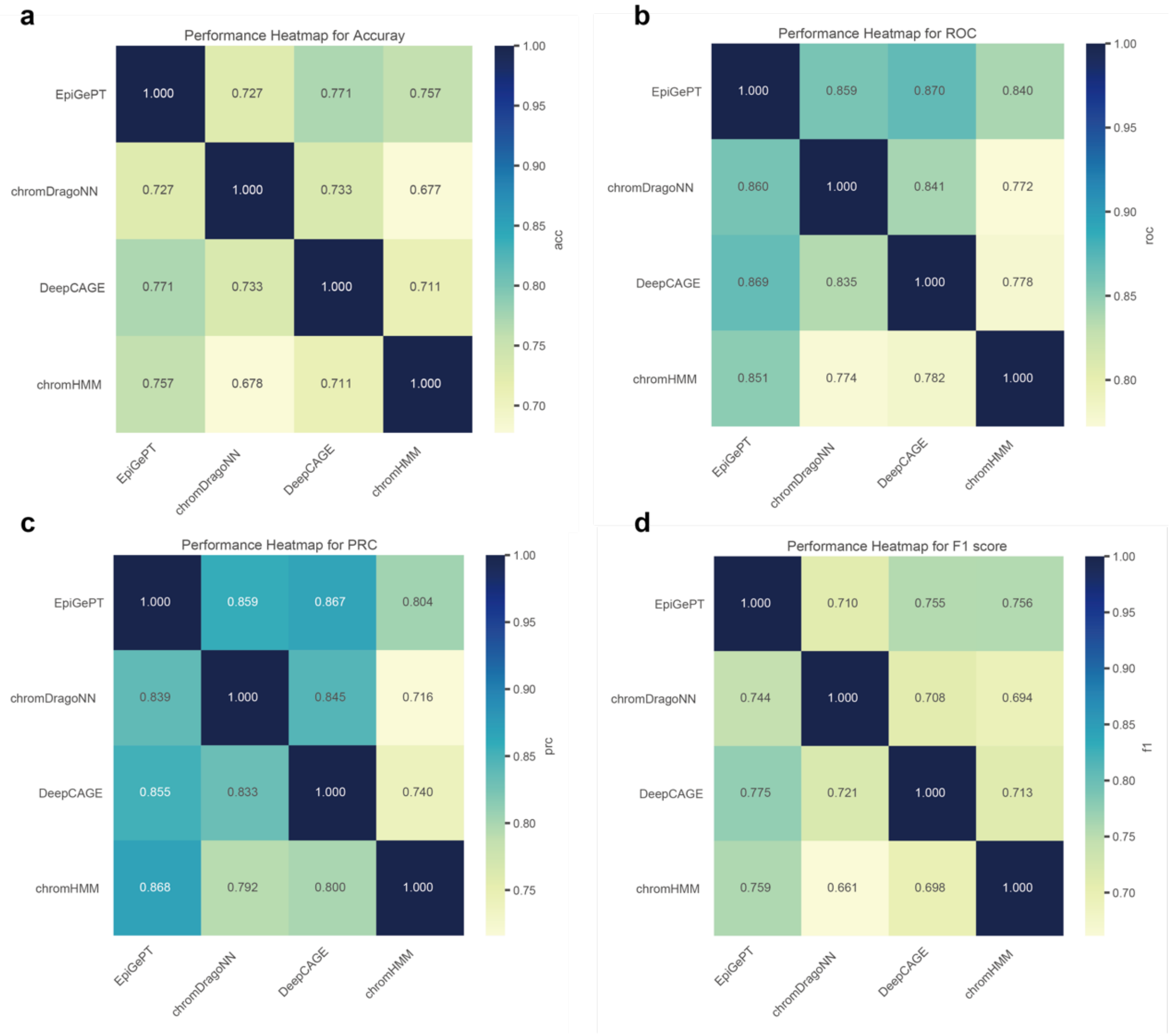


**Fig. S6. The average predictive performance of the remaining methods when each model serves as the ground truth. a**, Heatmap of average accuracy when the four methods serve as ground truth. The rows represent the ground truth, the columns represent the prediction methods, and the values indicate the five-fold cross-validation averages across all test cell types. **b**, Heatmap of average auROC when the four methods serve as ground trut2h. **c**, Heatmap of average auPRC when the four methods serve as ground truth. **d**, Heatmap of average f1-score when the four methods serve as ground truth.

## Fig. S7

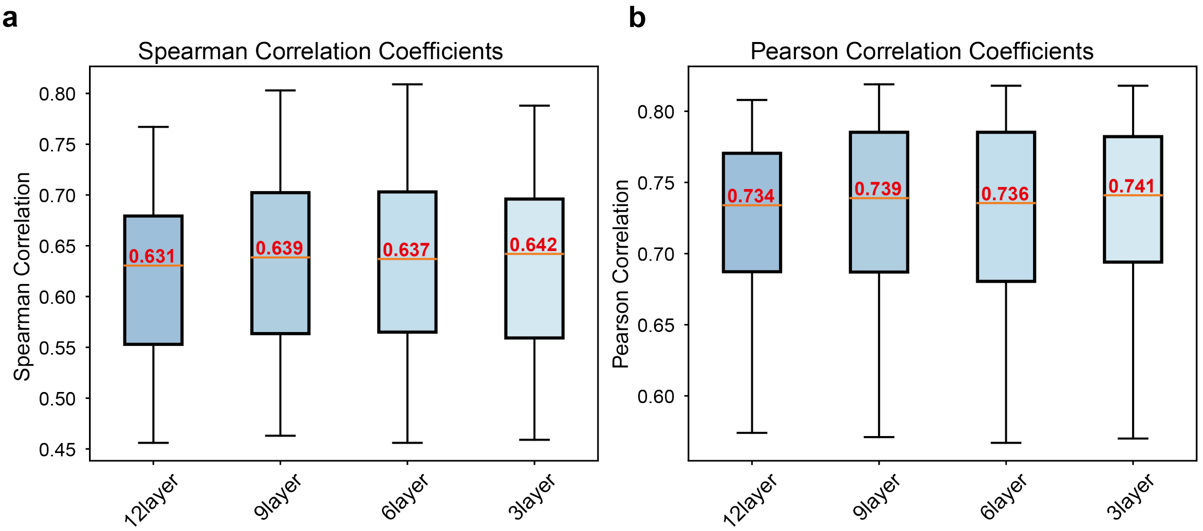


**Fig. S7. The performance comparison of retrained Enformer with different numbers of encoder layers for predicting chromatin accessibility.** **a**, The boxplot of PCC for predicting chromatin accessibility across 26 test cellular contexts by Enformer with varying numbers of encoder layers (3, 6, 9, and 12). **b**, The boxplot of SCC for predicting chromatin accessibility across 26 test cellular contexts by Enformer with varying numbers of encoder layers (3, 6, 9, and 12).

# Fig. S8

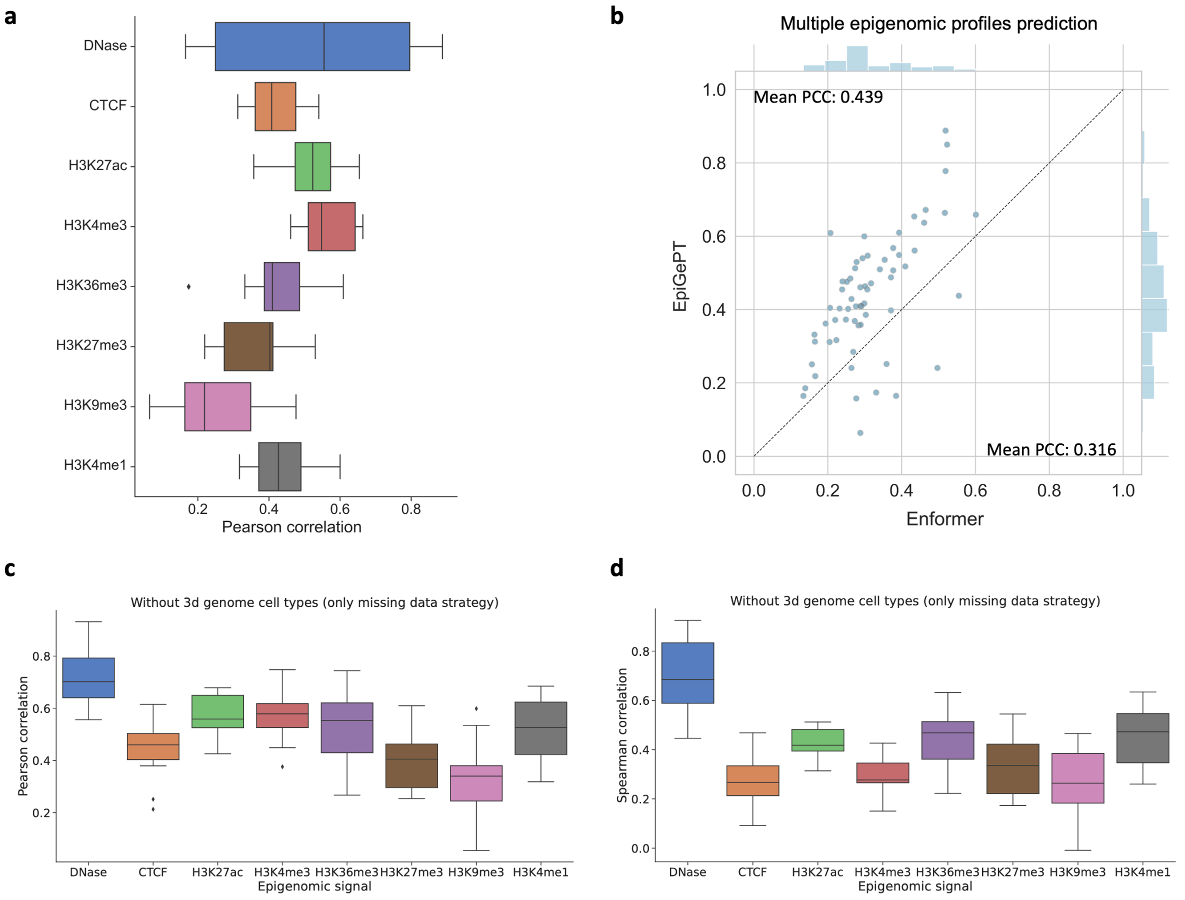


**Fig. S8. Performance of EpiGePT in cross-cell-type prediction. a**, The predictive performance of EpiGePT on 8 unseen cell types on hg19 reference genome (pearson correlation coefficients). **b**, Cross-cell-type prediction of 8 epigenomic signals at 8 test cell types. Each dot denotes the Pearson correlation coefficient of the predicted signals and true signals at the specific cell types on a specific epigenomic signal. **c**, The predictive performance of EpiGePT on 19 new cell types on hg38 reference genome (PCC). **d**, The predictive performance of EpiGePT on 19 new cell types on hg38 reference genome (SCC).

## Fig. S9

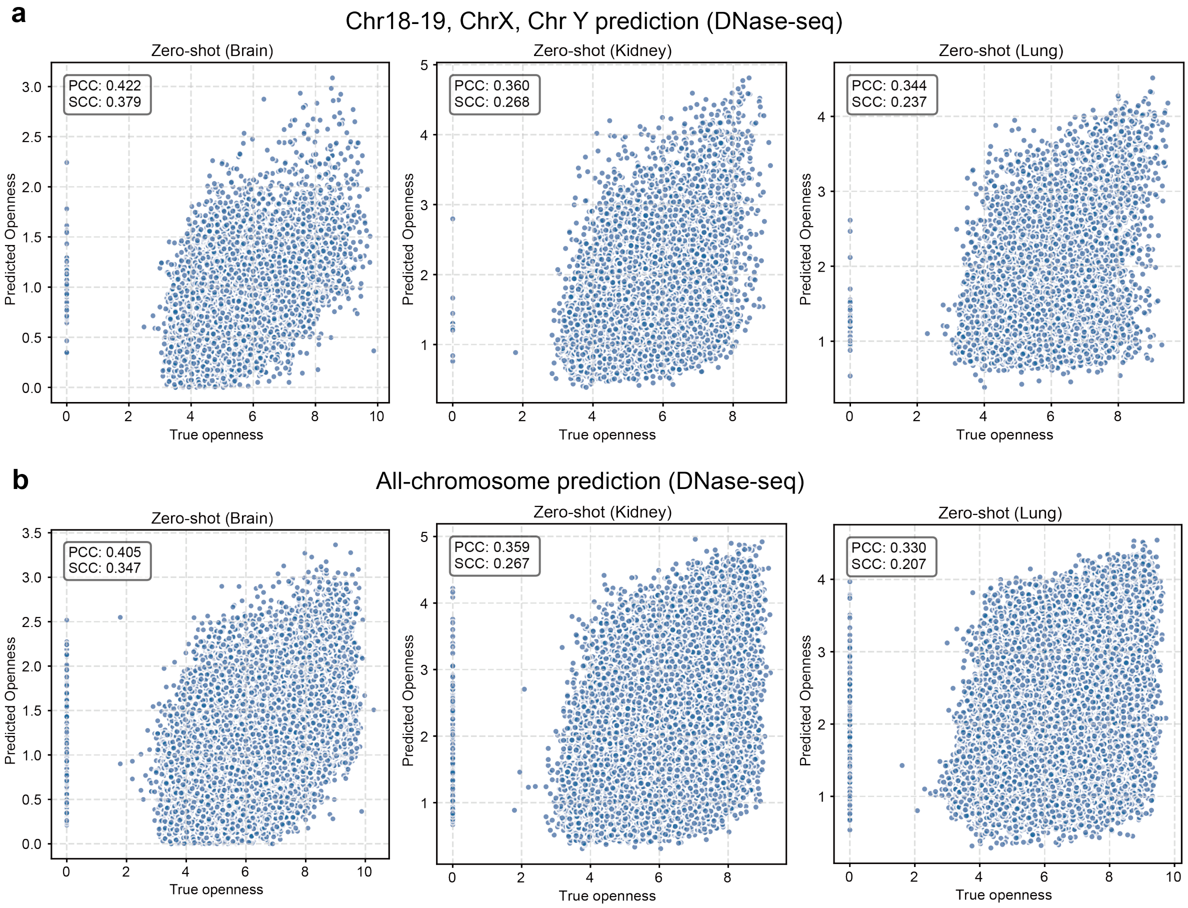


**Fig. S9. Zero-shot learning of EpiGePT on chromatin accessibility (DNase-seq) prediction on mouse data. a**, Scatter plots depicting the predicted openness and ground truth (logarithmically transformed foreground read count) for peaks on chromosomes 18-19 as well as the X and Y chromosomes for the brain (22,750 regions), kidney (16,250 regions), and lung (22,450 regions). **b**, Scatter plots depicting the predicted openness and ground truth (logarithmically transformed foreground read count) for peaks on all chromosomes for the brain (247,750 regions), kidney (180,400 regions), and lung (243,900 regions).

# Fig. S10

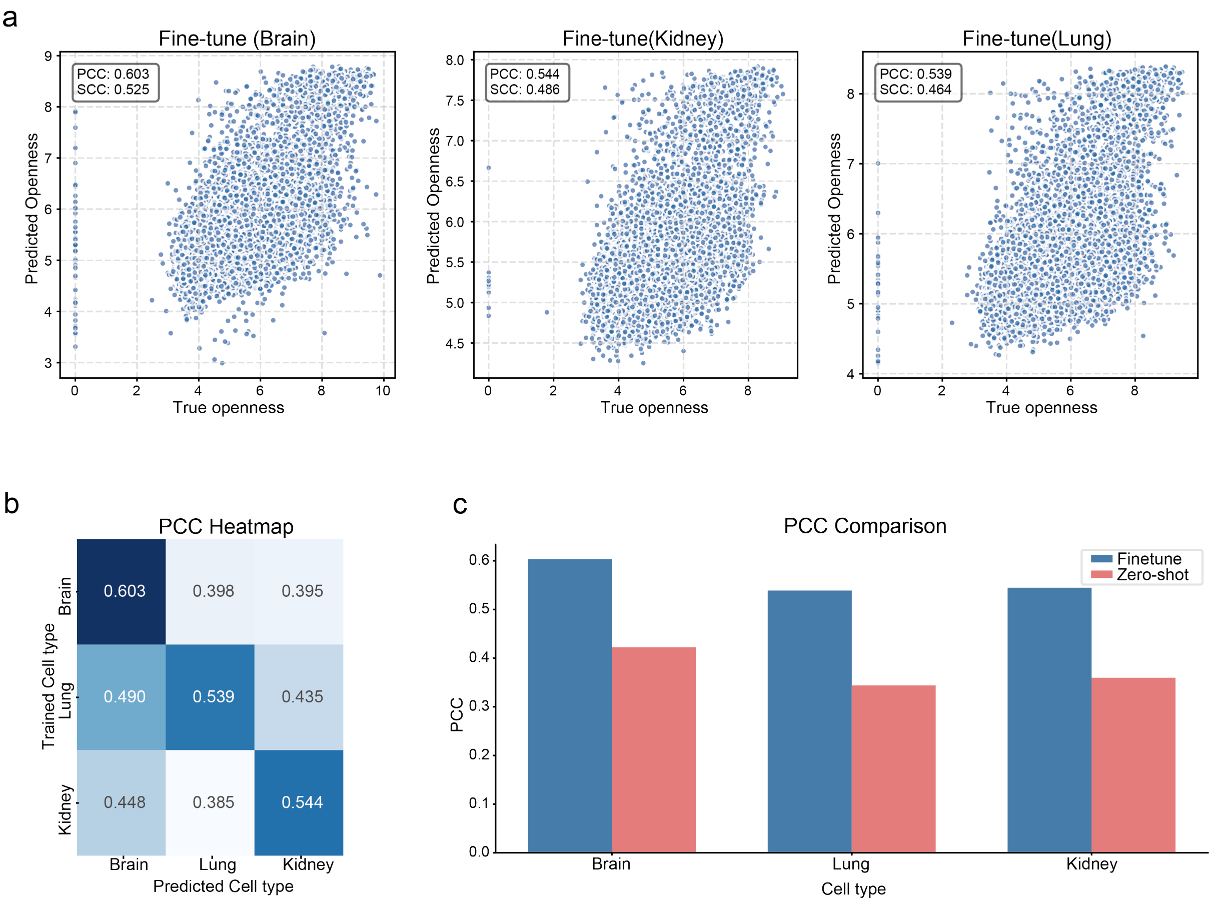


**Fig. S10. Model fine-tuning EpiGePT on chromatin accessibility (DNase-seq) prediction on mouse data. a**, Scatter plots depicting the predicted openness and ground truth (logarithmically transformed foreground read count) using fine-tuned model for peaks on chromosomes 18-19 as well as the X and Y chromosomes for the brain, kidney, and lung tissues. **b**, The heatmap matrix represents the performance across different cell types, with columns indicating the predicted cell types and rows indicating the training cell types. **c**, The comparison of performance (PCC) between finetuned and zero-shot models for predicting peaks on chromosomes 18-19 and the X and Y chromosomes across brain, kidney, and lung cell types shows the performance differences after finetuning.

# Fig. S11

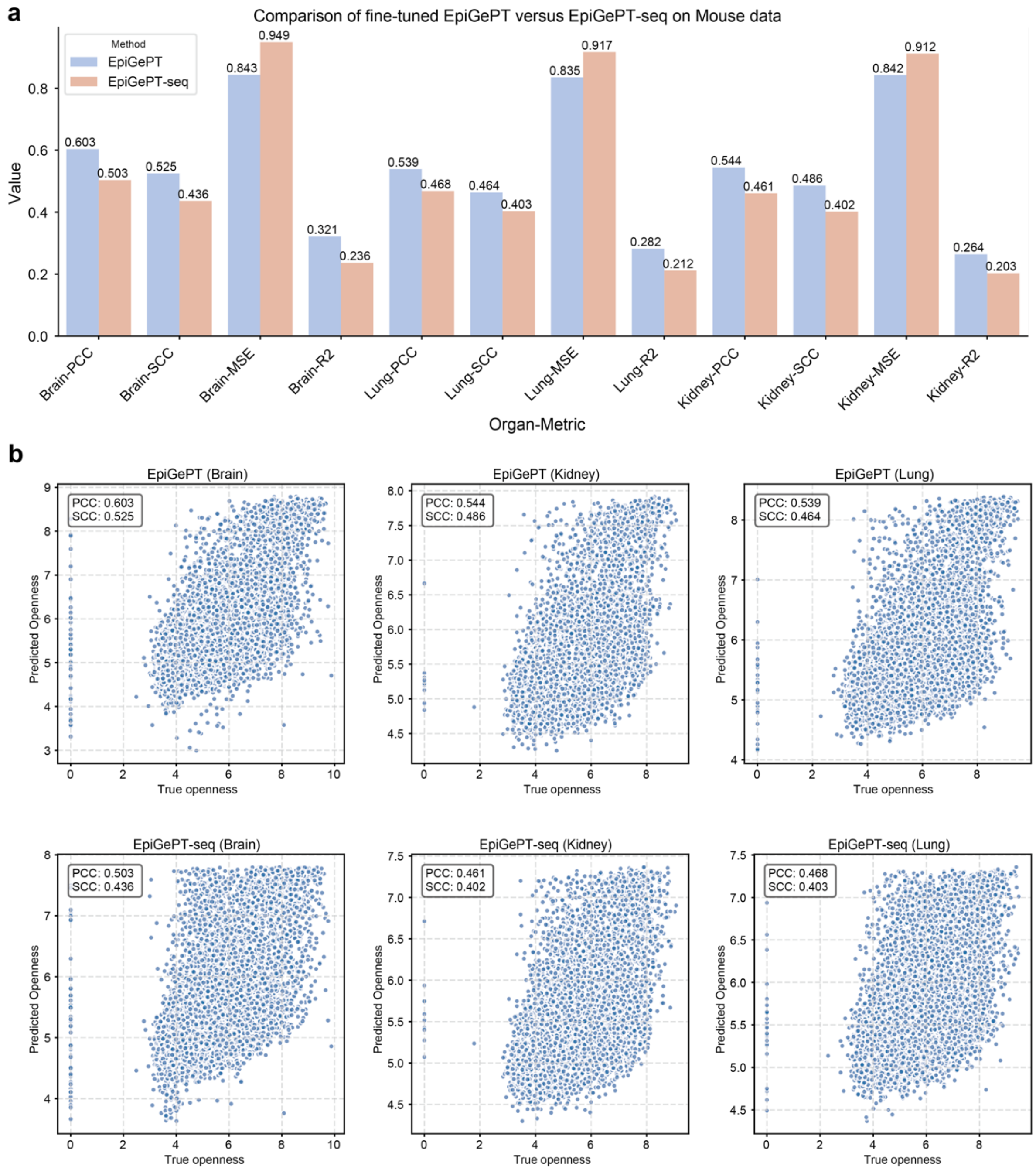


**Fig. S11. The cross-chromosome predictive performance of pretrained EpiGePT and EpiGePT-seq after fine-tuning on mouse data. a**, The bar plot illustrates the Pearson correlation coefficient (PCC), Spearman correlation coefficient (SCC), mean squared error (MSE), and R square between the predicted and true chromatin openness for EpiGePT and EpiGePT-seq, across 22,750 bins in the brain, 22,450 bins in the lung, and 16,200 bins in the kidney. **b**, The scatter plots illustrate the relationship

between the predicted and true chromatin openness for EpiGePT and EpiGePT-seq, across 22,750 bins in the brain, 22,450 bins in the lung, and 16,200 bins in the kidney.

## Fig. S12

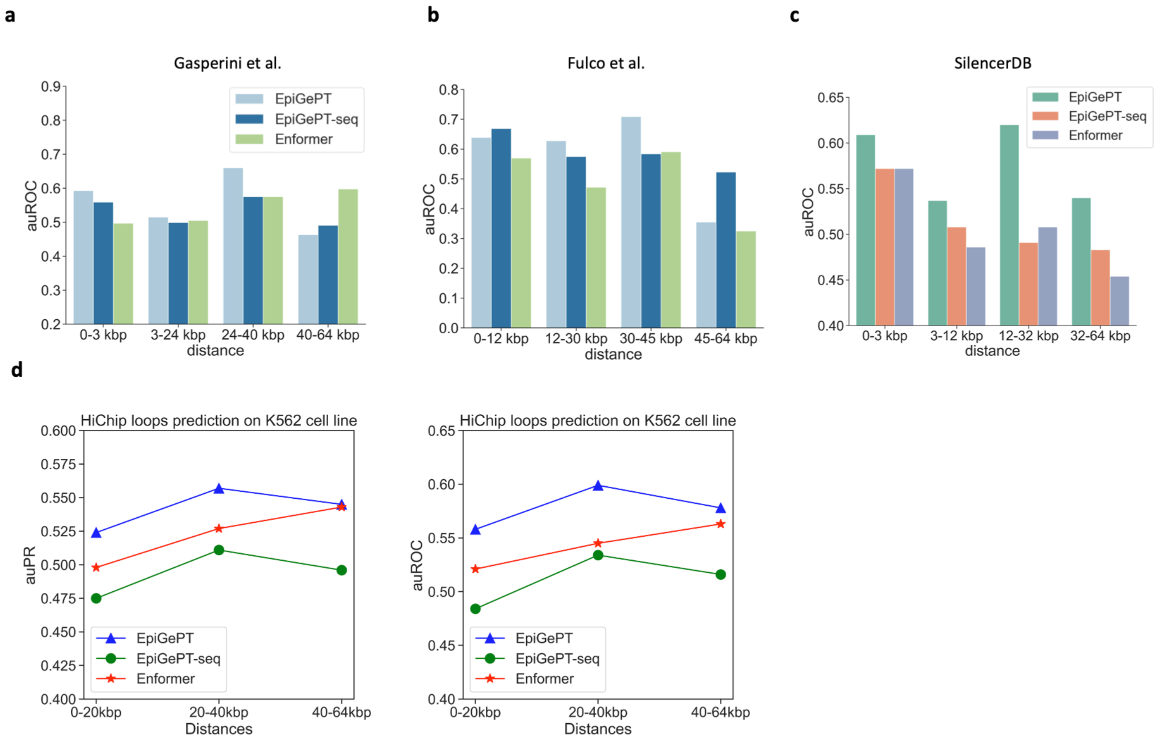


**Fig. S12. The performance (auROC) of attention score of EpiGePT in distinguishing regulatory element-gene pairs at different distance ranges. a**, The performance of EpiGePT in distinguishing enhancer-gene pairs at different distance ranges on the data from Gasperini et al. (15). **b**, The performance of EpiGePT in distinguishing enhancer-gene pairs at different distance ranges on the data from Fulco et al. (16). **c**, The performance of EpiGePT in distinguishing silencer-promoter pairs at different distance ranges on the data from SilencerDB (17). **d**, The performance (auROC and auPR) of attention score of EpiGePT in distinguishing HiChIP loops of H3K27ac at different distance ranges on K562 cell line.

## Fig. S13

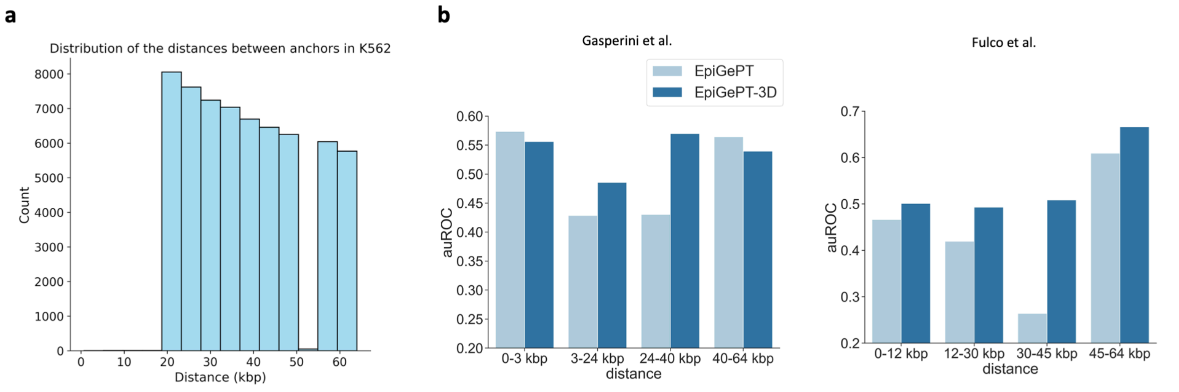


**Fig. S13. Incorporating 3D genomic information from HiChip data enhances the predictive performance of EpiGePT on E-P regulatory interaction on K562 cell line. a**, The distance distribution between the two anchors of the filtered loops on the K562 cell line. **b**, The performance (auROC) of self-attention scores of EpiGePT and EpiGePT-3D in identifying enhancer-promoter interactions across different distance ranges on the K562 cell type.

## Fig. S14

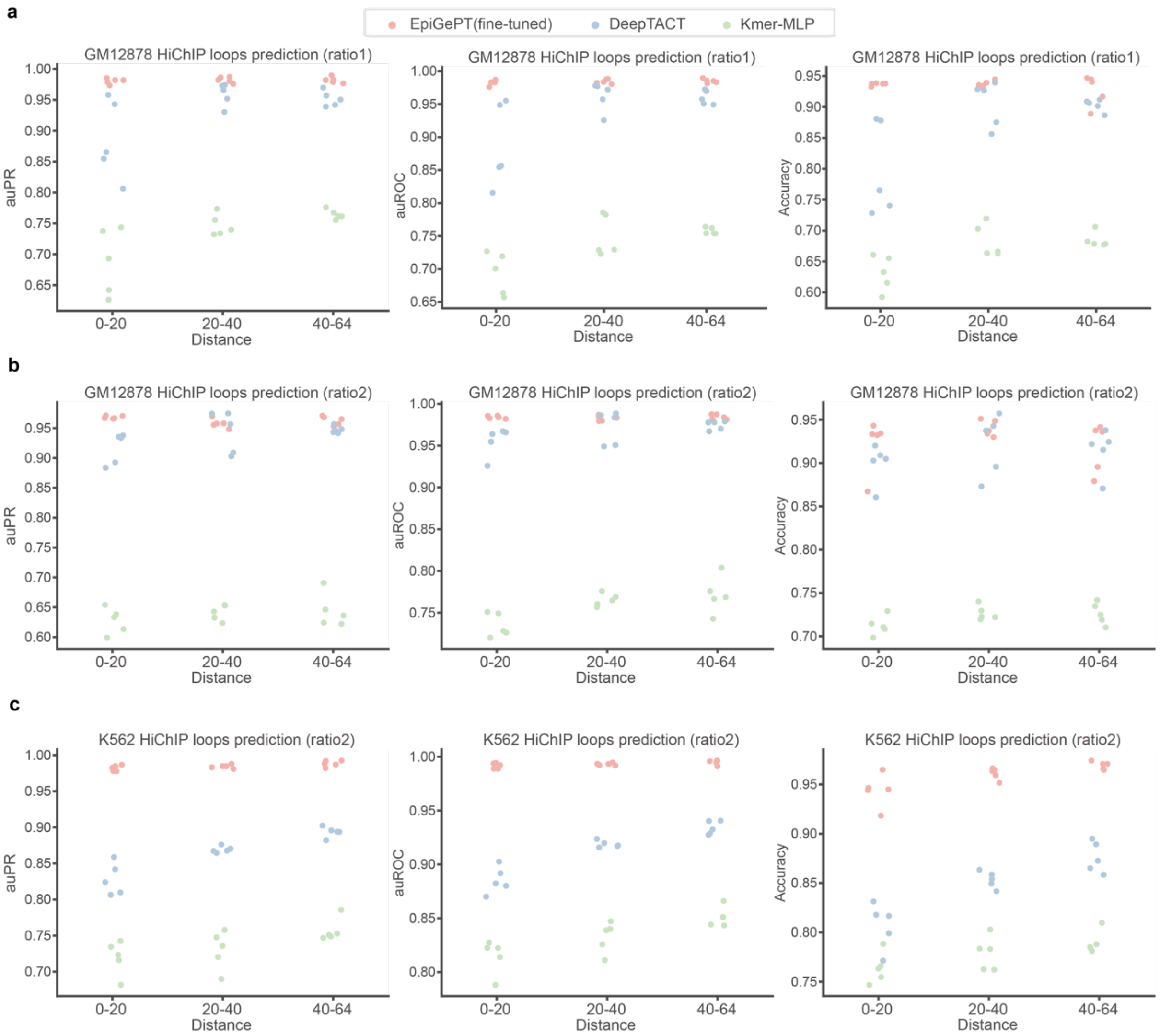


**Fig. S14. The fine-tuning performance of the EpiGePT model on predicting potential enhancer-promoter regulatory networks. a**, The performance (measured by auROC and auPRC) of the fine-tuned EpiGePT model and baseline methods (DeepTACT and Kmer-MLP) on HiChIP loops data (18) in distinguishing enhancer-gene pairs at various distance ranges (0-20 kbp, 20-40 kbp and 40-64 kbp). **b**, The performance of the fine-tuned EpiGePT model and baseline methods on HiChIP loops data in distinguishing enhancer-gene pairs under 1:2 positive-negative sample ratio on GM12878 cell line. **c**, The performance of the fine-tuned EpiGePT model and baseline methods on HiChIP loops data in distinguishing enhancer-gene pairs under 1:2 positive-negative sample ratio on K562 cell line.

## Fig. S15

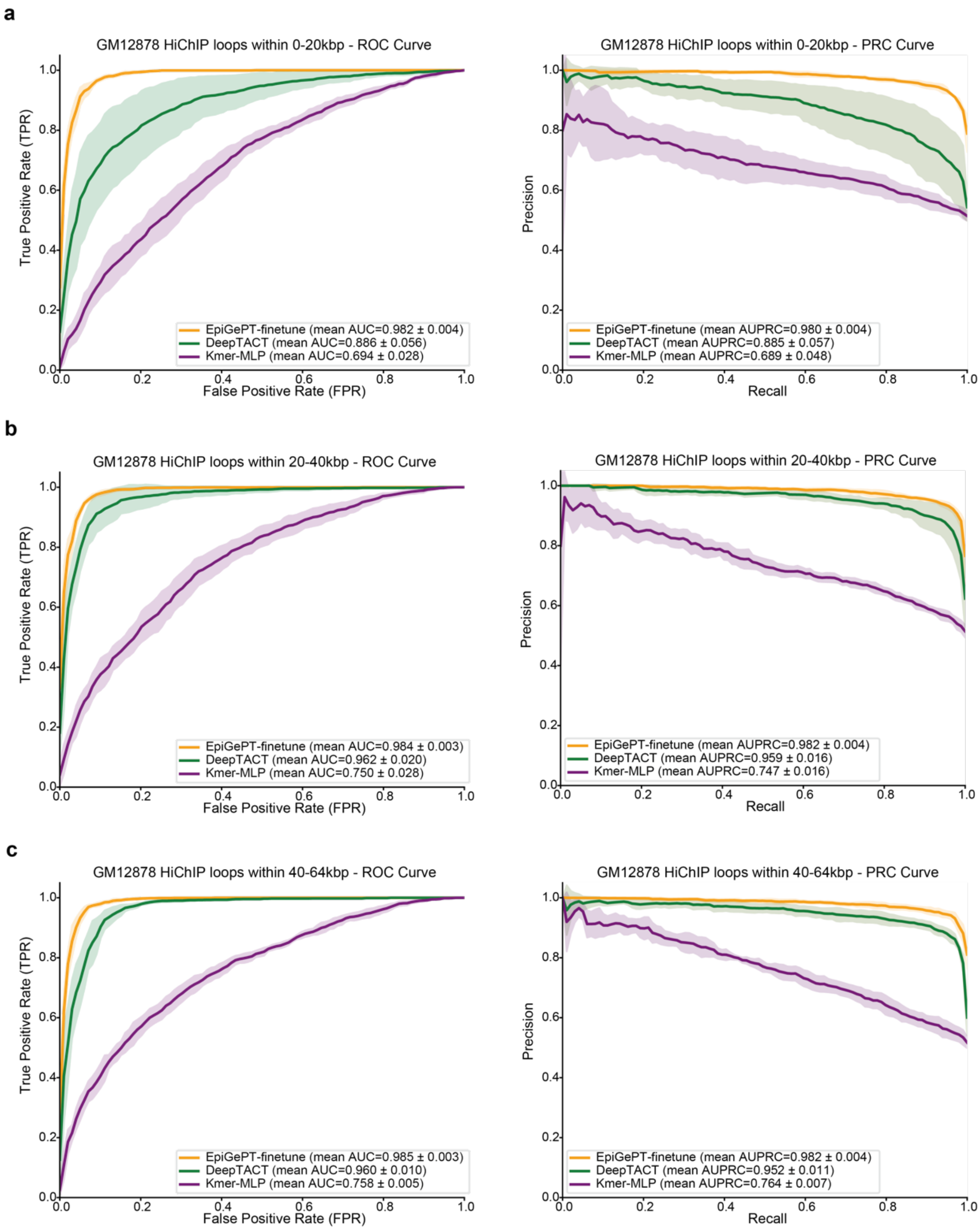


**Fig. S15. The ROC and PR curves of the EpiGePT model on predicting potential enhancer-promoter regulatory networks. a**, The ROC and PR curves (5-fold cross validation) of EpiGePT-finetune and baseline methods for predicting HiChIP loops from the GM12878 cell line (0-20 kbp). **b**, The ROC and PR curves (5-fold cross validation) of EpiGePT-finetune and baseline methods for predicting HiChIP loops from the GM12878 cell line (20-40 kbp). **c**, The ROC and PR curves (5-fold

cross validation) of EpiGePT-finetune and baseline methods for predicting HiChIP loops from the GM12878 cell line (40-64 kbp).

# Fig. S16

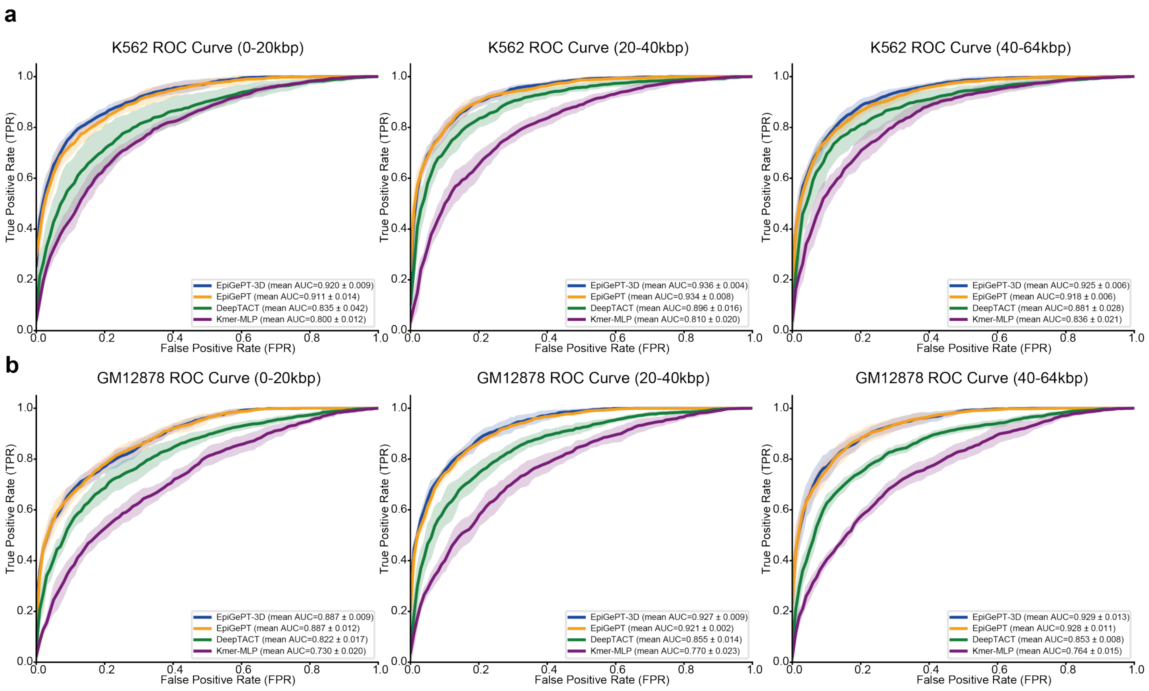


**Fig. S16. Comparison of the performance between fine-tuned EpiGePT-3D, EpiGePT and baseline methods in predicting HiChIP loops (ROC curves).** **a**, ROC curves (5-fold cross validation) comparing the performance of EpiGePT-3D, EpiGePT and baseline methods in predicting chromatin interactions (HiChIP loops) on the K562 cell line (positive-to-negative sample ratio of 1:1). **b**, ROC curves (5-fold cross validation) comparing the performance of EpiGePT-3D, EpiGePT and baseline methods in predicting chromatin interactions (HiChIP loops) on the GM12878 cell line (positive-to-negative sample ratio of 1:1).

# Fig. S17

**a**

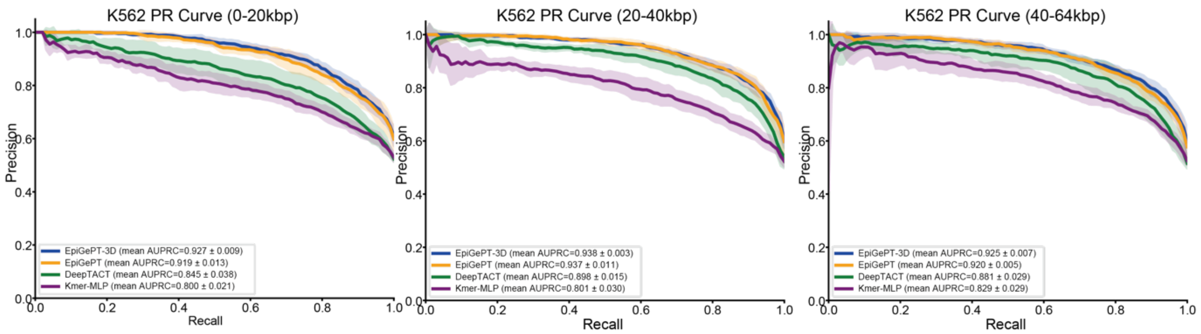


**b**

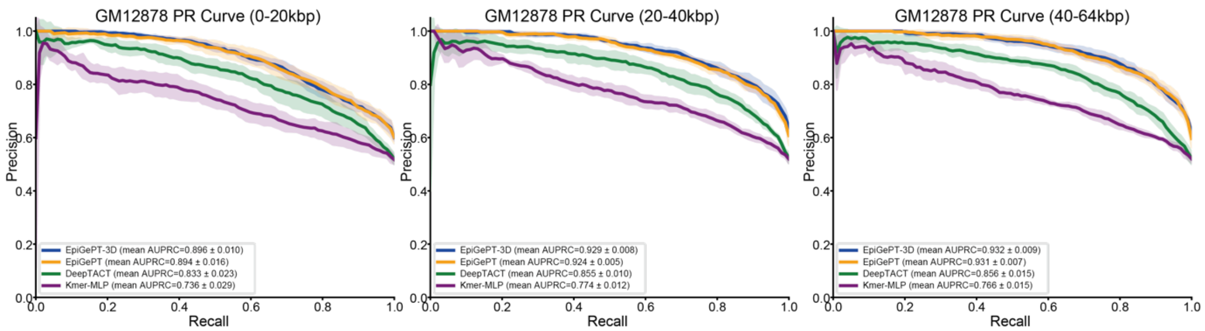


**Fig. S17. Comparison of the performance between fine-tuned EpiGePT-3D, EpiGePT and baseline methods in predicting HiChIP loops (PR curves)**. **a**, PR curves (5-fold cross validation) comparing the performance of EpiGePT-3D and baseline methods in predicting chromatin interactions (HiChIP loops) on the K562 cell line (positive-to-negative sample ratio of 1:1). **b**, PR curves (5-fold cross validation) comparing the performance of EpiGePT-3D, EpiGePT and baseline methods in predicting chromatin interactions (HiChIP loops) on the GM12878 cell line (positive-to-negative sample ratio of 1:1).

## Fig. S18

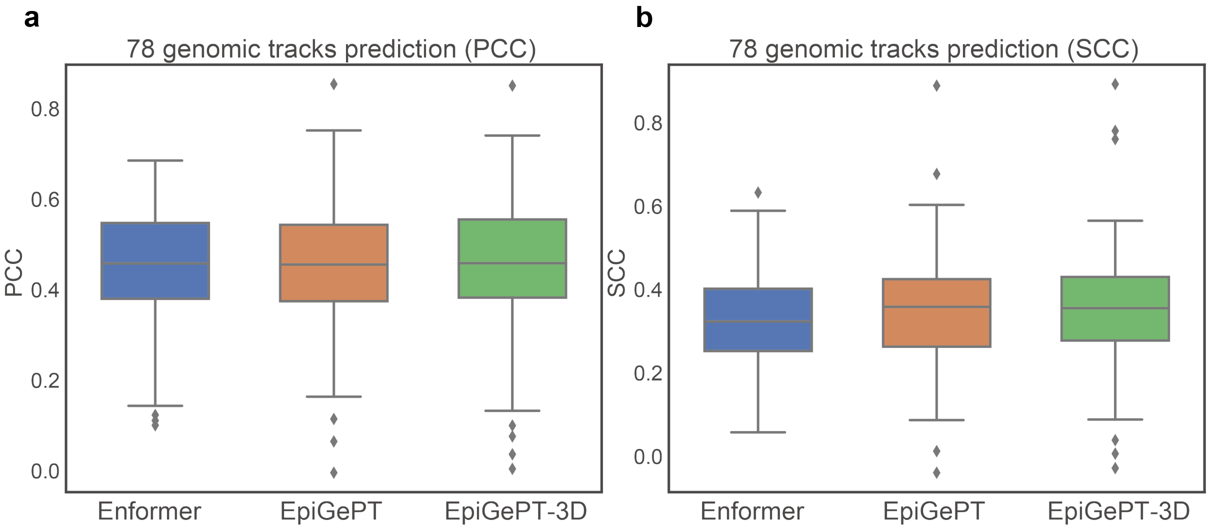


**Fig. S18. The performance comparison of original Enformer with EpiGePT and EpiGePT-3D on 78 genomic tracks. a**, The boxplot of PCC for the prediction of 78 genomic tracks across 15,830,000 non-overlapping 128bp bins by the original Enformer, EpiGePT, and EpiGePT-3D (trained on 11 cellular contexts and weight α for 3d genome loss was set to 1). **b**, The boxplot of SCC for the prediction of 78 genomic tracks across 15,830,000 non-overlapping 128bp bins by the original Enformer, EpiGePT, and EpiGePT-3D.

# Fig. S19

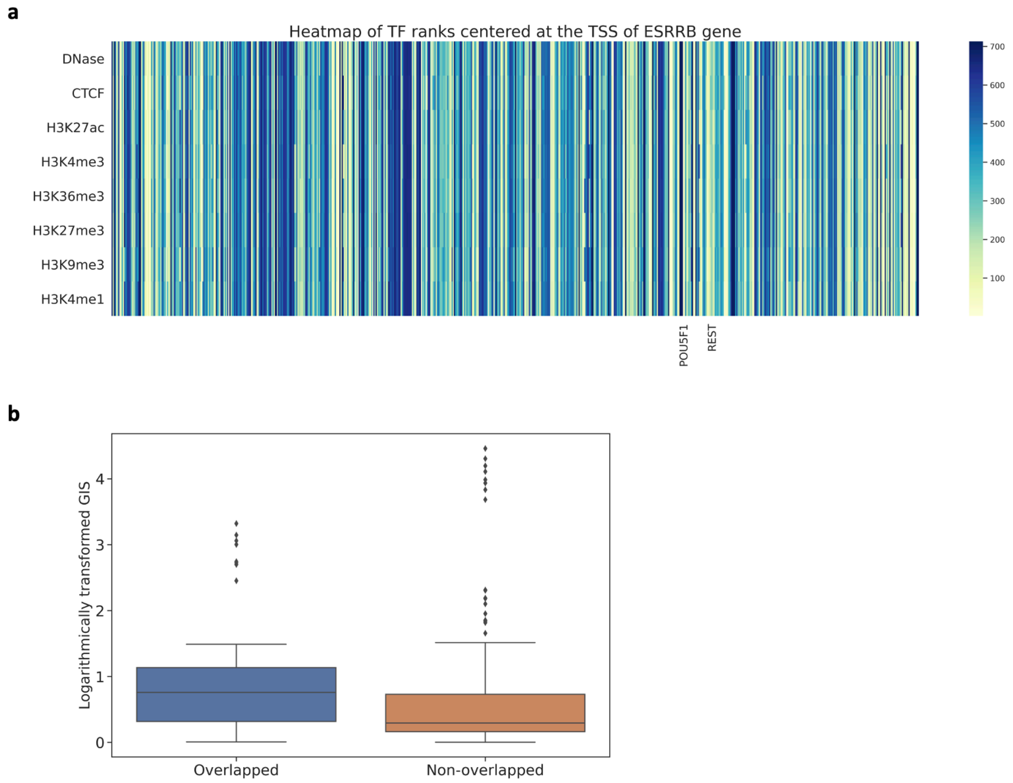


**Fig. S19. The GIS of ChIP-seq overlapped bins versus non-overlapped bins of *POU5F1* centered at the TSS of *ESRRB*. a**, Heatmap of TF ranks across 128 kbp region surrounding the TSS of *ESRRB* gene, each row denotes an epigenomic signal and each column denotes a TF. **b**, Distribution of non-zero GIS values on overlapped and non-overlapped bins in chip-seq data (ENCFF696NWL).

# Fig. S20

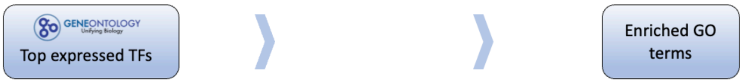


| GO Term ID | Biological process | P-value | FDR |
|---|---|---|---|
| GO:0048598 | embryonic morphogenesis | 8.78e-07 | 1.98e-04 |
| GO:0009790 | embryo development | 1.92e-07 | 4.99e-05 |
| GO:0048568 | embryonic organ development | 1.06e-07 | 2.86e-05 |
| GO:0009792 | embryo development ending in birth or egg hatching | 1.95e-04 | 1.79e-02 |
| GO:0030154 | cell differentiation | 6.67e-07 | 1.58e-04 |
| GO:0001892 | embryonic placenta development | 2.09e-05 | 2.92e-03 |

**Fig. S20. Gene ontology enrichment analysis based on the top 5% TFs with high expression in ESCs.** The results showed lower significance for biological processes associated with embryonic cell development compared with GO terms enriched with the top 5% ranked TFs.

## Fig. S21

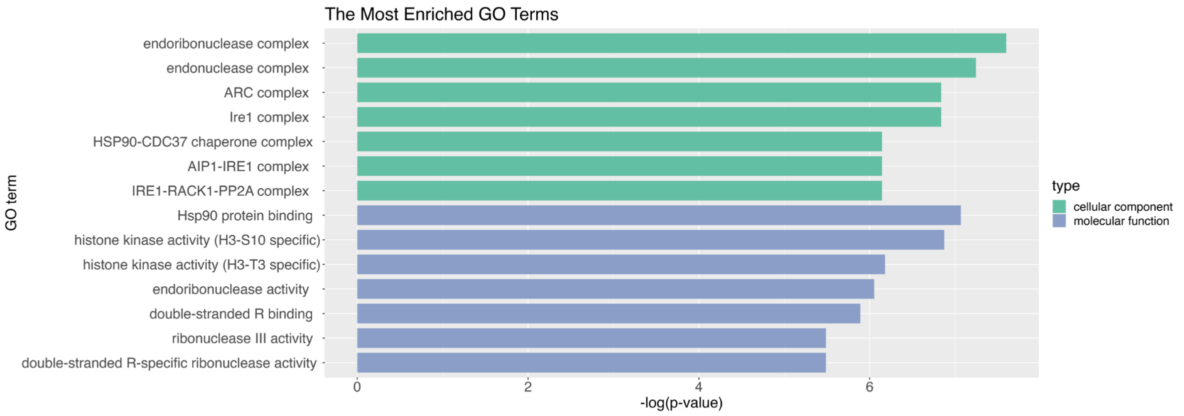


**Fig. S21. Enrichment result (Cellular component and Molecular function) of the nearest genes of the COVID-19 associated SNPs with the low LOS.**

# Fig. S22

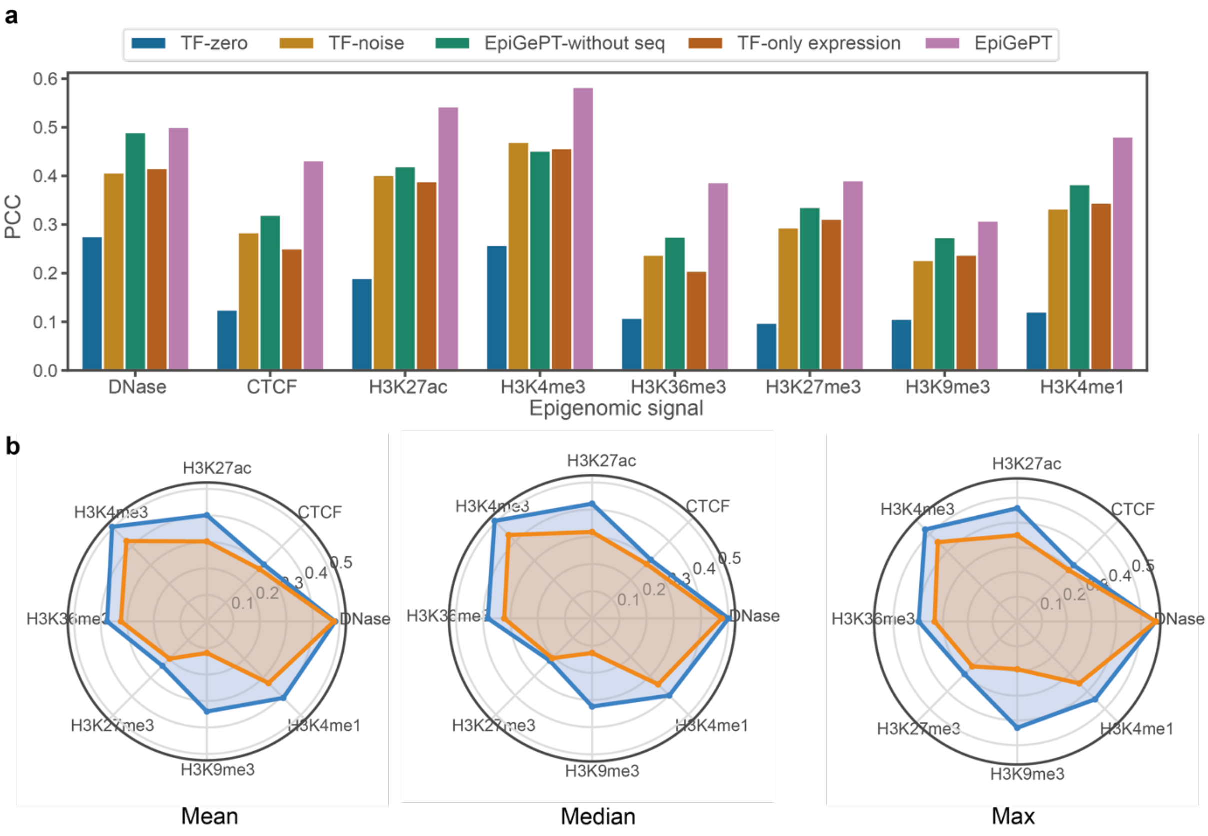


**Fig. S22. Ablation analysis of the EpiGePT model. a**, Ablation analysis on the TF module and the Sequence module, we observed a decrease in predictive performance for each module across eight chromatin epigenetic signals, as evidenced by a reduction in Pearson correlation coefficient. **b**, Ablation analysis on the Multi-task module. The green shaded area in the figure represents the results of multi-signal cross-cell-type predictions, while the red shaded area represents the results of training and predicting on each signal individually. It can be observed that the multi-task module has a positive effect on the model performance across all signals.

# Fig. S23

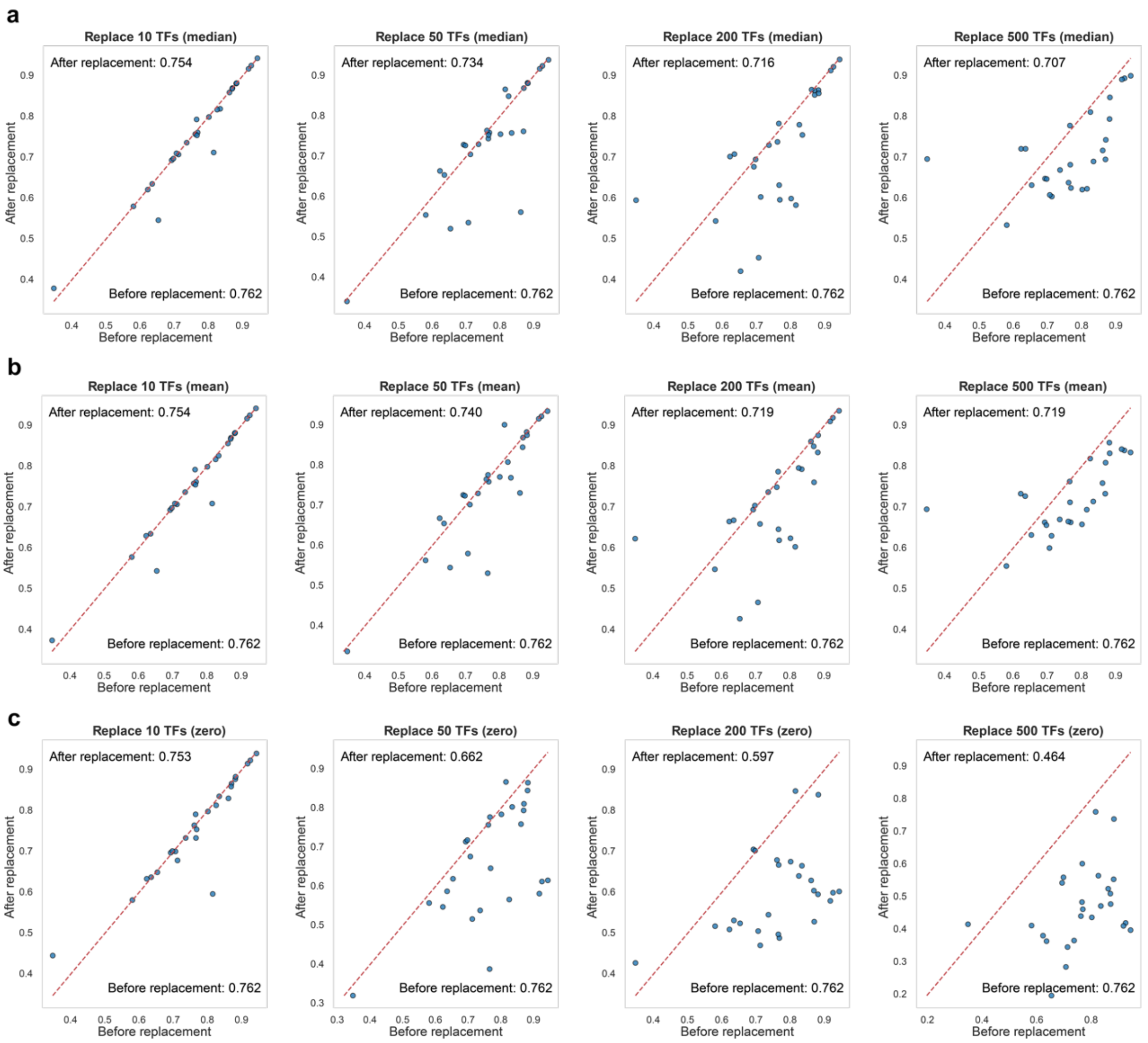


**Fig. S23. The simulation evaluated the changes in PCC for cross-cell type predictions under different numbers of missing TF profiles with three replacement strategies. a**, The average PCC for 26 test cell lines/tissues after replacing different numbers (10, 50, 200, 500) of TF profiles with the median value of the reference profile. In the scatter plot, the x-axis represents the performance before replacement, and the y-axis represents the performance after replacement. **b**, The average PCC for 26 test cell lines/tissues after replacing different numbers (10, 50, 200, 500) of TF profiles with the average value of the reference profile. **c**, The average PCC for 26 test cell lines/tissues after replacing different numbers (10, 50, 200, 500) of TF profiles with the zeros.

## Fig. S24

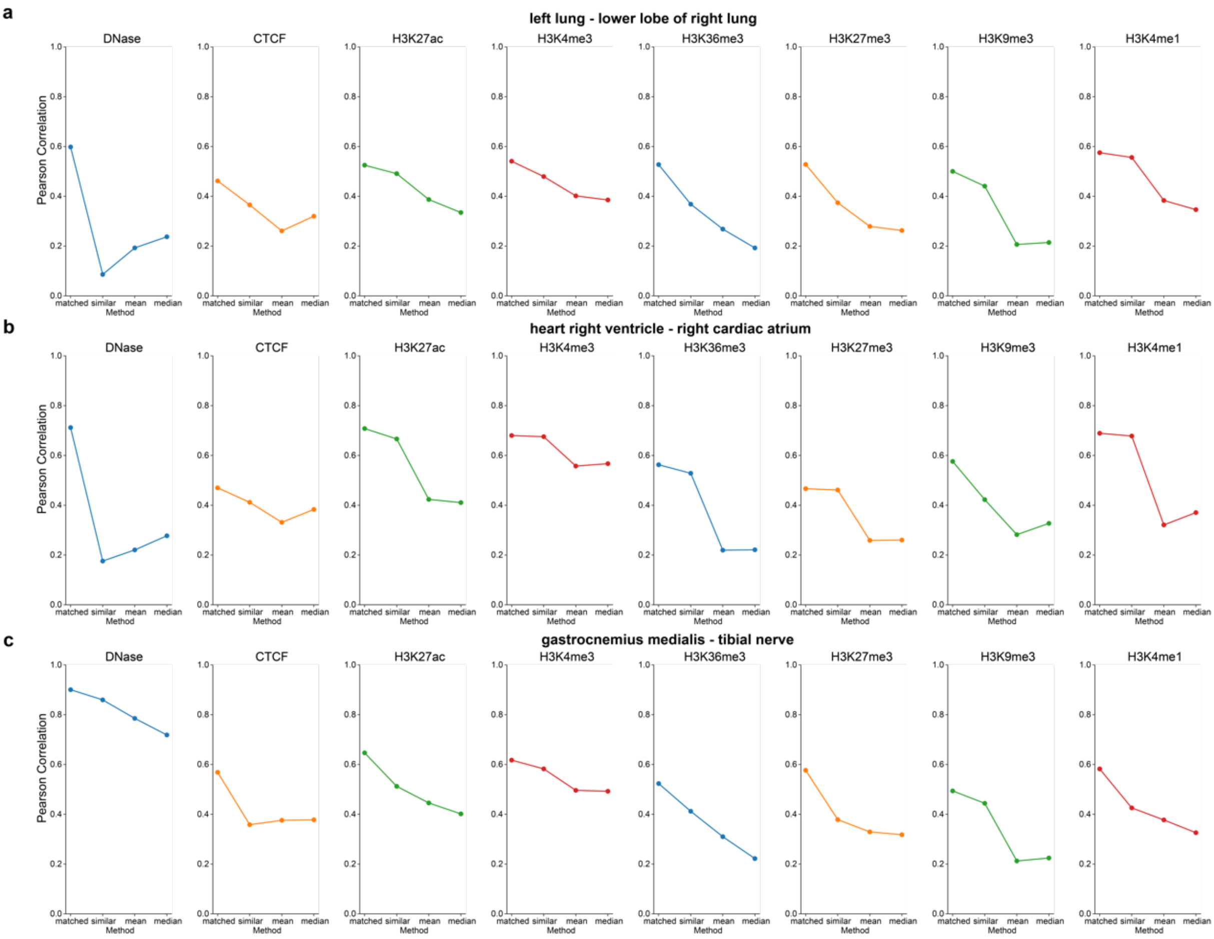


**Fig. S24. In three sets of similar cell types, the changes in predictive performance of eight types of epigenomic signals were simulated under different replacement strategies (similar, mean, median) when TF profiles were missing. a**, Eight-line charts show the predictive PCC for eight different epigenomic signals within the pair of similar cell types, the left lung and lower lobe of the right lung. The charts compare the PCC for predictions using the original TF profile (matched), the TF profile replaced with a similar cell type's profile (similar), and the TF profiles replaced with the reference profile's mean and median values. **b**, The change of predictive PCC for eight different epigenomic signals within the pair of similar cell types, the heart right ventricle and right cardiac atrium. **c**, The change of predictive PCC for eight different epigenomic signals within the pair of similar cell types, the gastrocnemius medialis and tibial nerve.

## Fig. S25

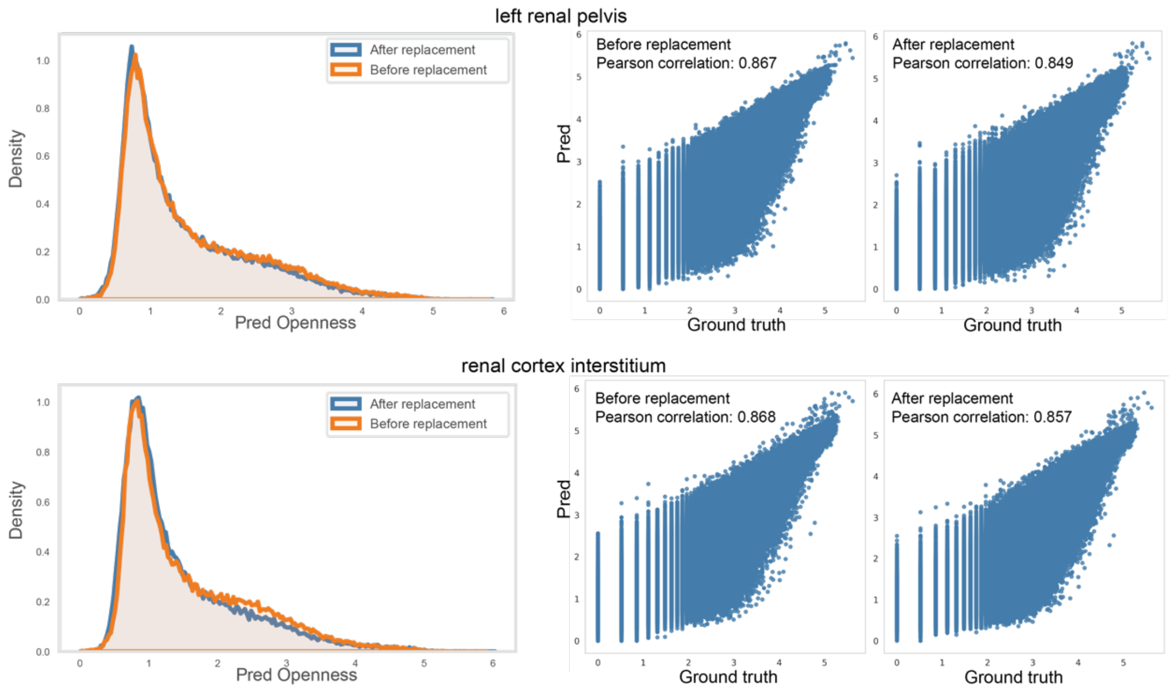


**Fig. S25. In the scenario of two TF profiles from the same cell type but different samples, the changes in DNase-seq predictive performance are simulated when missing TF profiles are replaced with the TF profile from a different sample. a**, The density plot (left) illustrates the predicted values before and after replacing the TF profile of the left renal pelvis from experiment ENCSR029FTY with that from experiment ENCSR410DUZ, and the scatter plot (right) shows the predicted values from both TF profiles against the ground truth, with the x-axis representing the true values and the y-axis representing the predicted values. **b**, The density plot (left) illustrates the predicted values before and after replacing the TF profile of the renal cortex interstitum from experiment ENCSR899SWV with that from experiment ENCSR436ZKE, and the scatter plot (right) shows the predicted values from both TF profiles against the ground truth, with the x-axis representing the true values and the y-axis representing the predicted values.

# Fig. S26

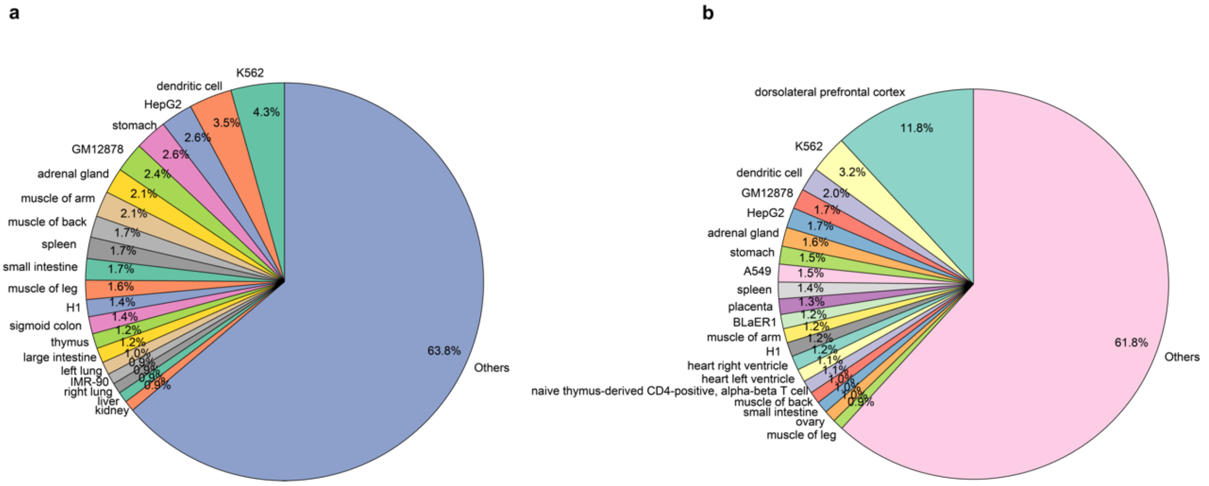


**Fig. S26. The pie charts illustrate the distribution of RNA-seq data from the ENCODE project. a**, the proportion of experiments for the top 15 cell lines/tissues in the hg19 (GRCh37) reference genome and **b**, the proportion of experiments for the top 15 cell lines/tissues in the hg38 (GRCh38) reference genome.

# Fig. S27

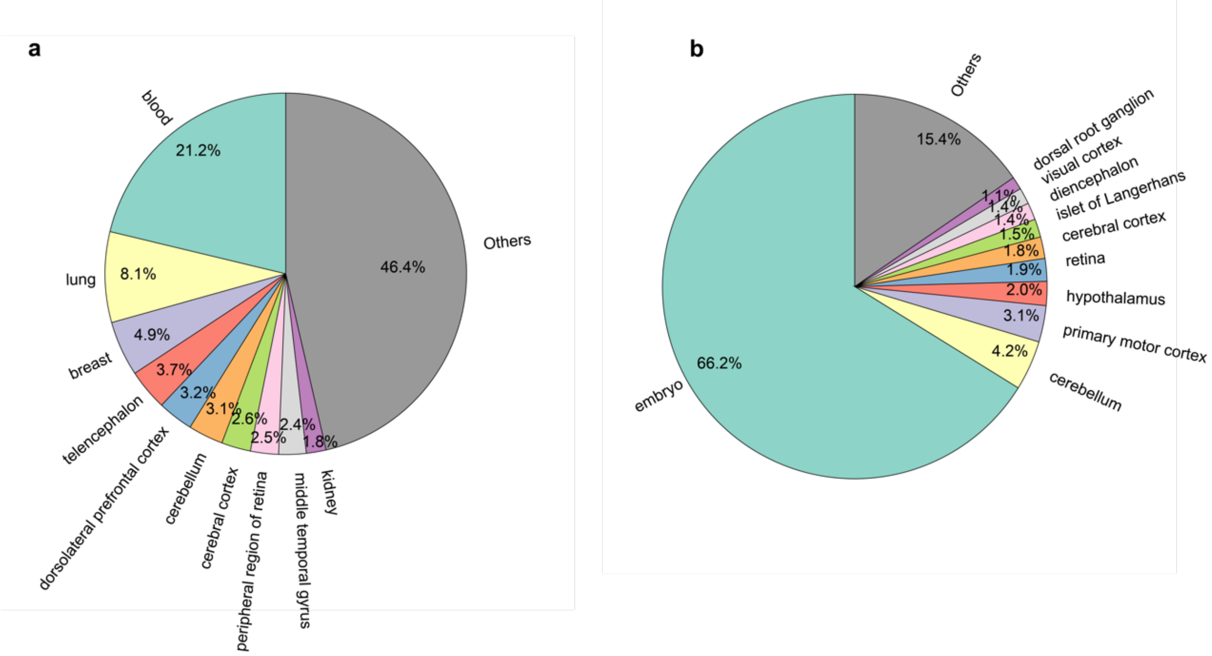


**Fig. S27. The pie charts illustrate the distribution of human and mouse single-cell RNA-seq data across various tissues from the CELLxGene database. a**, The top 10 tissues with the highest number of human cells and their respective proportions of total cell count. **b**, The top 10 tissues with the highest number of mouse cells and their respective proportions of total cell count.

## Fig. S28

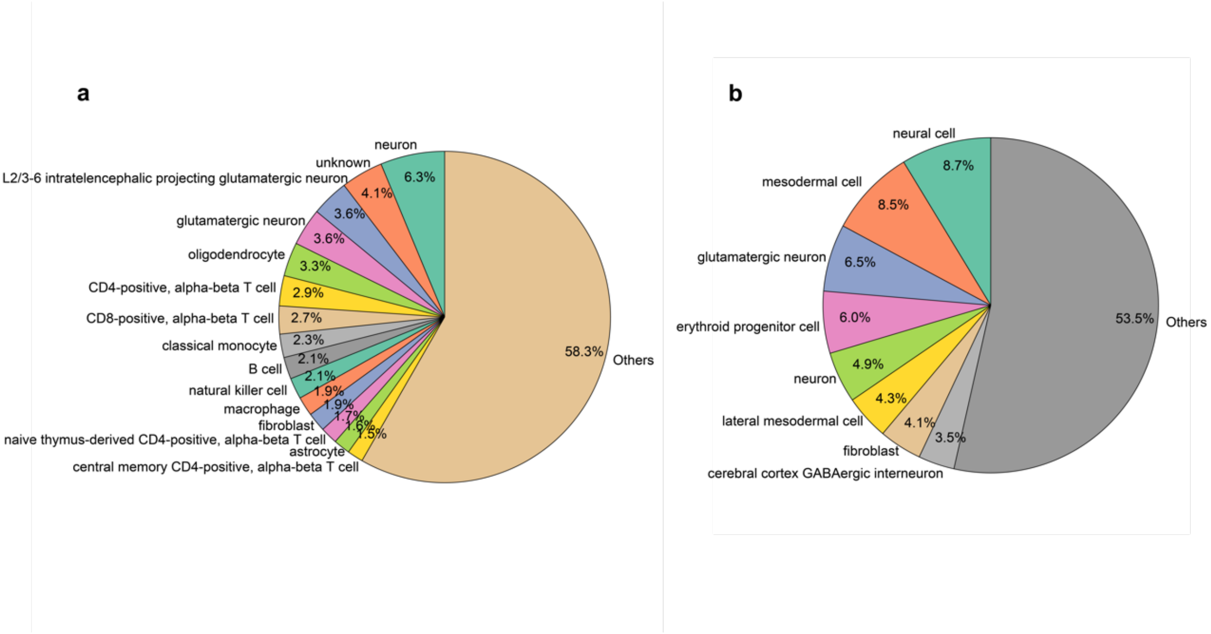


**Fig. S28. The pie charts illustrate the distribution of human and mouse single-cell RNA-seq data across various cell types from the CELLxGene database. a**, The top 15 cell types with the highest number of human cells and their respective proportions of total cell count. **b**, The top 8 cell types with the highest number of mouse cells and their respective proportions of total cell count.

## Fig. S29

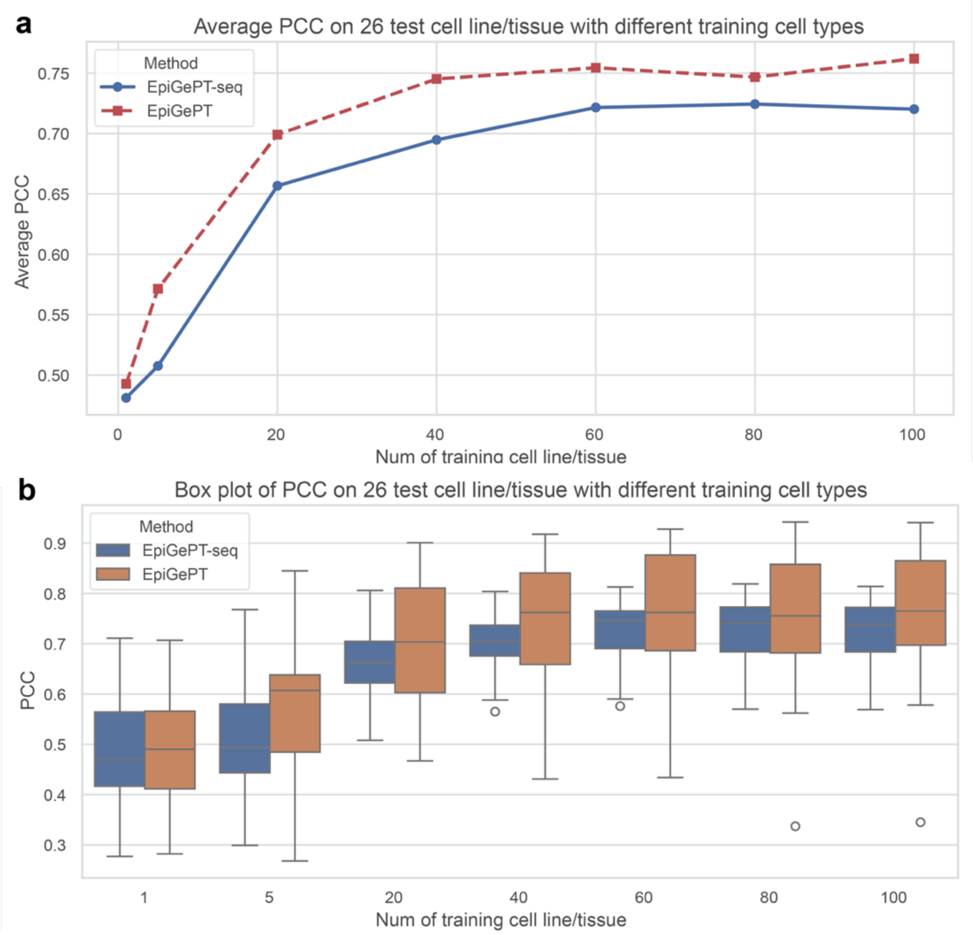


**Fig. S29. The Pearson correlation coefficient (PCC) for predicting chromatin accessibility (DNase-seq) under different numbers of training cell lines/tissues. a**, Line plot showing the average PCC across 26 test cell types, as a function of the number of training cell types. **b**, Box plot showing the distribution of PCC across 26 test cell types for different numbers of training cell types.

## Fig. S30

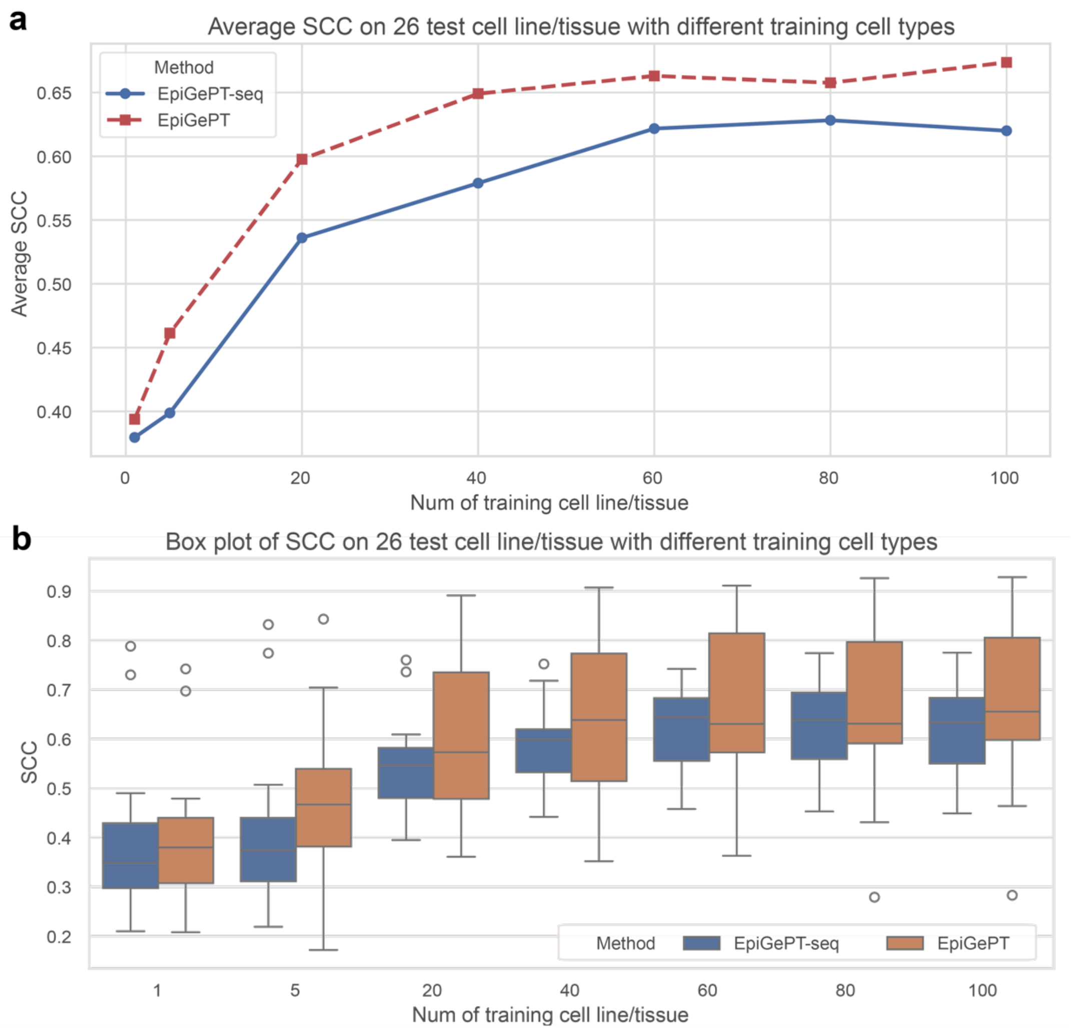


**Fig. S30. The Spearman correlation coefficient (SCC) for predicting chromatin accessibility (DNase-seq) under different numbers of training cell lines/tissues. a**, Line plot showing the average SCC across 26 test cell types, as a function of the number of training cell types. **b**, Box plot showing the distribution of SCC across 26 test cell types for different numbers of training cell types.

## Fig. S31

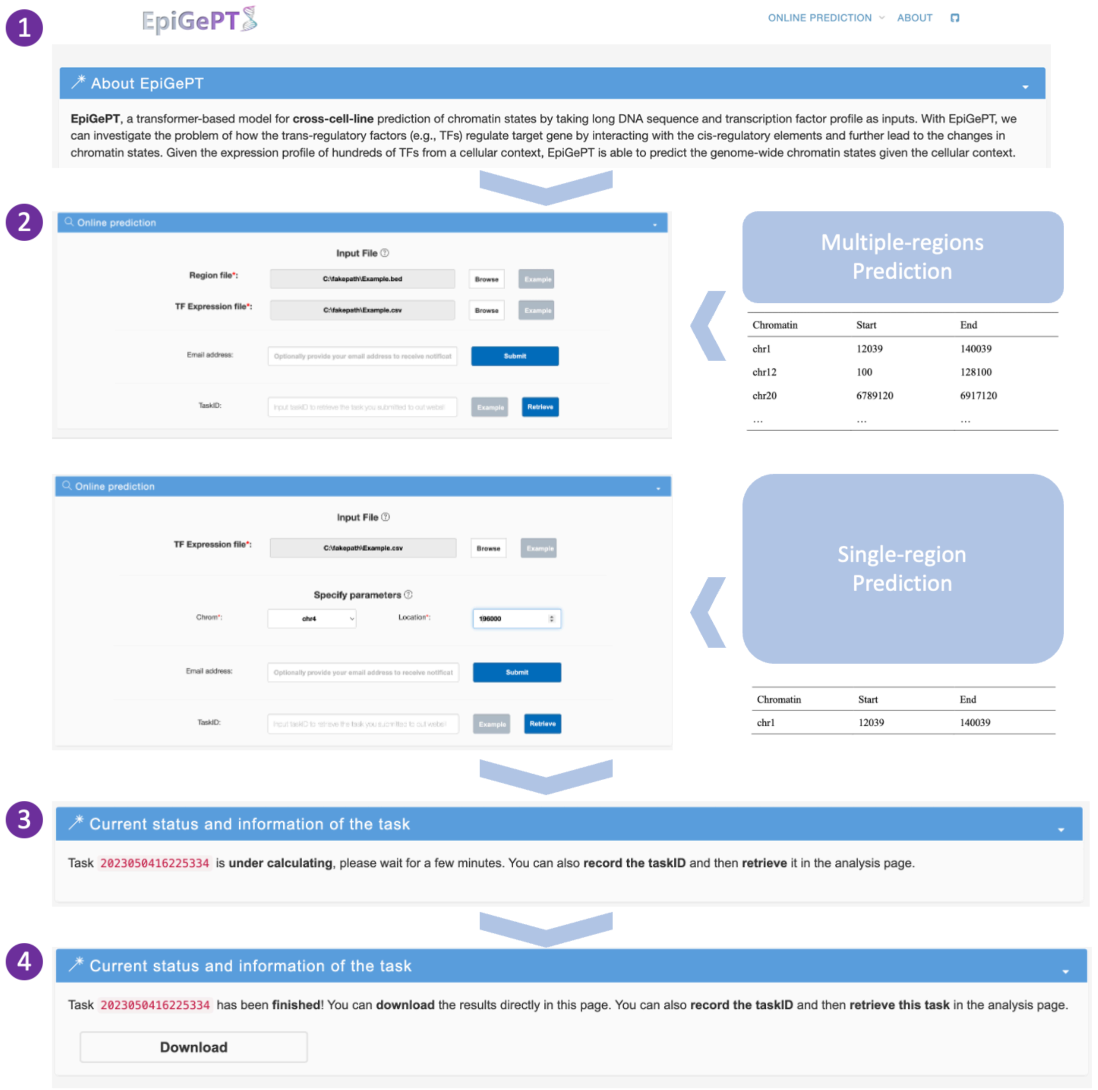


**Fig. S31. Case application of the EpiGePT-online.** Users can choose either single locus annotation or multi-region annotation on EpiGePT-online, and each genomic region requires a length of 128kbp. Users need to upload the TPM values of transcription factors expression simultaneously. After annotation, users can enter the result page and download the predicted files. The predictions are provided at the resolution of 128bp genomic bins, and users can obtain the predicted signals for these eight epigenomic profiles. Additionally, users have the option to download the prediction results in CSV format for further analysis and exploration.

## Fig. S32

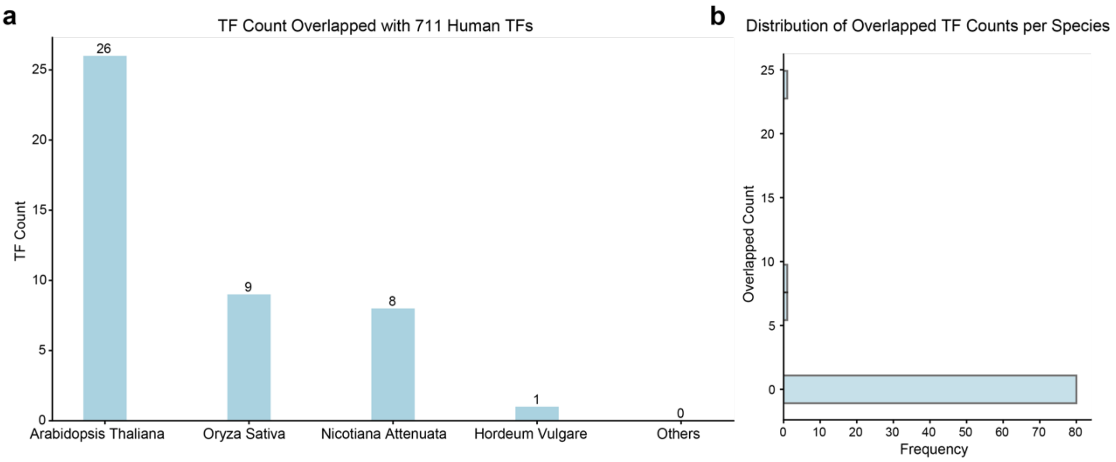


**Fig. S32. The overlap between human and plant gene annotations from the Ensembl Plant project in terms of the 711 selected TFs. a**, The bar plot illustrates the four species (Arabidopsis thaliana, Oryza sativa, Nicotiana attenuata, Hordeum vulgare) that have overlapping TFs with the 711 selected TFs, along with the number of overlapping TFs for each species. **b**, The histogram shows the distribution of the number of overlaps with the 711 selected human TFs across 83 plant species.

## Fig. S33

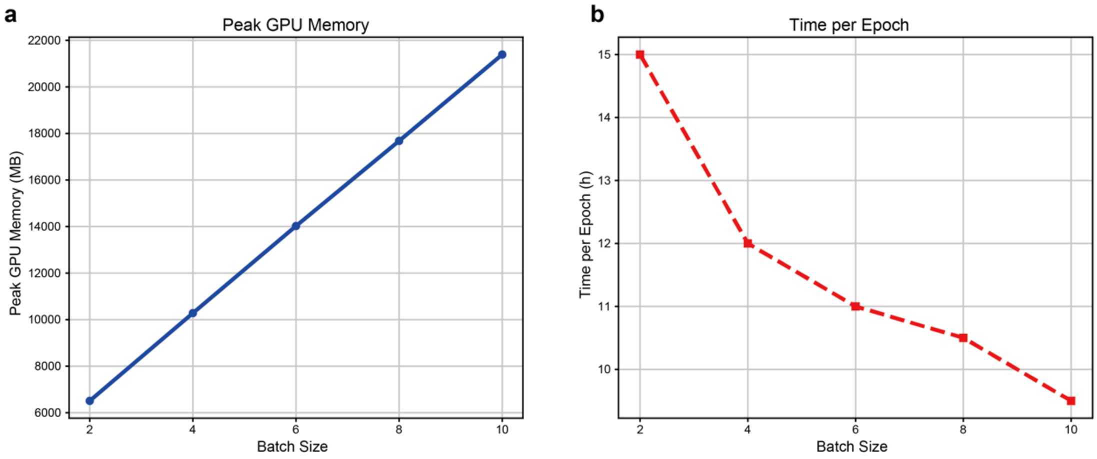


**Fig. S33. The computational resource usage, including peak GPU memory and time for training an epoch on an NVIDIA GeForce RTX 4090. a**, Line plot showing the GPU memory usage (in MB) as a function of batch size. **b**, Line plot showing the time taken to train one epoch (in hours) as a function of batch size.

# References


1. Song L, Crawford GE. DNase-seq: a high-resolution technique for mapping active gene regulatory elements across the genome from mammalian cells. Cold Spring Harbor Protocols. 2010;2010(2):pdb. prot5384.
2. Ernst J, Kellis M. Chromatin-state discovery and genome annotation with ChromHMM. Nature protocols. 2017;12(12):2478-92.
3. Bernstein BE, Stamatoyannopoulos JA, Costello JF, Ren B, Milosavljevic A, Meissner A, et al. The NIH roadmap epigenomics mapping consortium. Nature biotechnology. 2010;28(10):1045-8.
4. Avsec Ž, Agarwal V, Visentin D, Ledsam JR, Grabska-Barwinska A, Taylor KR, et al. Effective gene expression prediction from sequence by integrating long-range interactions. Nature methods. 2021;18(10):1196-203.
5. Consortium EP. An integrated encyclopedia of DNA elements in the human genome. Nature. 2012;489(7414):57.
6. d Galbraith E, sc Merleau N, mcdonald Smith B. The human cell count and size distribution. Proceedings of the National Academy of Sciences. 2023;120(39):e2303077120.
7. Biology CS-C, Abdulla S, Aevermann B, Assis P, Badajoz S, Bell SM, et al. CZ CELLxGENE Discover: A single-cell data platform for scalable exploration, analysis and modeling of aggregated data. BioRxiv. 2023:2023.10. 30.563174.
8. Heinz S, Benner C, Spann N, Bertolino E, Lin YC, Laslo P, et al. Simple combinations of lineage-determining transcription factors prime cis-regulatory elements required for macrophage and B cell identities. Molecular cell. 2010;38(4):576-89.
9. Yates AD, Allen J, Amode RM, Azov AG, Barba M, Becerra A, et al. Ensembl Genomes 2022: an expanding genome resource for non-vertebrates. Nucleic acids research. 2022;50(D1):D996-D1003.
10. Alvarez-Jarreta J, Amos B, Aurrecoechea C, Bah S, Barba M, Barreto A, et al. VEuPathDB: the eukaryotic pathogen, vector and host bioinformatics resource center in 2023. Nucleic acids research. 2024;52(D1):D808-D16.
11. Basenko EY, Pulman JA, Shanmugasundram A, Harb OS, Crouch K, Starns D, et al. FungiDB: an integrated bioinformatic resource for fungi and oomycetes. Journal of Fungi. 2018;4(1):39.
12. Breschi A, Gingeras TR, Guigó R. Comparative transcriptomics in human and mouse. Nature Reviews Genetics. 2017;18(7):425-40.
13. Nair S, Kim DS, Perricone J, Kundaje A. Integrating regulatory DNA sequence and gene expression to predict genome-wide chromatin accessibility across cellular contexts. Bioinformatics. 2019;35(14):i108-i16.
14. Liu Q, Hua K, Zhang X, Wong WH, Jiang R. DeepCAGE: incorporating transcription factors in genome-wide prediction of chromatin accessibility. Genomics, Proteomics & Bioinformatics. 2022;20(3):496-507.
15. Gasperini M, Hill AJ, McFaline-Figueroa JL, Martin B, Kim S, Zhang MD, et al. A genome-wide framework for mapping gene regulation via cellular genetic screens. Cell. 2019;176(1-2):377-90. e19.
16. Fulco CP, Nasser J, Jones TR, Munson G, Bergman DT, Subramanian V, et al. Activity-by-contact model of enhancer–promoter regulation from thousands of CRISPR perturbations. Nature genetics. 2019;51(12):1664-9.

17. Zeng W, Chen S, Cui X, Chen X, Gao Z, Jiang R. SilencerDB: a comprehensive database of silencers. Nucleic acids research. 2021;49(D1):D221-D8.
18. Zeng W, Liu Q, Yin Q, Jiang R, Wong WH. HiChIPdb: a comprehensive database of HiChIP regulatory interactions. Nucleic acids research. 2023;51(D1):D159-D66.
