# Supplementary material for: EpiGePT: a pretrained transformer-based language model for context-specific human epigenomics
Source: Genome Biol. 2024 Dec 18;25:310. doi: 10.1186/s13059-024-03449-7 (PMC11657395; doi:10.1186/s13059-024-03449-7)
Supplement: Supplementary file 3 — Additional file 3: Review history. [file 13059_2024_3449_MOESM3_ESM.docx]

Review History

**First round of review**

**Reviewer 1**

**Were you able to assess all statistics in the manuscript, including the appropriateness of statistical tests used?**

Yes, and I have assessed the statistics in my report.

**Comments to author:**

This study by Gao et al., entitled "EpiGePT: a Pretrained Transformer Model for Epigenomics" presents a machine learning transformer-based model designed to predict context-specific epigenomic signals by integrating chromatin interactions in their model training process. Implemented as software named EpiGePT (which I personally find to be a brilliant name) addresses limitations in existing genomic language models by incorporating transcription factor (TF) activities and 3D genome interactions. EpiGePT shows superior performance in predicting epigenomic signals and chromatin interactions across various cell types, offering insights into gene regulation and the impact of genetic variants. It also presents an online web application version of their software. Together, their study aims to support research into detecting gene regulatory mechanisms through epigenetic modifications, a key challenge in epigenomics research.

The manuscript is well written, easy to follow, and sufficiently structured, but the overall prediction improvements presented compared to previous approaches is rather less impressive and some benchmarking datasets used as ground truth could be challenged for their merits.

If the authors can further improve the model and address the major concerns raised below, I can support an editorial decision to accept this manuscript for publication (after major revision).

Major concerns:

1) The manuscript and title suggest that this procedure is easily applicable for any type of genomic and TF dataset, but fails to mention that training was performed on human data and tissue samples. I strongly suggest to add to the title and discuss in the rest of the manuscript that their model is currently designed for human genomes and cannot be transferred straight-forward to other species (maybe mouse, but not otherwise).
2) The manuscript also fails to mention how much resources were used to train their model and how genomics communities around other species with much less data resolution than human (e.g. ENCODE project etc) can expect to find prediction improvements (since even with human data their model prediction improvements are rather small).
3) A clear path forward and detailed description should be added on how users can retrain their model with their species specific datasets and to what degree the model trained on human samples can be transferred to other species such as plants, fungi, etc.
4) Some statements such as "In contrast, each combination of input region and cellular context provides an example for training in EpiGePT, thus providing a much larger number of examples available for model training" need to be clarified in detail. Here it is not clear to me how users who don't have access to TF or tissue data can benefit from their approach.
5) Please also specify how many different tissue samples need to be provided to their model (minimum number of different tissues) to achieve superior prediction accuracy compared to existing methods. In the presented case 129 cellular contexts were processed from the ENCODE project. Are these 129 cellular contexts the minimum number to achieve improvements? If yes, how can users from other model species achieve this high data resolution or will the model also be useful with less cellular context samples? If yes, how?
6) In lines 130 - 132, the authors write: Finally, we compared EpiGePT and ChromDragoNN [6] in the binary classification of functional regions versus nonfunctional regions, using the functional chromatin status derived from ChromHMM [20] annotations as ground truth (Supplementary Text S6). While ChromHMM is a well established software tool for human data, it would be important to see how the different models (ChromDragoNN, EpiGePT, ChromHMM) benchmark when each model serves as ground truth. E.g. how does EpiGePT and ChromHMM benchmark when ChromDragoNN is used as ground truth, or how does ChromDragoNN and ChromHMM perform when EpiGePT is used as ground truth etc. Please provide a comparison matrix where all combinations are tested.
7) In lines 140 - 142 the authors write: EpiGePT demonstrates a 3.3% to 5.2% higher performance than Enformer in terms of the median Pearson correlation coefficient under the three prediction settings (Fig. 2b). While this improvement is rather incremental and not substantial, to make any outperformance claim, the Enformer model would have to be retrained using the same training dataset used for EpiGePT training. It was not clear to me whether or not this has been done?

8) In contexts where TF profiles or chromatin interaction data are sparse or unavailable the authors do not discuss how their model's predictive accuracy may change or even suffer. Please discuss this point in detail.
9) Training and running predictions with EpiGePT may require substantial computational resources, which may limit its accessibility to researchers with limited access to high-performance computing facilities. It would be paramount that the authors provide more details on the time computational complexities required to train and employ their models.
10) Given the complexity of the model and the extensive training on specific tissue datasets, there is a potential risk of overfitting to the training data, which may affect the model's performance on truly novel data. How do the authors avoid overfitting?

**Reviewer 2**

**Were you able to assess all statistics in the manuscript, including the appropriateness of statistical tests used?**

Yes, and I have assessed the statistics in my report.

**Comments to author:**

Gao et. al. presented EpiGePT, a deep learning model using DNA sequence and epigenetic profiles (TF binding and chromatin interactions) to pre-train and perform multiple downstream tasks including the prediction of epigenetic tracks, chromatin interactions, TF binding, variant effect prediction and so on. For each of these downstream analyses, the authors presented a comprehensive performance assessment with reference datasets and state-of-the-art methods. The manuscript is well written and the methodology and results are well described. I believe the methods, results and associated source code and webserver can be valuable to the community. I'd recommend accepting the manuscript but request the authors to address the mentioned comments for a thorough performance analysis and also recommend the most useful model for future use.

Comments:
1. The current manuscript proposes multiple models and settings: EpiGePT, EpiGePT(seq) excluding the TF based learning, EpiGePT(3D: setting B) which incorporates 3D interactions, EpiGePT(3D: setting A) which uses self-attention scores for predicting E-P interactions, and EpiGePT(3D: finetuned) which basically fine-tunes EpiGePT for predicting 3D interactions. Different settings have different utility. However, for simplicity and future use by the community, the authors should propose one particular setting of EpiGePT for predicting epigenomic tracks and chromatin interactions, and present its performance in all of the mentioned objectives.
2. As mentioned in Suppl. Text S5, the authors modified Enformer by: 1) reducing the number of layers to 3, 2) Enformer+: using reference TF binding profiles. Can the authors present a performance comparison between EpiGePT, Enformer original (i.e., pre-trained Enformer model from Avsec et al. 2021), Enformer with 3 encoder layers but without using reference TF binding profiles, and Enformer+ which uses 3 encoder layers and reference TF binding profiles. I understand that original Enformer model does not satisfy cross-validation principle but this benchmarking would provide a upper bound of the performance metrics. Again, the authors need to choose one version of EpiGePT model (I would prefer EpiGePT-3D using reference TF information and chromatin interactions).

Minor comments
1. Fig. S3-e: Authors should indicate different track types for individual correlation plots.
2. When referring supplementary figures, such as Fig. S4, please refer to the exact figure i.e., Fig. S4(b).
3. Fig. 3h - EpiGePT should be EpiGePT(finetuned). The performance values and scales are not clear. Performance should be compared with EpiGePT-3D model.

**Authors Response**

**Point-by-point responses to the reviewers’ comments:**

**Reviewer #1:**

**This study by Gao et al., entitled "EpiGePT: a Pretrained Transformer Model for Epigenomics" presents a machine learning transformer-based model designed to predict context-specific epigenomic signals by integrating chromatin interactions in their model training process. Implemented as software named EpiGePT (which I personally find to be a brilliant name) addresses limitations in existing genomic language models by incorporating transcription factor (TF) activities and 3D genome interactions. EpiGePT shows superior performance in predicting epigenomic signals and chromatin interactions across various cell types, offering insights into gene regulation and the impact of genetic variants. It also presents an online web application version of their software. Together, their study aims to support research into detecting gene regulatory mechanisms through epigenetic modifications, a key challenge in epigenomics research.**

**The manuscript is well written, easy to follow, and sufficiently structured, but the overall prediction improvements presented compared to previous approaches is rather less impressive and some benchmarking datasets used as ground truth could be challenged for their merits*.***

**If the authors can further improve the model and address the major concerns raised below, I can support an editorial decision to accept this manuscript for publication (after major revision).**

## *RESPONSE*

*We appreciate the reviewer for the positive feedback of our work, as well as the enthusiastic assessment and constructive comments, which have helped us improve the manuscript. In response to the comments, we have made detailed revisions to the manuscript, including extensive additional experiments based on the constructive feedback provided. We have also incorporated these new experiments and results into the revised manuscript. Please find our point-to-point response to each comment below.*

# MAJOR SPECIFIC COMMENT 1

**1) The manuscript and title suggest that this procedure is easily applicable for any type of genomic and TF dataset, but fails to mention that training was performed on human data and tissue samples. I strongly suggest to add to the title and discuss in the rest of the manuscript that their model is currently designed for human genomes and cannot be transferred straightforward to other species (maybe mouse, but not otherwise).**

# *RESPONSE TO MAJOR SPECIFIC COMMENT 1*

*We appreciate the reviewer for this constructive comment. We agree that the potential applicability of EpiGePT to other species is extremely important and should be systematically discussed. To clarify the main focus on human species of our paper, we changed the paper title to “EpiGePT: a Pretrained Transformer Model for Human Epigenomics”. More importantly, we have conducted extensive experiments based on two different settings: zero-shot prediction and model fine-tuning on epigenomic profiles of mouse, to demonstrate the predictive performance of EpiGePT when applied to cross-species data.*

***Before the experiments, we first provide a detailed explanation regarding the crossspecies modeling capability of EpiGePT, along with its limitations****. First and foremost, we emphasize that EpiGePT is primarily trained on human data, but it has the potential for crossspecies modeling provided certain requirements are met. Overall, the data requirements for applying EpiGePT to other species involve* ***two key aspects****: (i) a high-quality reference genome and (ii) a high degree of conservation in transcription factors (TFs) compared to humans. Specifically, EpiGePT primarily consists of two input modules: one for DNA sequences and another for cell type-specific transcription factors (TFs). For the former, a complete and high-quality reference genome is essential. For instance, EpiGePT cannot model species such as complex fungi that lack a comprehensive reference genome. Regarding the latter, our TF module models the binding interactions between TFs and DNA sequences and captures the expression patterns of these key TFs across different cell types. These TFs and motifs are pre-selected based on human biological knowledge, necessitating a high degree of structural and functional conservation between the TFs in other species and those in humans. For example, the low conservation of TFs between plants, most unicellular organisms and humans leads to significant variability in the TF module, leading to ineffective modeling with EpiGePT for these species. However, species that satisfy both conditions, such as mouse and primates, are well-suited for epigenomic modeling with EpiGePT.*

*Next, to address whether EpiGePT can transfer the knowledge learned on human to other species, we take mouse data as an example to illustrate the cross-species predictive capability of EpiGePT through two different settings:* ***zero-shot learning*** *and* ***model fine-tuning****.*

*The two key aspects we identified are both satisfied by mouse data. First, this species has a high-quality reference genome, and we used the GRCm38 (mm10) version to provide region information. Second, the TF binding profiles for human and mouse TFs are nearly identical. We identified 688 corresponding TFs in mouse RNA-seq data using the gene annotation file (vM25) from the GENCODE project (https://www.gencodegenes.org) from the 711 selected TFs of humans, while the expression values of the remaining transcription factors were set to zero.*

***In terms of data collection and processing****, the ENCODE project (Consortium, 2012) includes a substantial amount of bulk-level epigenomic and gene expression experimental data for mouse. We took the DNase-seq and RNA-seq data from ENCODE as illustrative examples. Specifically, we collected the bulk data from three organ-level datasets for prediction: brain (ENCSR000COE), lung (ENCSR000CNM), and kidney (ENCSR000CNG). For DNase-seq data, we annotated the chromatin openness in genomic regions using OpenAnnotate (Chen, et al., 2021). The foreground openness in these three organs was annotated on the regions based on the corresponding narrow peak files (ENCFF941CCB, ENCFF268DLZ and*

*ENCFF587XGH), from which genomic regions were selected for prediction. Then, each peak was extended to a fixed length centered on the original peak center. After processing, we obtained 247,750 peaks for brain data, 243,900 peaks for lung data, and 180,400 peaks for kidney data. For RNA-seq data, we used RNA-seq data from the same samples (ENCFF455OZW, ENCFF641SMI and ENCFF876OPZ), with TPM values representing the expression levels of corresponding transcription factors. For TF binding status, due to the high conservation of TF binding between humans and mouse, we utilized the same homer tool (Heinz, et al., 2010) and motif database (Kulakovskiy, et al., 2018) to scan for TF binding status on peaks. At this point, we have completed the preprocessing of the data required for model training and prediction. Next, to evaluate the cross-species prediction capability of EpiGePT, we conducted both zero-shot prediction and model fine-tuning using mouse data.*

***For the zero-shot experiments****, we directly utilized the pre-trained EpiGePT model, which was pre-trained on 100 human cellular contexts and 1.17 million genomic bins, and directly applied it to predict DNase-seq signals of all peaks on mouse data to observe the discrepancies between predicted chromatin accessibility and the ground truth. As shown in* ***Fig. R1b****, we conducted predictions on the corresponding peaks of three cell types (brain, lung and kidney). Notably, even without fine-tuning the model, EpiGePT demonstrated a substantial predictive accuracy by achieving the Pearson correlation coefficient (PCC) of 0.330 to 0.405. For instance, the PCC in the brain data reached 0.405, while the Spearman correlation coefficient (SCC) was 0.347. Furthermore, to enable a more comprehensive comparison with the fine-tuned model, we performed predictions only on the peaks located on chromosomes 18-19 as well as the X and Y chromosomes. The results demonstrated a satisfactory level of predictive performance on the three different cell types with PCC ranging from 0.344 to 0.422 (****Fig. R1a****).*

***For the model fine-tuning experiments****, we first selected the peaks on chromosomes 1-17 from the three cell types as the training set and subsequently fine-tuned the model separately on the data from each cell type. We then performed cross-region predictions on the remaining chromosomes (chromosomes 18,19, X and Y) for each cell type. The results indicate that after fine-tuning with a limited amount of data, the model demonstrated much stronger predictive performance than zero-shot prediction. For instance, the Pearson correlation coefficient (PCC) for predictions on the test chromosomes in the brain reached 0.603 (****Fig. R2a****). This finding underscores the ability of EpiGePT to make effective predictions on new species with an effective fine-tuning strategy. Additionally, compared to the zero-shot prediction, the predictive accuracy improved significantly across all three cell types following fine-tuning even with a small dataset, with PCC increases of* ***18.1%****,* ***18.4%****, and* ***19.5%*** *observed in the brain, kidney, and lung, respectively (****Fig. 2c****). Furthermore, to further elucidate the ability of EpiGePT to transfer to other species, we tested EpiGePT on a more challenging task by predicting on novel cell types and genomic regions in another species. We trained separate fine-tuned models for the brain, kidney, and lung, and conducted predictions on the test regions for each cell type. As shown in* ***Fig. R2b****, taking the brain as an example, the model trained on lung data achieved a PCC of 0.490 when predicting the brain test data. Similarly, the model trained on kidney data yielded a PCC of 0.448 for the brain test data. Although these values were lower than the PCC of 0.603 achieved by the model trained specifically on brain data, they still exceeded the performance of the zero-shot learning. This demonstrates that after pre-training on human data, EpiGePT can significantly enhance predictive performance even with a minimal amount of mouse data for fine-tuning, thus validating the capability of EpiGePT to transfer to other species, such as mouse.*

*In conclusion, we have validated the applicability of EpiGePT to other species through both zero-shot learning and model fine-tuning approaches on mouse data. Additionally, we have discussed two key conditions that enable this application: DNA sequence input and TF input. This discussion has been included in the Discussion section in the revised manuscript. Besides, we modified the title to reflect its current focus on human data and incorporated the crossspecies prediction into the revised manuscript as a small section to reflect the ability of EpiGePT transferred to other species. We sincerely thank the reviewer again for this constructive comment, which has helped improve the clarity and completeness of our manuscript.*

***Fig. R1. Zero-shot learning of EpiGePT on chromatin accessibility (DNase-seq) prediction on mouse data.*** *(a) Scatter plots depicting the predicted openness and ground truth (logarithmically transformed foreground read count) for peaks on chromosomes 18-19 as well as the X and Y chromosomes for the brain (22,750 regions), kidney (16,250 regions), and lung (22,450 regions). (b) Scatter plots depicting the predicted openness and ground truth (logarithmically transformed foreground read count) for peaks on all chromosomes for the brain (247,750 regions), kidney (180,400 regions), and lung (243,900 regions).*

***Fig. R2. Model fine-tuning EpiGePT on chromatin accessibility (DNase-seq) prediction on mouse data.*** *(a) Scatter plots depicting the predicted openness and ground truth (logarithmically transformed foreground read count) using fine-tuned model for peaks on chromosomes 18-19 as well as the X and Y chromosomes for the brain, kidney, and lung tissues. (b) The heatmap matrix represents the performance across different cell types, with columns indicating the predicted cell types and rows indicating the training cell types. (c) The comparison of performance (PCC) between finetuned and zero-shot models for predicting peaks on chromosomes 18-19 and the X and Y chromosomes across brain, kidney, and lung cell types shows the performance differences after finetuning.*

## MAJOR SPECIFIC COMMENT 2

**2) The manuscript also fails to mention how much resources were used to train their model and how genomics communities around other species with much less data resolution than human (e.g. ENCODE project etc) can expect to find prediction improvements (since even with human data their model prediction improvements are rather small).**

## *RESPONSE TO MAJOR SPECIFIC COMMENT 2*

*We appreciate the reviewer for this thoughtful comment. The reviewer raises two core issues: 1) the resources utilized to train the model, and 2) how the inclusion of other species with much lower data resolution could lead to performance improvements. We apologize for not clearly addressing these two points in the original manuscript. Below, we provide detailed explanations to the reviewer on both matters.*

1. ***Resources Used to Train the Model***

***Data Resources****. We have described all the data used in the manuscript and provided details of the public bulk sequencing experimental data we used in the supplementary tables. Specifically, we trained models on two human reference genomes (GRCh37 and GRCh38). For the hg19 model, we compiled data from 28 cell lines/tissues and 8 epigenomic signals across 13,300,000 genomic bins, which included 224 DNase-seq and ChIP-seq experiments from the ENCODE project. Additionally, we utilized data from 303 RNA-seq experiments. For the hg38 model, we assembled data from 104 cell lines/tissues and 8 epigenomic signals across 15,870,000 genomic bins, comprising 625 DNase-seq and ChIP-seq experiments, along with 1,309 RNAseq experiments from the ENCODE project. Additionally, we collected chromatin interaction data from 13 cell types via the HiChIPdb database (Zeng, et al., 2023), which were used to guide the learning process for the self-attention module during model training. This allowed the model to learn underlying gene regulatory relationships.*

***Computational Resources****. The total parameter size of EpiGePT is 71.3M, making it feasible to train and make inference on a single GPU. We also tracked the performance across three hardware configurations (NVIDIA GeForce RTX 3090, RTX 4090, and NVIDIA RTX A6000), each using a single GPU (****Table R1-R3****,* ***Fig. R3****). For example, on a single NVIDIA RTX A6000 (48GB memory), peak GPU memory usage reached 31.8GB with a batch size of 16, and training one epoch took around 14 hours. The training dataset comprised 85 cell lines or tissues and 15,870 regions of 128kbp, totaling roughly 15 million bins. Training the full model on a single A6000 GPU would take approximately 2–3 weeks. However, this process can be significantly accelerated by utilizing multiple GPUs in parallel, employing either Data Parallel (DP) or Distributed Data Parallel (DDP) strategies. Regarding memory usage, a GPU with*

*48GB or 24GB of memory is sufficient, and the peak memory usage reached 341.5GB when loading all motif scores and labels for 104 cell lines/tissues into memory to enhance dataloader efficiency. To lower the requirement for memory usage during training, sample data can be stored on disk and loaded in batches, rather than loading all data into memory at once. This approach significantly reduces memory usage, making it feasible to train the model on most standard computing systems. We have also provided a detailed explanation of this in response to major comment 9, with the relevant tables and figures included at the end of that response.*

2. ***Applicability to Species with Lower Data Resolution***

***Data Resolution****. This term has multiple meanings. We define the data resolution as the length of the genomic bins, which is set to be 128bp. This genomic bin size does not directly affect the model’s performance since we use BAM files when processing epigenomic sequencing data, as long as BAM files are available, there are no inherent limitations on data resolution, even when applying the model to other species. The data resolution may also refer to the number of available cellular contexts/experiments in other species. We agree that the data resolution is much limited in other species, which raises concerns for EpiGePT modeling on other species.*

***Performance with Lower Data Quantity****. We conducted comprehensive experiments to investigate the relationship between data quantity in other species and performance improvement. In fact, compared to baseline methods, the improvements achieved by our model are not only dependent on the quantity of data, but also from the modeling approach design, which enables the model to maintain effectiveness even with limited data availability. Specifically, compared to purely DNA sequence-based models like Enformer (Avsec, et al., 2021), the improvements offered by EpiGePT come from the incorporation of cell-type-specific information and TF binding status through TF module. This inclusion of complex regulatory information across different cellular contexts can provide a more comprehensive and accurate interpretation of epigenomic signals. Compared to other convolutional neural network-based deep learning models like ChromDragoNN (Nair, et al., 2019) and DeepCAGE (Liu, et al., 2022), as well as regression-based machine learning models like BIRD (Zhou, et al., 2017), even though these models incorporate cell-type-specific inputs, their modeling approaches are typically designed for short DNA sequences, which restricts the model’s ability to capture longrange interactions between genomic regions, leading to a constrained perspective on genomic architecture. In contrast, the self-attention module in EpiGePT naturally addresses this limitation by enabling the model to consider long-range interactions across genomic regions. This leads to performance improvements when trained even on the same number of cellular contexts, and EpiGePT has demonstrated superior results in functional region classification tasks across 56 cellular contexts (****Fig. S5a-b****).*

*To more clearly explain the relationship between data quantity in other species and performance improvement, we provide the following three types of experimental results.*

***First****, when the data quantity is large, as demonstrated in the results section of the original manuscript, EpiGePT has already achieved significant improvements in certain tasks. For instance, in the classification task of functional chromatin regions, EpiGePT exceeded the best baseline method (ChromDragoNN) by* ***8.1%*** *in terms of auROC (Fig. S5a). In the binary classification task of predicting open chromatin regions, EpiGePT outperformed DeepCAGE by* ***14.3%*** *in auPR (Fig. 2c). Additionally, compared to pretrained Enformer, we achieved a* ***7.0%*** *higher average PCC across 78 genomic tracks. These metrics demonstrate that EpiGePT can deliver prediction improvements over baseline models with a sufficient amount of pretraining data and can exhibit substantial improvements in specific tasks.*

***Second****, when data quantity is limited, we simulate this scenario by downsampling human data across different cellular contexts. We then compare the performance and improvement of EpiGePT with the best baseline method for DNase-seq profile prediction, EpiGePT-seq, across different numbers of training cellular contexts. We reduced the number of training cellular context to 1, 5, 20, 40, 60, 80, and 100. As shown in* ***Fig. R4a****, even when the training data consisted of only one cellular context, EpiGePT still outperformed the second-best performing model, EpiGePT-seq, by an average PCC improvement of 1.4% across 26 test cellular contexts. Overall, as the number of training cellular contexts increased, the performance of both EpiGePT and EpiGePT-seq gradually improved and EpiGePT consistently demonstrated prediction improvements compared to models that used pure DNA sequences as input. Additionally, when the number of training cellular contexts reached 40, the performance of EpiGePT exceeded that of Enformer and ChromDragoNN (the best-performing convolution-based baseline methods), which were retrained on 103 cellular contexts. This comparison further demonstrates that the improvements in prediction are attributable to the additional biological knowledge provided by the TF module, rather than simply relying on the scale of the data. Furthermore, we also investigated the distribution of mouse data from the ENCODE project. Before any processing, there were totally 38 cell lines, tissues, and primary cells in mouse that provided both DNaseseq and total RNA-seq or polyA plus RNA-seq sequencing data simultaneously. Based on the results on human data, we can infer that EpiGePT can still achieve commendable results with limited cellular context data from other species such as mouse. We have also provided a detailed explanation of this in response to major comment 5, with the relevant tables and figures included at the end of that response.*

***Third****, we used three organ-level tissue DNase-seq experiments on mouse from ENCODE project as an example to explore the prediction improvements that EpiGePT can achieve with limited data on other species. Specifically, we fine-tuned both EpiGePT and EpiGePT-seq, which had been pre-trained on the same 100 human cellular contexts, using chromatin data from chromosomes 1-17 of mouse brain, lung, and kidney tissues. We then predicted chromatin accessibility on the remaining genomic regions. As shown in* ***Fig. R5a-b****, EpiGePT demonstrated significant improvements in various metrics such as PCC, SCC, Mean squared error (lower values indicate better performance), and R-squared. For example, the PCC on brain predictions improved by 10.0%. These results suggest that, even with fine-tuning on other species with limited data, EpiGePT can consistently outperform EpiGePT-seq (best performance baseline method when predicting DNase-seq).*

*In summary, we have provided a more detailed description of the data and computational resources in the revised manuscript, along with a thorough discussion on the relationship between prediction improvement and data volume. Moreover, as demonstrated in our response to major comment 1, the model has been successfully applied to mouse data, and it consistently outperformed pure sequence-based methods on this dataset, further validating its cross-species applicability. Thus, even in cases where data from other species is limited, EpiGePT is still capable of delivering performance improvements.*

***Table R1****. The computational resource usage, including peak GPU memory and peak RAM consumption (NVIDIA GeForce RTX 3090).*

| *Batch size* | *GPU Device* | *Time*  *(h)/epoch* | *Peak GPU*  *memory (MB)* | *Peak RAM*  *memory (GB)* |
| --- | --- | --- | --- | --- |
| *2* | *NVIDIA GeForce RTX 3090* | *19* | *6356* | *341.529* |
| *4* | *NVIDIA GeForce RTX 3090* | *15-16* | *10118* | *341.529* |
| *6* | *NVIDIA GeForce RTX 3090* | *13-14* | *13860* | *341.529* |
| *8* | *NVIDIA GeForce RTX 3090* | *13* | *17526* | *341.529* |
| *10* | *NVIDIA GeForce RTX 3090* | *13* | *21210* | *341.529* |
| *CPU info: Intel(R) Xeon(R) CPU E5-2680 v4 @ 2.40GHz* | | | |  |

***Table R2****. The computational resource usage, including peak GPU memory and peak RAM consumption (NVIDIA GeForce RTX 4090).*

| *Batch size* | *GPU Device* | *Time*  *(h)/epoch* | *Peak GPU*  *memory (MB)* | *Peak RAM*  *memory (GB)* |
| --- | --- | --- | --- | --- |
| *2* | *NVIDIA GeForce RTX 4090* | *15* | *6510* | *341.532* |
| *4* | *NVIDIA GeForce RTX 4090* | *12* | *10280* | *341.532* |
| *6* | *NVIDIA GeForce RTX 4090* | *11* | *14022* | *341.532* |
| *8* | *NVIDIA GeForce RTX 4090* | *10-11* | *17680* | *341.532* |
| *10* | *NVIDIA GeForce RTX 4090* | *9-10* | *21390* | *341.532* |
| *CPU info: Intel(R) Xeon(R) Gold 5220 CPU @ 2.20GHz* | | | |  |

***Table R3****. The computational resource usage, including peak GPU memory and peak RAM consumption (NVIDIA RTX A6000).*

| *Batch size* | *GPU Device* | *Time*  *(h)/epoch* | *Peak GPU*  *memory (MB)* | *Peak RAM*  *memory (GB)* |
| --- | --- | --- | --- | --- |
| *2* | *NVIDIA RTX A6000* | *19* | *6356* | *341.529* |
| *4* | *NVIDIA RTX A6000* | *16-17* | *10126* | *341.529* |
| *8* | *NVIDIA RTX A6000* | *14-15* | *17526* | *341.529* |
| *10* | *NVIDIA RTX A6000* | *14-15* | *21210* | *341.529* |
| *16* | *NVIDIA RTX A6000* | *14* | *31792* | *341.529* |
| *CPU info: AMD EPYC 7643 48-Core Processor* | | |  |  |

***Fig. R3.*** *The computational resource usage, including peak GPU memory and time for training an epoch on an NVIDIA GeForce RTX 4090. (a) Line plot showing the GPU memory usage (in MB) as a function of batch size. (b) Line plot showing the time taken to train one epoch (in hours) as a function of batch size.*

***Fig. R4. The Pearson correlation coefficient (PCC) for predicting chromatin accessibility (DNaseseq) under different numbers of training cell lines/tissues.*** *(a) Line plot showing the average PCC across 26 test cell types, as a function of the number of training cell types. (b) Box plot showing the distribution of PCC across 26 test cell types for different numbers of training cell types. (c) Bar plot with error bars showing the PCC across 26 test cell types for different numbers of training cell types.*

***Fig. R5. The cross-chromosome predictive performance of pretrained EpiGePT and EpiGePTseq after fine-tuning on mouse data.*** *(a) The bar plot illustrates the Pearson correlation coefficient (PCC), Spearman correlation coefficient (SCC), mean squared error (MSE), and R square between the predicted and true chromatin openness for EpiGePT and EpiGePT-seq, across 22,750 bins in the brain, 22,450 bins in the lung, and 16,200 bins in the kidney. (b) The scatter plots illustrate the relationship between the predicted and true chromatin openness for EpiGePT and EpiGePT-seq, across 22,750 bins in the brain, 22,450 bins in the lung, and 16,200 bins in the kidney.*

## MAJOR SPECIFIC COMMENT 3

**3) A clear path forward and detailed description should be added on how users can retrain their model with their species specific datasets and to what degree the model trained on human samples can be transferred to other species such as plants, fungi, etc.**

## *RESPONSE TO MAJOR SPECIFIC COMMENT 3*

*Thank you for this constructive comment. In this comment, the reviewer raises two core issues: (i) How to retrain (finetune) the EpiGePT model, which has been pretrained on human data, for other species, and (ii) To what degree the model can be transferred to other species, particularly plants or fungi. Overall, this comment is closely related to the content of the first two major comments. Next, we will provide clear explanations addressing each of these core issues individually.*

***1.*** *Regarding Retraining the EpiGePT Model on Other Species*

*We agree with the reviewer that a further discussion and a more detailed description are necessary. Then, we will describe this issue in two aspects: necessary conditions and data preparation.*

*For the necessary conditions, as we have already explained in response to* ***major comment 1****: (i) a high-quality and complete reference genome and (ii) a high degree of conservation in transcription factors (TFs) compared to humans. These two conditions are prerequisites for fine-tuning the model. This is because EpiGePT’s core modeling is focused on learning the relationship between key human TFs and their genomic region binding. If there is low functional and structural conservation of TFs across species, the model cannot effectively learn from this. Therefore, cross-species transfer is only possible when these two conditions are met. For example, organisms such as mouse and chimpanzee are more applicable for cross-species model transfer.*

*For data preparation, researchers need to prepare data from three aspects: experimental epigenomic data, DNA module input, and TF module input.*

1) ***Experimental epigenomic data preparation****. In the corresponding species, researchers need to collect bulk sequencing data from the relevant cell line or tissue. Using DNase-seq as an example, researchers need to first collect the BAM sequencing files and use samtools and bedtools to calculate the read count of the sequencing files over the specified genomic regions (BED file) (refer to the tutorial provided in our GitHub: https://github.com/ZjGaothu/EpiGePT). As for the selection of regions, researchers can either split the entire genome and filter for regions that overlap with epigenomic signals from multiple cell types, similar to our approach, or select specific regions based on their research interests. It is important to note that the length and resolution of the regions need to be consistent with the pretrained human model. For example, the DNase-seq prediction model mentioned earlier uses 10kbp regions with 200bp resolution, while pretrained model for predicting multiple epigenomic profiles uses 128kbp regions with 128bp resolution. Finally, by applying a logarithmic transformation to the read counts, the corresponding ground truth data can be obtained. The ground truth for the i-th epigenomic track in the j-th region can be obtained using the following mathematical expression:*

*𝐸𝑝𝑖	𝑝𝑟𝑜𝑓𝑖𝑙𝑒_!,#_ = log	(𝑟𝑒𝑎𝑑𝑐𝑜𝑢𝑛𝑡_!,#_ + 1)*

2) ***DNA module input preparation****. For the DNA module input, users only need to collect the reference genome fasta file for the corresponding species. For instance, in the case of mouse, the GRCm38 (mm10) version can be used. It is important to note that the fasta file must match the reference genome used in the sequencing experiments (Epigenomic files and RNA-seq files). During model training, the DNA sequence can be read from the reference genome using Pyfasta and then converted into an 𝐿 × 4 one-hot matrix.*
3) ***TF module input preparation****. For the TF module, the input includes expression data for TFs and their binding status to the genomic regions. For the expression data, researchers need to collect RNA-seq data corresponding to the same cell line or tissue associated with the ground truth epigenomic data. If fine-tuning is performed on a specific cell line or tissue, the TPM (Transcripts Per Million) value can be directly utilized and quantile normalized (using the reference TF expression file we provided) to a 711-dimensional TF expression vector. Detailed tutorials on processing these data are available on our GitHub and website*

*(https://github.com/ZjGaothu/EpiGePT, http://health.tsinghua.edu.cn/epigept/tutorial.php). It is important to note that other species may not have a one-to-one correspondence with these 711 human TFs. For missing TF expressions, we can set them to zero or we can replace them with the mean expression of the corresponding TF from the human reference expression profile. For the TF binding status, the DNA binding specificity of TFs is generally highly conserved between related organisms. In mammalian (or even vertebrate) organisms, the human motif files can be used for substitution, utilizing the Homer tool (Heinz, et al., 2010) to scan the TF binding status in the corresponding regions. However, for different sets of organisms, such as fruit flies, yeast, or plants, which often contain entirely different transcription factors, it is necessary to locate the corresponding motif database and then use the Homer tool for scanning (Heinz, et al., 2010). Then, the binding status features for the 711 corresponding TFs can be obtained. By performing an element-wise product of these two features, the input for the TF module during model training can be generated.*

*If the aforementioned conditions are met and the three types of data can be obtained, users can proceed to transfer the model to a new species for fine-tuning. We have incorporated the details of how to transfer the human model to other species in the revised manuscript.*

***2. Transferability of EpiGePT to Other Species****. The ability to transfer the model to other species depends heavily depends on the overall conservation of gene expression profiles between humans and those species. For instance, in species like plants or fungi, where gene expression and regulatory patterns can differ significantly, the primary challenge lies in identifying a set of TFs to model the binding status of TFs to genomic regions across different cell types. Specifically, we collected gene annotation files for widely studied plants from the Ensembl Plants project (Yates, et al., 2022). Taking Arabidopsis thaliana and Oryza sativa, two commonly studied species, as examples, we identified only 26 and 9 corresponding TFs out of the 711 TFs. Overall, among the gene annotation files of the 83 plant species provided by the Ensembl Plants project (Yates, et al., 2022), we found overlap with our selected 711 TFs in only four species, with arabidopsis thaliana exhibiting the highest overlapping proportion of only 3.66% (****Fig. R6a-b****). The small shared TF gene regulation mechanism indicates that transferring human model to plant data is challenging. Fungi exhibit even lower similarity to human TFs in terms of both sequence and function. For example, from FungiDB (AlvarezJarreta, et al., 2024), we retrieved the gene annotation file for Aspergillus aculeatus ATCC 16872, which showed 0% overlap with the 711 human TFs, making it unable to transfer the pre-trained human model to this species. In practice, the degree of overlap in TFs can be used as one criterion for determining the feasibility of model transfer. For organisms such as plants and fungi, where the overlap in TFs is less than 5%, fine-tuning the model is highly challenging. In contrast, vertebrate organisms such as mouse exhibit a high degree of conservation in their overall gene expression profiles compared to humans (Breschi, et al., 2017), which provides a strong foundation for transferring EpiGePT to these species.*

*In summary, we would like to once again thank the reviewer for this constructive comment. We have incorporated a clear path forward and a detailed description of transferring the model to other species into the revised manuscript and supplementary materials. Moreover, we acknowledge that transferring the model to plants or fungi might be not feasible at the moment due to significant differences in gene expression profiles across species. We have also discussed the criteria and conditions for determining whether the model can be transferred to a new species.*

***Fig. R6. The overlap between human and plant gene annotations from the Ensembl Plant project in terms of the 711 selected TFs.*** *(a) The bar plot illustrates the four species (Arabidopsis thaliana, Oryza sativa, Nicotiana attenuata, Hordeum vulgare) that have overlapping TFs with the 711 selected TFs, along with the number of overlapping TFs for each species. (b) The histogram shows the distribution of the number of overlaps with the 711 selected human TFs across 83 plant species.*

## MAJOR SPECIFIC COMMENT 4

**4) Some statements such as "In contrast, each combination of input region and cellular context provides an example for training in EpiGePT, thus providing a much larger number of examples available for model training" need to be clarified in detail. Here it is not clear to me how users who don't have access to TF or tissue data can benefit from their approach.**

## *RESPONSE TO MAJOR SPECIFIC COMMENT 4*

*We thank the reviewer for this thoughtful comment. Below, we provide further clarification regarding both the larger number of examples available for model training and how users can proceed without transcription factor (TF) or tissue-specific data.*

1. *Clarifying the Larger Number of Training Examples.*

*Our statement aims to emphasize the difference in the training methodology between our model and sequence-based methods, such as Enformer, which arises due to differences in input data. Specifically, for predicting epigenomic profiles across multiple cell types, our model uses not only DNA sequence information but also additional TF expression data. This additional information introduces cellular context awareness into the model’s input. As a result, during training, even for the same genomic region, data from different cell types are treated as distinct inputs to the model, effectively increasing the number of training examples. To clarify, we organize the data into an (𝑁 × 𝑀) matrix, where 𝑁 denotes the number of genomic regions and 𝑀 denotes the number of cellular contexts. When our model trains on this data matrix, the total number of training instances is N times M. In contrast, DNA sequence-only LLMs, such as Enformer (Avsec, et al., 2021), use DNA sequence as their sole input. Thus, for the same genomic region across different cellular contexts, their inputs are identical. Taking Enformer as an example, during training on human data, its input consists of approximately 200 kbp of DNA sequence, while its output includes 5,313 human genomic tracks. This means the number of training examples is limited to 𝑁, rather than 𝑁 × 𝑀, which reduces the training instances by 1/M. This highlights the key difference in training methodologies that we aim to emphasize and explains why our approach can lead to a larger number of training examples, which is much preferable to large-scale deep learning models.*

*In addition to increasing the number of training examples, our training strategy provides another advantage: it improves the generalizability of the model when making predictions in new cellular contexts, a feature that DNA sequence-based LLMs like Enformer lack. Although the output of Enformer includes 5,313 human genomic tracks, covering epigenomic profiles and gene expression profiles like CAGE-seq of various cell lines and tissues, it cannot make predictions in any cellular context that was not present in the training set. Enformer can only predict profiles for cell lines or tissues that the model has already seen during the training stage. This limitation in the model’s generalization ability is not aligned with the type of LLM we aim to develop. In contrast, our model only requires the TF expression information of the relevant cell type to perform genome-wide predictions for any novel cellular context. This also leads to the reviewer’s question regarding how users can proceed when they lack TF or tissue data.*

2. *Access to Context-specific TF Expression Data. Regarding the* ***second question*** *on how to use our model EpiGePT in the absence of transcription factor (TF) or tissue-specific data, we would like to provide further clarification. In practical use, we can consider two scenarios:*

***Scenario 1*** *involves users, especially those with biological background, who have a cell line/tissue/celluar context of interest and have already conducted gene expression experiment (e.g., RNA-seq) of this particular context. Note that the expense of RNA-seq is much lower than those epigenomic sequencing experiments. If they want to use EpiGePT to make predictions of epigenomic profiles for that specific cell type and infer the gene regulatory mechanism. In this case, users simply need to follow the tutorial provided on our website (http://health.tsinghua.edu.cn/epigept/tutorial.php) and prepare the model input data for inference. Our website provides the online inference if users uploaded their gene expression data and the genomic regions of interests. Since users have conducted their own sequencing experiments, there will be no issue of lacking TF expression data.*

***Scenario 2*** *involves users who have not conducted gene expression sequencing experiments but want to explore the epigenetic signals of a particular cell line or tissue using public data. To facilitate this issue, we have tried our best to provide a comprehensive TF reference dataset, which covers the major cell types that are publicly available. In most cases, users can easily find the corresponding expression data. For example, in the ENCODE project (Consortium, 2012) has aggregated a vast amount of bulk sequencing data, we compiled data that includes polyA plus RNA-seq and total RNA-seq experiments. We analyzed the distribution of experiments across tissues, cell lines, primary cells, and in vitro differentiated cells, and presented the results in a pie chart (****Fig. R7****). In the human reference genome GRCh37, there are a total of 575 different experimental datasets covering 218 cell lines or tissues. In the reference genome GRCh38, there are 1,015 different experimental datasets that encompass 281 unique cell lines and tissues. Existing research indicates that there are more than 400 cell types in the human body (d Galbraith, et al., 2023). Therefore, we believe that the bulk sequencing data available on ENCODE project has already covered the vast majority of cell types in human body.*

*In addition, with the rapid advancement of single-cell sequencing technology, the scale of single-cell RNA sequencing (scRNA-seq) data has grown rapidly. Expression data from different cell types covered by single-cell data, when processed into pseudo-bulk data, can also be used as model input. For instance, cellxgene database (Biology, et al., 2023) has already collected scRNA-seq data from over 91.7 million human cells. Our statistical analysis of the data, as of September 16, 2024, shows that human data covers 280 tissues and 721 cell types (****Fig. R8*** *and* ***Fig. R9****). Even in the mouse data, the cell count exceeds 42 million, covering 98 tissues and 424 cell types. Therefore, in actual usage scenarios, gene expression data (TPM value) for the vast majority of cell types can be found.*

*If a perfectly matching dataset is not available, users can also select a similar cell type from reference data based on the cell type description to serve as input for the TF module and utilize the model accordingly. We have also provided processed TF expression data for both the hg19 (GRCh37) and hg38 (GRCh38) human reference genomes on the Download page on the website (http://health.tsinghua.edu.cn/epigept/download.php), which can be used as a approximate TF reference profile.*

*In conclusion, regarding the first question about the number of training samples, we have now included the above explanation in the revised supplementary text. For the second question, concerning how to use the model when TF expression data is lacking, we have clarified this through two different scenarios. The situation where users “don’t have access to TF data” is quite rare due to the abundance of available gene expression data from multiple public databases, including GEO database (Barrett, et al., 2012), ENCODE project (Consortium, 2012), ROADMAP project (Bernstein, et al., 2010), FANTOM project (Lizio, et al., 2015) etc. We also recommend that users, in the absence of TF expression data, utilize reference TF expression data to find a similar cell type, enabling them to use the model. We express our appreciation again to the reviewer for posing this thought-provoking question. We have also thoroughly discussed this issue in our response to major comment 8 and provided additional experiments results.*

***Fig. R7. The pie charts illustrate the distribution of RNA-seq data from the ENCODE project.*** *(a) the proportion of experiments for the top 15 cell lines/tissues in the hg19 (GRCh37) reference genome and (b) the proportion of experiments for the top 15 cell lines/tissues in the hg38 (GRCh38) reference genome.*

***Fig. R8. The pie charts illustrate the distribution of human and mouse single-cell RNA-seq data from the CELLxGene database.*** *(a) The top 10 tissues with the highest number of human cells and their respective proportions of total cell count. (b) The top 10 tissues with the highest number of mouse cells and their respective proportions of total cell count.*

***Fig. R9. The pie charts illustrate the distribution of human and mouse single-cell RNA-seq data from the CELLxGene database.*** *(a) The top 15 cell types with the highest number of human cells and their respective proportions of total cell count. (b) The top 8 cell types with the highest number of mouse cells and their respective proportions of total cell count.*

## MAJOR SPECIFIC COMMENT 5

**5) Please also specify how many different tissue samples need to be provided to their model (minimum number of different tissues) to achieve superior prediction accuracy compared to existing methods. In the presented case 129 cellular contexts were processed from the ENCODE project. Are these 129 cellular contexts the minimum number to achieve improvements? If yes, how can users from other model species achieve this high data resolution or will the model also be useful with less cellular context samples? If yes, how?**

## *RESPONSE TO MAJOR SPECIFIC COMMENT 5*

*Thank you for this insightful comment. The core concern raised by the reviewer in this comment is how many cellular contexts are required to achieve improvement. The 129 cellular contexts used by EpiGePT is certainly not the minimum number required to achieve improvements relative to the baseline method. To systematically address this concern, we provide additional experiments by training EpiGePT with different cellular contexts to explain this issue in detail.*

*In addressing the question of how many different tissue samples need to be provided to achieve superior prediction accuracy compared to existing methods, we first need to clearly explain the key differences between our model and existing methods.*

***First****, the motivation behind developing EpiGePT was to create a* ***cellular context-aware*** *model, where the context is represented by the input TF module. This allows the model to accurately predict epigenetic signals given any new cellular contexts. By incorporating the TF module together with the DNA sequence input, EpiGePT largely improves the generalization power by easily making prediction on new cell types while the sequence-based methods fail to do so.*

***Second****, based on the above foundational goal, we will explain step-by-step how EpiGePT differs from other existing methods. The baseline models can be divided into two categories: one consists of pure DNA sequence-based models, and the other includes models that can predict across different cell types. The former includes models like Enformer (Avsec, et al., 2021), while the latter includes models such as ChromDragoNN (Nair, et al., 2019), DeepCAGE (Liu, et al., 2022), and BIRD (Zhou, et al., 2017). Compared to the pure DNA sequence-based models, we achieve superior prediction accuracy because our model’s input data includes not only DNA sequences but also features such as TF binding information in regions and the expression of these TFs. This enables the model to identify the cell type associated with the input, providing it with extra information and giving EpiGePT a clear advantage. Compared to models that predict across cell types, EpiGePT employs a language model approach. Even when making predictions at the token level (genomic bin), it can capture interactions between different bins by the self-attention mechanism. This ability to model interactions across bins is a distinctive feature not present in the other three models, and it is the fundamental reason behind the improvement EpiGePT offers.*

1. *Comparing to Sequence-based Baselines. To further clarify the improvement of EpiGePT over existing methods given different number of cellular contexts, we selected* ***EpiGePT-seq****, the model with performance second only to EpiGePT in predicting DNase-seq data on 1.17 million genomic bins, and compared its performance with EpiGePT under different numbers of training cellular contexts (1, 5, 20, 40, 60, 80, and 100). We evaluated both models on the same 26 test cellular contexts to directly address the question of how many different tissue samples need to be provided to achieve superior prediction accuracy compared to existing methods. Theoretically, when the number of cellular contexts is greater than 1, our model can outperform pure DNA sequence-based methods, such as EpiGePT-seq. As shown in* ***Fig. R10*** *and* ***Fig. R11****, even with only one training cellular context, incorporating the TF module allows our model to achieve a slight advantage in prediction accuracy (1.2% improvement on average in terms of PCC). When the number of cellular contexts exceeds 1, the performance gap becomes larger, with both PCC and SCC showing significant improvements (For example, when trained on 40 cellular contexts, EpiGePT achieves a 5.0% improvement on average in terms of PCC). Impressively, when the number of training cellular contexts reaches 40, the performance of EpiGePT on the test data surpasses that of EpiGePT-seq trained with 100 cellular contexts, and it also exceeds that of Enformer trained with 103 cellular contexts, as reported in Fig. 2b in the original manuscript. This further validates the reasons we previously outlined. Specifically, EpiGePT can provide distinct predictions for different cell types within the same genomic region, whereas sequence-based models like Enformer and EpiGePT-seq can only produce the same prediction for all cell types. Therefore, as long as the number of cellular contexts is greater than one, our model will outperform sequence-only models.*
2. *Comparing to CNN-based Baselines. For other models that can predict across different cell types, the reason EpiGePT achieves superior prediction accuracy lies in its more biologically aligned modeling approach, not in the number of training cellular contexts. EpiGePT learns the comprehensive interactions between different chromatin regions during training by the selfattention mechanism, which may captures the complex regulatory mechanisms of biology better than CNN-based methods. As demonstrated in Fig. 2a in the original manuscript, EpiGePT achieves better performance than the best baseline ChromDragoNN when trained on the same number of cellular contexts. Additionally, as shown in Supplementary Fig. S5a and S5b, EpiGePT still performs better than the best baseline ChromDragoNN with cross-validation when trained on a total of 56 cellular contexts. Moreover, as observed in* ***Fig. R10*** *and* ***Fig. R11****, when the number of training cellular contexts reaches 40, EpiGePT outperforms ChromDragoNN (the best-performing baseline method with cross-cell type prediction capability, trained on 103 cellular contexts in Fig. 2a) on the same test data. This demonstrates that, compared to such methods, the improvement comes from EpiGePT’s modeling approach and architecture rather than the number of cellular contexts.*

*In addressing the question of how can users from other model species achieve this high data resolution or will the model also be useful with fewer cellular context samples, even though the answer to the first question is “no,” we would like to provide a clear explanation to the reviewer here as well. From the perspective of the improvement brought compared to existing methods, our analysis above has demonstrated that it is not affected by the number of cellular contexts. Even in species with fewer cellular context samples, our modeling approach can still achieve better results than existing sequence-based methods. Regarding the model’s dependence on the number of cellular contexts, it is evident from* ***Fig. R10a*** *and* ***Fig. R11a*** *that performance on the same test set increases as the number of cellular contexts rises. However, once the number reaches 40, this improvement trend slows down. In this specific task of predicting human chromatin accessibility (DNase-seq), 40 cellular contexts is the suggested number to achieve a superior prediction performance.*

*In conclusion, while 129 cellular contexts are not the minimum number required to achieve improvements, the core of this improvement lies in the modeling approach. Regarding EpiGePT’s reliance on cellular contexts, having more cellular contexts naturally enhances performance. However, when facing fewer cellular context samples in a specific species, and based on our analysis above, we anticipate that EpiGePT will still outperform existing sequence-based methods or other existing methods trained on the same number of cellular contexts. We have already made the necessary revisions in the revised manuscript and supplementary materials, and we have incorporated the additional experiments mentioned in this response into the revised supplementary materials.*

***Fig. R10. The Pearson correlation coefficient (PCC) for predicting chromatin accessibility (DNaseseq) under different numbers of training cell lines/tissues.*** *(a) Line plot showing the average PCC across 26 test cell types, as a function of the number of training cell types. (b) Box plot showing the distribution of PCC across 26 test cell types for different numbers of training cell types. (c) Bar plot with error bars showing the PCC across 26 test cell types for different numbers of training cell types.*

***Fig. R11. The Spearman correlation coefficient (SCC) for predicting chromatin accessibility (DNase-seq) under different numbers of training cell lines/tissues.*** *(a) Line plot showing the average SCC across 26 test cell types, as a function of the number of training cell types. (b) Box plot showing the distribution of SCC across 26 test cell types for different numbers of training cell types. (c) Bar plot with error bars showing the SCC across 26 test cell types for different numbers of training cell types.*

## MAJOR SPECIFIC COMMENT 6

**6) In lines 130 - 132, the authors write: Finally, we compared EpiGePT and ChromDragoNN [6] in the binary classification of functional regions versus nonfunctional regions, using the functional chromatin status derived from ChromHMM [20] annotations as ground truth (Supplementary Text S6). While ChromHMM is a well established software tool for human data, it would be important to see how the different models (ChromDragoNN, EpiGePT, ChromHMM) benchmark when each model serves as ground truth. E.g. how does EpiGePT and ChromHMM benchmark when ChromDragoNN is used as ground truth, or how does ChromDragoNN and ChromHMM perform when EpiGePT is used as ground truth etc. Please provide a comparison matrix where all combinations are tested**.

## *RESPONSE TO MAJOR SPECIFIC COMMENT 6*

*We appreciate the reviewer for this thoughtful comment. We agree with the reviewer that using other methods as ground truth and benchmarking the remaining methods is a necessary addition. In response, we have made the corresponding revisions by adding performance comparisons of the remaining methods using EpiGePT, ChromDragoNN (Nair, et al., 2019), and DeepCAGE (Liu, et al., 2022) as ground truth for the binary classification task on functional chromatin status. The results are shown in* ***Fig. R12*** *and* ***Fig. R13****. Next, we provide a detailed explanation and interpretation of the results in the figures from three perspectives.*

***First****, we explain the reason for choosing annotations of ChromHMM (Ernst, et al., 2017) as the ground truth in the original manuscript. Since ChromHMM is an unsupervised model, it is not feasible to train it in a supervised manner using sequence label information. ChromHMM utilizes a Hidden Markov Model (HMM) to model DNA sequences across different cell types, with various epigenomic profiles as observed variables, to infer the states of the sequences, which correspond to the model’s hidden states. In the manuscript, we employed the results from its 15-state model, and further merged and simplified the categories. For the binary classification task, we consolidated these states into functional and non-functional chromatin regions. Additionally, the inference results of ChromHMM are based on a large number of epigenomic tracks as inputs to the model, which is not the case for the other supervised machine learning models. This contributes to its increased reliability. ChromHMM has been widely used in the computational biology community for genome-wide annotation of the functional genomic regions. It is natural to use its annotation results as the surrogate “ground truth” for the benchmark experiment. Therefore, using the annotation from ChromHMM as the ground truth to train ChromDragoNN, DeepCAGE, and EpiGePT provides a relatively fair comparison.*

***Second****, regarding the experimental setup for the additional benchmarking. Based on the aforementioned unsupervised nature of ChromHMM, we used ChromHMM’s annotations as the ground truth to train EpiGePT, ChromDragoNN, and DeepCAGE. We converted their predicted results (probabilities) into categorical labels and used these labels as ground truth to evaluate the performance of other methods. For ChromHMM, we downloaded its posterior probabilities from https://egg2.wustl.edu/roadmap/web_portal/chr_state_learning.html to obtain its predicted categories. Consequently, we computed the results for the four methods as ground truth through five-fold cross-validation across 56 cell types. For metric selection, we used accuracy, auROC (area under the Receiver Operating Characteristic Curve), auPRC (Area Under the Precision-Recall Curve), and F1 score.*

***Third****, we conducted comprehensive experiments here by treating the predicted results from one method as ground truth and evaluating the predicted results of other methods. As shown in Fig. R8, we plotted four heatmap matrices, each denoting the results of using different methods as ground truth across the four metrics. Specifically, each row of the matrix represents the performance of the remaining methods using a given method as the ground truth, with the values in each position reflecting the average metric across the 56 cell types in five-fold crossvalidation. The diagonal elements of the matrix are 1, indicating the performance metrics computed with itself as the ground truth. From the figure, it can be observed that EpiGePT consistently outperforms the other three methods across most metrics when using DeepCAGE and ChromDragoNN as ground truth. When using ChromHMM as the ground truth, EpiGePT achieves the highest score across all metrics. The results demonstrate that EpiGePT has a stronger ability to learn from the chromatin state information contained in the ground truth labels and performs better in predictions when ground truth is available. Additionally, we found that EpiGePT continues to achieve best performance when using DeepCAGE as the ground truth. However, when using ChromDragoNN as the ground truth, EpiGePT is only slightly worse than DeepCAGE but better than other methods. These results suggest that the predicted labels of EpiGePT are less similar to those of ChromDragoNN compared to DeepCAGE. In other words, compared to ChromDragoNN and DeepCAGE, EpiGePT is capable of predicting many common functional chromatin regions, while also correctly identifying regions that the other two methods misclassified. This highlights the accuracy of EpiGePT’s predictions.*

*To further illustrate all combinations when each model serves as ground truth, we also plotted box plots (****Fig. R13****) showing the performance metrics of other methods across each cell type when each model is used as the ground truth. We specifically generated box plots for the results using EpiGePT, ChromDragoNN, and DeepCAGE as ground truth, and the conclusions drawn from these plots are consistent with those in* ***Fig. R12****.*

***In conclusion****, in response to the constructive comments, we conducted a detailed analysis of how the different models (ChromDragoNN, EpiGePT, ChromHMM, and DeepCAGE) perform when each model serves as the ground truth. This comprehensive evaluation of the predictive performance across models not only illustrates the differences between them but also emphasizes the superior accuracy and robustness of EpiGePT across various metrics and cell types.*

***Fig. R12. The average predictive performance of the remaining methods when each model serves as the ground truth.*** *(a) Heatmap of average accuracy when the four methods serve as ground truth. The rows represent the ground truth, the columns represent the prediction methods, and the values indicate the five-fold cross-validation averages across all test cell types. (b) Heatmap of average auROC when the four methods serve as ground truth. (c) Heatmap of average auPRC when the four methods serve as ground truth. (d) Heatmap of average f1-score when the four methods serve as ground truth.*

***Fig. R13. The box-plots of predictive performance of the remaining methods when each model serves as the ground truth.*** *(a)* *Box plot of the performance (accuracy, AUROC, AUPRC, F1-score) of the remaining models when ChromDragoNN serves as the ground truth. (b)* *Box plot of the performance (accuracy, AUROC, AUPRC, F1-score) of the remaining models when DeepCAGE serves as the ground truth. (c)* *Box plot of the performance (accuracy, AUROC, AUPRC, F1-score) of the remaining models when EpiGePT serves as the ground truth.*

## MAJOR SPECIFIC COMMENT 7

**7) In lines 140 - 142 the authors write: EpiGePT demonstrates a 3.3% to 5.2% higher performance than Enformer in terms of the median Pearson correlation coefficient under the three prediction settings (Fig. 2b). While this improvement is rather incremental and not substantial, to make any outperformance claim, the Enformer model would have to be retrained using the same training dataset used for EpiGePT training. It was not clear to me whether or not this has been done?**

## *RESPONSE TO MAJOR SPECIFIC COMMENT 7*

*Thank you for this thoughtful comment. In fact, in order to have a fair comparison with Enformer, we have adopted two different strategies for implementing Enformer models. 1) we directly use the pretrained Enformer from the original paper; 2) we retrained Enformer model from scratch using the same amount of training data as other sequence-based methods, such as EpiGePT-seq. Let us now provide a detailed explanation to the reviewer.*

1. ***Comparing with retrained Enformer****. We conducted extensive experiments by comparing our EpiGePT model to the retrained Enformer model where the Enformer was trained from scratch. The results shown in* ***Fig. 2b*** *represent our comparison with the retrained Enformer. In* ***Fig. 2b****, we compare our model with Enformer on chromatin accessibility prediction tasks, under three experimental settings: “cross-region,” “cross-cell type,” and “cross-both.” However, due to the pure DNA sequence as input, Enformer lacks the capability to perform predictions in the “cross-cell type” and “cross-both” settings. This is because Enformer’s input consists solely of DNA sequence information, without any cell-type-specific information, and it predicts numerous genomic tracks simultaneously through a multi-task framework. Consequently, for the same genomic region, it can only predict the genomic tracks that were present in the training data and cannot generalize to new cell types. Therefore, to ensure a fair comparison, we adopted Enformer’s architecture as per its original method (Supplementary Text S5). We then retrained Enformer on the same dataset to obtain the retrained Enformer model for comparison. Therefore, we have already compared our model with the retrained Enformer and confirmed that EpiGePT demonstrates improvements in prediction performance across all three experimental settings.*
2. ***Comparing with pretrained Enformer.*** *However, we also realized that training a new Enformer model from scratch is either time-consuming or GPU resource-extensive, which might not be feasible for many small labs. Many researchers may directly use the pretrained version provided in the original research. The Enformer paper also provided users with userfriendly API to perform inference using the pretrained model. Note that the pretrained Enformer model were based on 5,313 human experiments (e.g., DNase-seq, ChIP-seq etc) from the ENCODE, which is a more “favorable” setting to Enformer. Specifically, we evaluated the overlapping genomic tracks between our test dataset and the 5,313 human genomic tracks predicted by Enformer, then compared the performance of the pretrained Enformer with our EpiGePT on these overlapping tracks. As shown in* ***Fig. 2d****, on 78 genomic tracks from 19 cell types that EpiGePT had not seen during training, EpiGePT still achieved an average* ***6.0%*** *improvement in Spearman correlation coefficient (SCC) and* ***7.0%*** *improvement in Pearson correlation coefficient (PCC). Note that these genomic tracks were already present in the training set of Enformer, indicating that the out-of-sample prediction of EpiGePT is still better than the in-sample prediction of pretrained Enformer model. This further demonstrates that even when compared to the pretrained Enformer, EpiGePT still delivers superior performance. One fundamental reason for the improvement of EpiGePT over Enformer lies in the different model design. Enformer aims to directly predict epigenomic signals from only DNA sequences ignoring the fact that the DNA sequence remains the same across different cellular contexts, but gene regulation and expression patterns differ. Our EpiGePT model explicitly model the TF-based gene regulation, thus potentially providing a better interpretation of how the epigenomic signals are determined by both DNA sequence and the corresponding cellular context.*

*In summary, we have conducted comparisons with both the pretrained and retrained Enformer models, and demonstrated performance improvements in both scenarios. This more comprehensively illustrates that for capturing diverse epigenomic features across multiple cellular contexts, EpiGePT is a more suitable modeling approach. In the “Genome-wide prediction of epigenomic signals” section of the Results in the manuscript, we have provided a detailed comparison with the retrained Enformer and the pretrained Enformer in the second paragraph.*

## MAJOR SPECIFIC COMMENT 8

**8) In contexts where TF profiles or chromatin interaction data are sparse or unavailable the authors do not discuss how their model's predictive accuracy may change or even suffer.**

**Please discuss this point in detail.**

## *RESPONSE TO MAJOR SPECIFIC COMMENT 8*

*We appreciate the reviewer for this insightful comment. We first apologize for not thoroughly discussing how to use the model for predictions when TF profiles or chromatin interaction data are sparse or unavailable, and how this might impact the model’s predictive accuracy, and we agree with the reviewer that this is an important consideration for the model’s application. Next, we will provide detailed explanations on how the model can be used for predictions in scenarios where TF profiles or chromatin interaction data are lacking.*

***For scenarios where TF profiles are sparse or missing****, we can divide the discussion into three cases: (1) The user has TF profiles, but they are incomplete, in which case we will address how the degree of sparsity affects predictive accuracy; (2) The user does not have the TF profiles for the specific cell type but can find a similar cell type with reference TF profiles available; and (3) The user lacks TF profiles for the target sample but can obtain TF profiles from the same cell type in other samples. We will explain how to use the model in each of these cases.*

1) ***For the first case****, we have conducted an experiment on chromatin accessibility profile (DNase-seq) prediction task in 26 novel cell types to investigate the effects of sparsity of TF data (****Fig. R14****). Specifically, for 711 TFs, we evaluated the performance of pre-trained EpiGePT under the absence of 10, 50, 200, and 500 randomly selected TFs. Additionally, during the prediction stage, we explored three potential strategies to address missing TF data: (1) relacing the profiles of the missing TFs with 0; (2) replacing the missing TF profiles with the average values from the reference TF profiles; and (3) replacing the missing TF profiles with the median values from the reference TF profiles (Note: since not all test cell lines/tissues have similar counterparts in the reference TF profiles, this case is not discussed here but will be addressed in detail in the second case). As shown in* ***Fig. R14a-R14c****, the scatter plots demonstrate the predicted PCC values on the test cell line before and after replacing the TF profile. The x-axis denotes the PCC values using the complete TF profile, while the y-axis denotes the PCC values using the sparse TF profile. Each point corresponds to a specific cell line/tissue. The results firstly indicate that, among the three potential strategies to address missing TF data,* ***replacing missing values with the average of reference profiles yields the best performance on average****. Additionally, with respect to sparsity, when a small number of TFs are missing (10 or 50), the model’s predictive accuracy is not significantly affected. For instance, the average PCC decreases by only* ***0.8%*** *when replacing 10 TF profiles with the average values (****Fig. R14b****). When a large number of TF profiles are missing (200, 500), the average PCC shows a more clear decline. Specifically, when 500 TFs were missing and replaced with zeros, the average PCC dropped from 0.762 to 0.464. However, by replacing the missing profiles with the average reference TF expression, the PCC only decreased to 0.719. In other words, while predictive accuracy is significantly impacted when a large portion of TF profiles is absent, incorporating reference TF profiles allows the model to maintain a reasonable level of predictive performance.*
2) ***For the second case****, we demonstrate the impact of using TF profiles from similar cell types by conducting replacement experiments on three sets of closely related cell types. Specifically, we examined (1) the left lung and lower lobe of the right lung, (2) the heart right ventricle and right cardiac atrium, and (3) the gastrocnemius medialis and tibial nerve. These cell types were selected as they belong to the same respiratory system, are components of the heart, and share anatomical proximity in the thoracic cavity, respectively. For each pair, we replaced the 711 TF profiles with those from a similar cell type, reference median values, and reference mean values (****Fig. R15****). The results indicate that for eight types of epigenetic modification signals,* ***using TF expression profiles from similar cell lines or tissues generally leads to better predictive performance compared to using median or mean values as substitutes****. For instance, when the TF expression profile of the tibial nerve was used to replace that of the gastrocnemius medialis, the predicted PCC for the DNase-seq profile dropped from 0.901 to 0.860, whereas using mean values reduced it further to 0.785. Similarly, replacing the heart’s right ventricle TF profile with that of the right cardiac atrium resulted in only a slight decrease in the predicted PCC for H3K4me3 from 0.680 to 0.675, while using mean values caused a more significant drop to 0.557 (****Fig. R15b, Fig. R15c****). These results indicate that while the complete absence of the target cell type’s TF profile can have some impact on predictive accuracy, it does not significantly compromise the model’s overall performance. Selecting TF profiles from similar cell types based on anatomical relationships, tissue types, or broader system-level relationships tends to minimize the reduction in predictive accuracy.*
3) ***For the third case****, we examined the impact of replacing TF profiles with those from different samples of the same tissue type on the model’s predictive accuracy. Specifically, we selected two sets of TF profiles from different sample batches of the same tissue type, sourced from the ENCODE project, namely the left renal pelvis (ENCSR029FTY and ENCSR410DUZ) and the renal cortex interstitium (ENCSR899SWV and ENCSR436ZKE). These cell types were included in the test set data. We then used both sets of TF expression profiles as model inputs and employed the matching DNase-seq data from the samples as ground truth to simulate the scenario where TF profiles from different samples of the same cell type are used (****Fig. R16****). We compared the differences in predicted values across 1.17 million genomic bins before and after the replacement of TF profiles in each pair of data sets, and visualized these differences using density plots (****Fig. R16a, R16b****). Based on the density plots, when using TF profiles from different donors, the predicted values show a high degree of overlap. From the scatter plots, predictions using matched TF profiles show slightly better accuracy, while using TF profiles from other samples leads to a slight decrease in predictive accuracy, by 1.8% and 1.1% in the two tissues, respectively. Overall,* ***if TF profiles from other samples of the same cell type can be found, the model’s predictive accuracy will not experience a significant decline****.*

*Therefore, in scenarios where TF profiles are unavailable or sparse, we have explored three different cases and presented solutions for each. Researchers can practically seek similar cell types, different samples of the same cell type, or utilize reference TF profiles we provided, all of which can facilitate model predictions with only a slight reduction in predictive accuracy. Additionally, in our response to major comment 4, we have also demonstrated the substantial accumulation of publicly available scRNA-seq data. Thus, we believe that researchers can find corresponding or similar TF profiles for cell types in the vast majority of cases, indicating that this issue will not limit the generalizability of the model and predictive accuracy of the model.*

***For scenarios where chromatin interaction data are sparse or missing****, the model’s predictive accuracy during the inference phase will not be affected by data sparsity or absence. This is because chromatin interaction data are only used during training to guide the model in learning potential regulatory relationships. It acts as a constraint applied to the model’s attention layer and guide the model learning through an additional loss term, without being part of the model’s input. Specifically, we introduced a cosine similarity loss on the attention scores corresponding to regions with chromatin interactions, in addition to the model’s original loss function:*

*𝐿 = 1𝐽 >)#’( |𝐵1!| >&$’( ||𝑦!,#,$ − 𝑦C!,#,$||%% ∙ 𝐼(𝑘 ∈ 𝐵!)*

*This added constraint encourages the model to assign higher attention scores to bins that are known to interact with each other. Since chromatin interactions are inherently sparse, this additional loss only influences the model’s backpropagation in a limited number of regions.* ***Therefore, during the inference stage, no chromatin interaction data are required as model input.*** *In other words, when users wish to make predictions for a specific cell type, they only need to provide the DNA sequence information and the TF expression profile, without needing any chromatin interaction data. Users do not need to be concerned with preparing corresponding chromatin interaction data, as this was taken into account during model development to enhance both the model’s generalizability and ease of use.*

*In summary, we have simulated scenarios where TF profiles and chromatin interaction data are missing, and investigated their impact on EpiGePT’s predictive accuracy. For the former, we provided different solutions tailored to specific application cases, enabling predictions with minimal impact on predictive accuracy. For the latter, we clarified that chromatin interaction data do not serve as input of the model, and therefore do not affect prediction or inference.*

*Additionally, we have discussed these aspects in the revised supplementary materials and revised manuscript.*

***Fig. R14. The simulation evaluated the changes in PCC for cross-cell type predictions under different numbers of missing TF profiles with three replacement strategies.*** *(a)* *The average PCC for 26 test cell lines/tissues after replacing different numbers (10, 50, 200, 500) of TF profiles with the median value of the reference profile. In the scatter plot, the x-axis represents the performance before replacement, and the y-axis represents the performance after replacement. (b)* *The average PCC for 26 test cell lines/tissues after replacing different numbers (10, 50, 200, 500) of TF profiles with the average value of the reference profile. (c)* *The average PCC for 26 test cell lines/tissues after replacing different numbers (10, 50, 200, 500) of TF profiles with the zeros.*

***Fig. R15. In three sets of similar cell types, the changes in predictive performance of eight types of epigenomic signals were simulated under different replacement strategies (similar, mean, median) when TF profiles were missing.*** *(a) Eight-line charts show the predictive PCC for eight different epigenomic signals within the pair of similar cell types, the left lung and lower lobe of the right lung. The charts compare the PCC for predictions using the original TF profile (matched), the TF profile replaced with a similar cell type’s profile (similar), and the TF profiles replaced with the reference profile’s mean and median values. (b) The change of predictive PCC for eight different epigenomic signals within the pair of similar cell types, the heart right ventricle and right cardiac atrium. (c) The change of predictive PCC for eight different epigenomic signals within the pair of similar cell types, the gastrocnemius medialis and tibial nerve.*

***Fig. R16. In the scenario of two TF profiles from the same cell type but different samples, the changes in DNase-seq predictive performance are simulated when missing TF profiles are replaced with the TF profile from a different sample.*** *(a) The density plot (left) illustrates the predicted values before and after replacing the TF profile of the left renal pelvis from experiment ENCSR029FTY with that from experiment ENCSR410DUZ, and the scatter plot (right) shows the predicted values from both TF profiles against the ground truth, with the x-axis representing the true values and the y-axis representing the predicted values. (b) The density plot (left) illustrates the predicted values before and after replacing the TF profile of the renal cortex interstitum from experiment ENCSR899SWV with that from experiment ENCSR436ZKE, and the scatter plot (right) shows the predicted values from both TF profiles against the ground truth, with the x-axis representing the true values and the y-axis representing the predicted values.*

## MAJOR SPECIFIC COMMENT 9

**9) Training and running predictions with EpiGePT may require substantial computational resources, which may limit its accessibility to researchers with limited access to highperformance computing facilities. It would be paramount that the authors provide more details on the time computational complexities required to train and employ their models.**

## *RESPONSE TO MAJOR SPECIFIC COMMENT 9*

*We appreciate the reviewer for this constructive comment. We agree with the comment of reviewer that training EpiGePT may present a high computational resource requirement, potentially posing a barrier for researchers. However, in practice, the computational demands for users performing inference with EpiGePT are significantly lower. I will now explain the computational requirements for EpiGePT inference from three perspectives.*

***First****, we recorded the computational resource requirements during both model training and inference. The total parameter size of EpiGePT is approximately 71.3M, making it feasible to train and infer even using standard GPU hardware. To provide a more comprehensive overview of the resource consumption during pre-training, we additionally recorded the resource usage when training on three different CPU and GPU configurations (NVIDIA GeForce RTX 3090, NVIDIA GeForce RTX 4090 and NVIDIA RTX A6000), each using a single GPU (****Table R1R3****,* ***Fig. R17****). Specifically, using a single NVIDIA RTX A6000 as an example, which has 48GB of memory, we observed a peak GPU memory usage of 31,792MB with a batch size of 16. Under these conditions, training one epoch takes approximately 14 hours. The training data for this experiment included 85 cell lines or tissues, covering 15,870 128kbp regions, meaning the model was trained on roughly 15 million bins. These results indicate that, in terms of computation time, completing model training on a single A6000 GPU would take approximately 2-3 weeks. However, this process can be accelerated by utilizing multiple GPUs in parallel (via DP or DDP strategies). In terms of memory usage, GPUs with 48GB or 24GB of memory are sufficient to support the pre-training of EpiGePT. In terms of memory usage, we load all 15 million motif scores and the corresponding label information for 104 cell lines/tissues across these bins into memory at once to improve dataloader efficiency, resulting in a peak memory usage of 341.529GB. However, during practical use, the training samples can be stored in segments on disk. By streaming data from disk during the training process, this approach can significantly reduce the physical memory requirements. Therefore,* ***we conclude that training can be efficiently performed on a system equipped with a single GPU with 24GB of memory and 128GB of RAM****, without the necessity of extensive reliance on highperformance computing clusters.*

*For researchers, the practical demand typically centers on performing inference with the pretrained model rather than retraining it from scratch. Consequently, we focus more on the memory requirements associated with the inference process. During the inference process, as backpropagation is not required to adjust the model weights, researchers need only ensure that the GPU is capable of performing inference with a batch size of at least 1. Our testing indicated that the GPU memory usage for a batch size of 1 is only 3,012MB, a requirement that is readily accommodated by the majority of GPUs currently available. Regarding memory usage, it is contingent upon the size of the sample set to be predicted. If the sample size is excessively large, the same approach of segmenting the data on disk can be employed to reduce memory consumption. Therefore, for researchers, the computational resource requirements for performing inference with EpiGePT locally are not so demanding.*

***Second****, to facilitate the use of EpiGePT for researchers without enough computational resources, we further developed an online prediction platform for EpiGePT (http://health.tsinghua.edu.cn/epigept). We have deployed the pre-trained EpiGePT model on the backend of the platform to assist researchers who lack programming expertise or computational resources. We have pre-scanned and stored the whole-genome motif scores (hg19 and hg38), organizing them by chromosome and storing them on computing nodes within a high-performance computing cluster. By simply inputting cell type and genomic region information, users can easily obtain predictions of epigenomic signals. Specifically, we provide two options for users to input their data: single region prediction and multiple regions prediction. For the former (single region prediction), users can select a specific genomic region and upload the expression data of relevant transcription factors (TFs) for the desired cell type to complete the prediction. For the latter (multiple regions prediction), users can upload a BED file to obtain predictions for multiple genomic regions simultaneously. We support predictions based on two reference genomes, hg19 and hg38. Additionally, users can provide their email addresses, and upon completion of the computational tasks, an email containing a download link for the results will be automatically sent. Furthermore, we offer a comprehensive tutorial on how to prepare the input TF expression file (http://health.tsinghua.edu.cn/epigept/tutorial.php). For those who wish to run our model locally on their own machines, we also provide detailed tutorials, and all processed data used in the predictions are available for download on the web server’s Download page. By setting up this web server, we aim to help users perform inference more conveniently and efficiently. By establishing this web server, we aim to provide users with a more convenient and efficient way to perform model inference. Therefore, to address the challenge of efficiently employing EpiGePT,* ***we have developed a web server that enables online inference, providing users with a solution to this issue****.*

***Third****, in response to the comment of reviewer, we created a* ***Docker image*** *for the environment for running EpiGePT. Users can pull the environment to their local computer using the command “docker pull epigept/epigept:latest,” allowing for further application and development of EpiGePT.*

*In conclusion, we have provided more detailed explanations of the computational complexities for both the training and inference EpiGePT. We also clarified that both training and inference are feasible on most computers equipped with at least one GPU device. Additionally, we undertook two initiatives to facilitate researchers’ use of our model: first, by setting up a web server for online predictions, and second, by creating a Docker image, enabling researchers to either perform analyses online or deploy the development environment locally for further analysis.*

***Table R1****. The computational resource usage, including peak GPU memory and peak RAM consumption (NVIDIA GeForce RTX 3090).*

| *Batch size* | *GPU Device* | *Time*  *(h)/epoch* | *Peak GPU*  *memory (MB)* | *Peak RAM*  *memory (GB)* |
| --- | --- | --- | --- | --- |
| *2* | *NVIDIA GeForce RTX 3090* | *19* | *6356* | *341.529* |
| *4* | *NVIDIA GeForce RTX 3090* | *15-16* | *10118* | *341.529* |
| *6* | *NVIDIA GeForce RTX 3090* | *13-14* | *13860* | *341.529* |
| *8* | *NVIDIA GeForce RTX 3090* | *13* | *17526* | *341.529* |
| *10* | *NVIDIA GeForce RTX 3090* | *13* | *21210* | *341.529* |
| *CPU info: Intel(R) Xeon(R) CPU E5-2680 v4 @ 2.40GHz* | | | |  |

***Table R2****. The computational resource usage, including peak GPU memory and peak RAM consumption (NVIDIA GeForce RTX 4090).*

| *Batch size* | *GPU Device* | *Time*  *(h)/epoch* | *Peak GPU*  *memory (MB)* | *Peak RAM*  *memory (GB)* |
| --- | --- | --- | --- | --- |
| *2* | *NVIDIA GeForce RTX 4090* | *15* | *6510* | *341.532* |
| *4* | *NVIDIA GeForce RTX 4090* | *12* | *10280* | *341.532* |
| *6* | *NVIDIA GeForce RTX 4090* | *11* | *14022* | *341.532* |
| *8* | *NVIDIA GeForce RTX 4090* | *10-11* | *17680* | *341.532* |
| *10* | *NVIDIA GeForce RTX 4090* | *9-10* | *21390* | *341.532* |
| *CPU info: Intel(R) Xeon(R) Gold 5220 CPU @ 2.20GHz* | | | |  |

***Table R3****. The computational resource usage, including peak GPU memory and peak RAM consumption (NVIDIA RTX A6000).*

| *Batch size* | *GPU Device* | *Time*  *(h)/epoch* | *Peak GPU*  *memory (MB)* | *Peak RAM*  *memory (GB)* |
| --- | --- | --- | --- | --- |
| *2* | *NVIDIA RTX A6000* | *19* | *6356* | *341.529* |
| *4* | *NVIDIA RTX A6000* | *16-17* | *10126* | *341.529* |
| *8* | *NVIDIA RTX A6000* | *14-15* | *17526* | *341.529* |
| *10* | *NVIDIA RTX A6000* | *14-15* | *21210* | *341.529* |
| *16* | *NVIDIA RTX A6000* | *14* | *31792* | *341.529* |
| *CPU info: AMD EPYC 7643 48-Core Processor* | | |  |  |

***Fig. R17.*** *The computational resource usage, including peak GPU memory and time for training an epoch on an NVIDIA GeForce RTX 4090. (a) Line plot showing the GPU memory usage (in MB) as a function of batch size. (b) Line plot showing the time taken to train one epoch (in hours) as a function of batch size.*

## MAJOR SPECIFIC COMMENT 10

**10) Given the complexity of the model and the extensive training on specific tissue datasets, there is a potential risk of overfitting to the training data, which may affect the model's performance on truly novel data. How do the authors avoid overfitting?**

## *RESPONSE TO MAJOR SPECIFIC COMMENT 10*

*We appreciate the reviewer for this thoughtful comment. Indeed, we agree with the reviewer that there is a potential risk of overfitting to the training data. However, we have adopted three specific strategies during the model training process in order to minimize the risk of overfitting.* ***First****, during model training, we divided the data into a training set and a validation set. Specifically, we randomly split the data in a 9:1 ratio for training and validation. At the end of each epoch, we calculated the loss on the validation set, and we implemented an early stopping mechanism based on these losses. If the loss on the validation set did not decrease after 3 consecutive epochs, the training process was stopped. The model with the best performance on the validation set was retained for downstream analysis. It is important to note that the validation set contained different combinations of cell types and genomic regions compared to the training set, which helped improve the generalization ability of the model and reduce the risk of overfitting. Additionally, we conducted a five-fold cross-validation in the benchmark experiment for chromatin accessibility (DNase-seq profiles) prediction to more comprehensively validate the model’s performance on truly novel data.*

***Second****, we incorporated dropout during model training. Throughout the training process, we used the dropout technique by randomly setting the output of certain neurons to zero with a probability of p , effectively perturbing the network structure of the model. This forces the model to rely on only a subset of neurons during each forward pass, making it more difficult for the model to become dependent on specific neurons. As a result, dropout helps prevent the model from overfitting to the training data.*

***Third****, during model training, we randomly shuffled the order of the input samples. Our training data consists of a matrix that includes multiple cell lines/tissues and genomic regions, so the order in which data is fed into the model is particularly important. During training, we randomly selected different cell lines/tissues and genomic regions, shuffling the data matrix along both rows and columns. This approach ensures that the model is not trained with a fixed anchor, such as a specific cell line/tissue or genomic region, further promoting generalization and mitigating the risk of overfitting. This training approach also helps prevent the model from memorizing the specific order of the training data, breaking its dependence on the cell line/tissue information or the chromatin positional information of the regions. Consequently, this enhances the model’s generalization ability and reduces the risk of overfitting.*

*In summary, we have implemented three strategies during the training process to minimize overfitting: partitioning the validation set and applying an early stopping mechanism, incorporating dropout, and randomly shuffling the order of cell types and genomic regions. The model’s performance on the test set further confirms the effectiveness of these measures in mitigating overfitting.*

**REVIEWER #2:**

**Gao et. al. presented EpiGePT, a deep learning model using DNA sequence and epigenetic profiles (TF binding and chromatin interactions) to pre-train and perform multiple downstream tasks including the prediction of epigenetic tracks, chromatin interactions, TF binding, variant effect prediction and so on. For each of these downstream analyses, the authors presented a comprehensive performance assessment with reference datasets and state-of-the-art methods. The manuscript is well written and the methodology and results are well described. I believe the methods, results and associated source code and webserver can be valuable to the community. I'd recommend accepting the manuscript but request the authors to address the mentioned comments for a thorough performance analysis and also recommend the most useful model for future use.**

## *RESPONSE*

*We appreciate the reviewer for recognizing the significance of our work and the positive comments. In response to the reviewer’s comments, we have made comprehensive revisions to the manuscript. Next, we response to the reviewers’ concerns in a point-to-point manner.*

## MAJOR SPECIFIC COMMENT 1

**1) The current manuscript proposes multiple models and settings: EpiGePT, EpiGePT(seq) excluding the TF based learning, EpiGePT (3D: setting B) which incorporates 3D interactions, EpiGePT(3D: setting A) which uses self-attention scores for predicting E-P interactions, and EpiGePT(3D: finetuned) which basically fine-tunes EpiGePT for predicting 3D interactions. Different settings have different utility. However, for simplicity and future use by the community, the authors should propose one particular setting of EpiGePT for predicting epigenomic tracks and chromatin interactions, and present its performance in all of the mentioned objectives.**

## *RESPONSE TO MAJOR SPECIFIC COMMENT 1*

*We appreciate the reviewer for this thoughtful comment. We agree with the reviewer that the original manuscript presented a number of EpiGePT settings, which may have caused confusion regarding how to select a particular setting of EpiGePT for downstream applications. Below, we will provide a detailed explanation of our model and the purpose and distinctions of the various settings.*

*To provide a clearer explanation of the purposes, inputs, and outputs of the different settings, we have organized the models and settings mentioned by the reviewer into a table, categorizing them into* ***Epigenomic Profiles Prediction*** *and* ***Chromatin Interactions Prediction*** *based on their respective tasks. As shown in* ***Table R4****, our models and different settings are divided into two parts: EpiGePT and EpiGePT-seq are models for predicting epigenomic profiles, while the other settings, A-C, are designed to explore the model’s ability to capture chromatin interactions.*

***For epigenomic profiles prediction****, we need to firstly emphasize that for future usage, we provide users with the pretrained EpiGePT as the base model. The main purpose of designing EpiGePT-seq is to perform an ablation study to validate the improvement brought by incorporating TF profiles into the model, rather than to offer it as a pretrained model for users. Therefore, we have sufficiently compared the performance of EpiGePT with EpiGePT-seq, which uses only DNA sequence as input. As shown in* ***Fig. 2a*** *and* ***Fig. S3b*** *of the original manuscript, EpiGePT consistently achieved better results across three tasks related to predicting epigenomic signals, such as chromatin accessibility measured by DNase-seq. Additionally, we added the experiments with cross-cell-type predictions using human data, where we downsampled the training cellular contexts and evaluated the models on the same test cellular context. In all cases, EpiGePT outperformed EpiGePT-seq under various numbers of training cellular contexts (****Fig. R10a-c****). Furthermore, we fine-tuned the models on data from three mouse cell types using EpiGePT and EpiGePT-seq, which were pretrained on 100 cellular contexts. The results showed that in brain, kidney, and lung cellular contexts, EpiGePT achieved superior performance even with limited mouse data (****Fig. R5****). These findings demonstrate that EpiGePT, with the integration of the TF module, not only improves performance on epigenomic profiles prediction tasks but also brings the model with the capability for predicting in unseen cell types. This is the primary distinction between EpiGePT and current methods based solely on DNA sequences. Additionally, we provided a performance comparison for cross-cell-type prediction on DNase-seq profiles (hg19 reference genome) (****Fig. R18****), which includes EpiGePT, EpiGePT-seq, BIRD, ChromDragoNN, Enformer, and Enformer+. It is evident that EpiGePT achieves the best performance, and this figure clearly demonstrates the improvement that EpiGePT brings over baseline methods, particularly in our key task of cross-cell-type prediction. In summary, the performance comparison for the* ***epigenomic profiles prediction task was conducted among EpiGePT, EpiGePT-seq, and baseline methods****. This task does not involve the three models (settings A-C) used for chromatin interaction prediction. The performance comparisons have already been presented in the original manuscript (****Fig. 2a, Fig. S3b, Fig. R10, Fig. R5, Fig. R18****).*

***For chromatin interactions prediction****, we presented three different settings. However, these settings are based on the same model architecture, with identical forward propagation processes and model inputs. The difference lies in the approaches: settings A and B use self-attention scores to prioritize chromatin interactions, while setting C replaces the model’s output components with a new MLP to serve as a classifier, predicting whether the two anchors have an interaction relationship.*

*Overall, the three settings are intended for two different application scenarios.* ***First****, in situations where real label data is lacking, such as cell types without 3D genome data, settings A and B can be used. For these two models, we recommend using EpiGePT-3D (setting B) for chromatin interaction prediction. In the manuscript, we have demonstrated through the comparison of setting A and setting B that, when training the model on the same data, EpiGePT3D exhibits more biologically meaningful self-attention scores than EpiGePT, allowing it to better prioritize real chromatin interactions (****Fig. 3d-e****). Second, in scenarios where real label data is available but there is a need to predict additional chromatin interaction pairs, the finetuned model is a more accurate choice. However, the output of the finetuned EpiGePT is not epigenomic profiles, nor is it designed to predict epigenomic signals; therefore, it is unnecessary to compare it with EpiGePT in the epigenomic profiles prediction task. In the manuscript, we provided a comparison between the finetuned EpiGePT and baseline methods (DeepTACT, Kmer-MLP), showing that the finetuned EpiGePT outperforms the baselines. Additionally, we supplemented the comparison with data lifted over to hg38, showing that EpiGePT-3D still provides performance improvement over EpiGePT and other baseline methods (****Fig. R21-R22****). In summary, across both application scenarios and under the same training settings, EpiGePT-3D consistently achieves better performance than EpiGePT.*

***Therefore, we recommend using EpiGePT-3D for chromatin interaction prediction****.*

*In addition, regarding the performance of EpiGePT-3D and EpiGePT on the epigenomic profile prediction task, we have validated that, after training on the same 11 cellular contexts, EpiGePT can improve the average predicted PCC across all genomic tracks from 0.407 to 0.439 when tested on 19 different cellular contexts (****Fig. 3e****). However, the currently available chromatin interaction data with corresponding RNA-seq, ChIP-seq, and DNase-seq data for the same cellular contexts is limited. This constraint impacts the performance of models trained on a larger number of cellular contexts. Therefore, for the epigenomic profile prediction task, we provide pretrained versions of EpiGePT for both hg19 and hg38 reference genomes for researchers, as well as an EpiGePT-3D model trained on 11 cellular contexts available on our website (http://health.tsinghua.edu.cn/epigept). Additionally, we plan to update the EpiGePT3D on our website when more comprehensive 3D genome data becomes available in the future. All the aforementioned revisions and results have been incorporated into the corresponding sections of the manuscript. We express our appreciation once again for the reviewer’s thoughtful comment, which have helped improve the clarity and logic of the explanations regarding the different model settings.*

***Table R4.*** *Description and comparison of EpiGePT with different settings.*

| ***Model Input*** | ***Output*** | ***Purpose*** |
| --- | --- | --- |
| *Task: Epigenomic profiles prediction* |  |  |
| *EpiGePT DNA + TF* | *Genomic tracks* | *Predicting epigenomic profiles.* |
| *EpiGePT-seq DNA* | *Genomic tracks* | *Ablation study* |
| *Task: Chromatin interactions prediction* |  |  |
| *EpiGePT (setting A) DNA + TF* | *Attention scores* | *Prioritizing chromatin interactions* |
| *EpiGePT-3D (setting B) DNA + TF* | *Attention scores* | *Prioritizing chromatin interactions* |
| *EpiGePT-finetuned DNA + TF*  *(setting C)* | *Predicted probability of chromatin*  *interaction* | *Predicting chromatin interaction* |

***Fig. R18. Comparison of cross-cell-type chromatin accessibility (DNase-seq profiles) prediction performance between EpiGePT and baseline methods.*** *(a) Box plot of Pearson correlation coefficients (PCC) of the 5-fold cross-validation results. (b) Box plot of Spearman correlation coefficients (SCC) of the 5-fold cross-validation results.*

***Fig. R5. The cross-chromosome predictive performance of pretrained EpiGePT and EpiGePTseq after fine-tuning on mouse data.*** *(a) The bar plot illustrates the Pearson correlation coefficient (PCC), Spearman correlation coefficient (SCC), mean squared error (MSE), and R square between the predicted and true chromatin openness for EpiGePT and EpiGePT-seq, across 22,750 bins in the brain, 22,450 bins in the lung, and 16,200 bins in the kidney. (b) The scatter plots illustrate the relationship between the predicted and true chromatin openness for EpiGePT and EpiGePT-seq, across 22,750 bins in the brain, 22,450 bins in the lung, and 16,200 bins in the kidney.*

***Fig. R10. The Pearson correlation coefficient (PCC) for predicting chromatin accessibility (DNaseseq) under different numbers of training cell lines/tissues.*** *(a) Line plot showing the average PCC across 26 test cell types, as a function of the number of training cell types. (b) Box plot showing the distribution of PCC across 26 test cell types for different numbers of training cell types. (c) Bar plot with error bars showing the PCC across 26 test cell types for different numbers of training cell types.*

## MAJOR SPECIFIC COMMENT 2

**2) As mentioned in Suppl. Text S5, the authors modified Enformer by: 1) reducing the number of layers to 3, 2) Enformer+: using reference TF binding profiles. Can the authors present a performance comparison between EpiGePT, Enformer original (i.e., pre-trained Enformer model from Avsec et al. 2021), Enformer with 3 encoder layers but without using reference TF binding profiles, and Enformer+ which uses 3 encoder layers and reference TF binding profiles. I understand that original Enformer model does not satisfy cross-validation principle but this benchmarking would provide a upper bound of the performance metrics. Again, the authors need to choose one version of EpiGePT model (I would prefer EpiGePT-3D using reference TF information and chromatin interactions).**

## *RESPONSE TO MAJOR SPECIFIC COMMENT 2*

*Thank you for this constructive comment. We agree with the reviewer that it is important to include a performance comparison between EpiGePT, Enformer original (pretrained Enformer), Enformer+, and retrained Enformer. To clarify, the manuscript already includes comparisons between EpiGePT and both the original pretrained Enformer and the retrained Enformer models. We apologize for not clearly highlighting these comparisons in the manuscript. Next, we will provide a detailed explanation of these comparisons in two parts (retrained Enformer and pretrained Enformer) for the reviewer, and part of the experimental results related to this comment has already been addressed in our response to major comment 1.*

*First, for the comparison with the retrained Enformer (Enformer with 3 encoder layers but without using reference TF binding profiles, and Enformer+ which uses 3 encoder layers and reference TF binding profiles), we have conducted a five-fold cross-validation on the chromatin accessibility (DNase-seq profiles) cross-cell-type prediction task. As shown in* ***Fig. R18****, EpiGePT achieved superior results, with a median PCC of 0.787, outperforming both Enformer+ (0.779) and the retrained Enformer (0.735). The purpose of this comparison is to demonstrate that model architecture of EpiGePT offers stronger predictive accuracy than Enformer when trained on the same dataset. Specifically,* ***1)*** *the comparison between EpiGePT and the retrained Enformer shows that using Enformer’s pure DNA sequence architecture makes it difficult to accurately predict epigenomic signals in unseen cell types. This is because the DNA sequences are identical across different cell types, so even when trained in various cellular contexts, the retrained Enformer cannot distinguish the same region under different contexts and can only predict an averaged epigenomic signal. This comparison highlights the improvement brought by EpiGePT’s use of reference TF profiles.* ***2)*** *the comparison between EpiGePT and the retrained Enformer+ shows that the convolution tower and other operations applied to the DNA sequence in Enformer did not improve predictive accuracy. In fact, when incorporating reference TF binding profiles, the main difference between Enformer+ and EpiGePT lies in the CNN module used for DNA feature extraction. Our experimental results indicate that the more complex operations, such as the convolution tower in Enformer, did not enhance performance on our dataset. Instead, there was a slight decline in accuracy. Moreover, these operations significantly increase the demand for computational and memory resources.* ***3)*** *By comparing the retrained Enformer+ with the retrained Enformer, we can also confirm the improvement in predictive accuracy brought by the incorporation of reference TF binding profiles. The main difference between Enformer+ and Enformer is that the former has crosscell type prediction capabilities, which is the primary reason we designed this model. In summary, from the above experiments comparing with the retrained Enformer, we can conclude that under the same training and testing data, EpiGePT demonstrates superior predictive performance compared to the Enformer architecture.*

*In addition, we provide an explanation for using the retrained Enformer with 3 encoder layers. Although the original Enformer model was trained with 11 encoder layers, during the process of adapting the Enformer architecture to our processed data, we found that the number of encoder layers did not have a decisive impact on the model’s performance. We tested the Enformer with 3, 6, 9, and 12 encoder layers for DNase-seq prediction, and observed that there were no significant changes in the average PCC and SCC. In fact, the 3-layer model showed slightly better performance in terms of median scores, although the highest improvement in PCC was only about 0.7% (****Fig. R19****). Specifically, the average PCC for the 3-layer model was*

*0.724, slightly lower than the 9-layer model at 0.726 but higher than the 12-layer model at 0.718. Therefore, since the number of layers did not substantially affect the model’s performance, we opted to use the 3-layer model to reduce computational resource requirements during retraining.*

*Second, for the comparison with the original Enformer (pretrained Enformer), since the original Enformer was trained on 5,313 human genomic tracks based on the hg38 genome and covered a large number of genomic regions, we used a masking strategy for training on hg38 data to include more cell types. In Fig. 2d of the original manuscript, we already compared EpiGePT with the original Enformer. Because the original Enformer cannot predict unseen genomic tracks, we selected 78 genomic tracks from its 5,313-dimensional output for genome-wide prediction. The cell types associated with these tracks were all unseen during EpiGePT training, although some regions might overlap with the Enformer training set. Even under this setting favarable to Enformer, EpiGePT still demonstrated an improved prediction performance over Enformer.*

*Morever, based on this comment, we conducted an extra experiment to compare the performance of EpiGePT, EpiGePT-3D only using 11 cell types with 3D genome data for training, and the original Enformer. The results showed that, even with a significantly reduced number of cellular contexts, EpiGePT-3D still performed slightly better than the original Enformer on the same 78 genomic tracks, and EpiGePT-3D outperformed EpiGePT (****Fig. R20****) slightly. This suggests that in the future, when sufficient 3D genome data becomes more available, EpiGePT-3D will be the better choice. However, at the current stage, due to limitations in the number of training cellular contexts, we still choose EpiGePT as the base model for researchers to use.*

*In summary, since the input and output settings of the original Enformer and retrained Enformer differ, we compared EpiGePT with the retrained Enformer and the original Enformer separately. These comparisons collectively demonstrate that EpiGePT has the capability for cross-cell-type prediction of epigenomic signals due to its model design, and achieves higher predictive accuracy in practice. We also recommend that researchers use the pretrained EpiGePT model for future applications. Additionally, we have made the EpiGePT-3D model available on our website (http://health.tsinghua.edu.cn/epigept) and will update it when more 3D genome data and paired epigenomic data become available.*

***Fig. R19. The performance comparison of retrained Enformer with different numbers of encoder layers for predicting chromatin accessibility.*** *(a) The boxplot of PCC for predicting chromatin accessibility across 26 test cellular contexts by Enformer with varying numbers of encoder layers (3, 6, 9, and 12). (b) The boxplot of SCC for predicting chromatin accessibility across 26 test cellular contexts by Enformer with varying numbers of encoder layers (3, 6, 9, and 12).*

***Fig. R20. The performance comparison of original Enformer with EpiGePT and EpiGePT-3D on 78 genomic tracks.*** *(a) The boxplot of PCC for the prediction of 78 genomic tracks across 15,830,000 non-overlapping 128bp bins by the original Enformer, EpiGePT, and EpiGePT-3D (trained on 11 cellular contexts). (b) The boxplot of SCC for the prediction of 78 genomic tracks across 15,830,000 non-overlapping 128bp bins by the original Enformer, EpiGePT, and EpiGePT-3D (trained on 11 cellular contexts).*

**MINOR SPECIFIC COMMENT 1**

**1) Fig. S3-e: Authors should indicate different track types for individual correlation plots.**

## *RESPONSE TO MINOR SPECIFIC COMMENT 1*

*We appreciate the reviewer for this meticulous comment. In fact, we presented the predicted results for chromatin accessibility (DNase-seq profile) in Fig. S3e, and we indeed did not clearly indicate this in the figure. In the revised supplementary materials, we have now explicitly labeled the track type in the Fig. S3e and provided a clearer explanation in the figure caption, which enhances both the clarity and readability of the manuscript.*

## MINOR SPECIFIC COMMENT 2

**2) When referring supplementary figures, such as Fig. S4, please refer to the exact figure i.e., Fig. S4(b).**

## *RESPONSE TO MINOR SPECIFIC COMMENT 2*

*Thank you for this detailed comment. In the revised manuscript, we have conducted a thorough review and made the necessary revisions to ensure that all references to supplementary figures point to the exact subfigures. We sincerely thank the reviewer for this comment, which has contributed to enhancing the readability of the manuscript.*

## MINOR SPECIFIC COMMENT 3

**3) Fig. 3h - EpiGePT should be EpiGePT(finetuned). The performance values and scales are not clear. Performance should be compared with EpiGePT-3D model.**

## *RESPONSE TO MINOR SPECIFIC COMMENT 3*

*Thank you for this constructive comment. The reviewer raised three points in this comment.*

*Below, we address and explain each of these issues in detail.*

***First****, regarding the issue with the figure captions, we have updated the revised manuscript and supplementary materials to clearly indicate that the performance shown is for the finetuned EpiGePT. This comment has contributed to enhancing the accuracy and clarity of the figure captions.*

***Second****, regarding the issue of unclear values and scales that may make it difficult for readers to interpret the scales and values directly. To address this, we have revised Fig. 3h with clear references for both values and scales. Additionally, to enhance clarity, we have also provided the quantitative results of the five-fold cross-validation performance in tables, which has been included in table S15-S18 in the revised supplementary materials (****Table R5-R8****).*

***Third****, concerning the comparison between the performance of EpiGePT and the EpiGePT-3D model: The HiChIP data used in this study were obtained from the HiChIPdb database (Zeng, et al., 2023), where all datasets are aligned to the GRCh37 (hg19) reference genome. Consequently, Fig. 3h illustrates the performance of the finetuned EpiGePT trained on the hg19 reference genome. In contrast, EpiGePT-3D was pretrained on the hg38 genome with a larger and more diverse set of cellular contexts. Due to these differences in reference genomes, a direct comparison between pretrained EpiGePT and EpiGePT-3D on this dataset is not feasible. However, following the reviewer’s comment, we conducted additional experiments to facilitate a direct comparison between EpiGePT-3D and the baseline methods.*

*To evaluate the ability of EpiGePT-3D and baseline methods in predicting HiChIP loops after fine-tuning, we converted the HiChIP loop data from hg19 to the hg38 reference genome using liftover (Raney, et al., 2024). Subsequently, we retrained baseline models including the k-merbased MLP and DeepTACT on the hg38 reference. Notably, the chromatin accessibility data required for DeepTACT were also re-annotated on the hg38 genome using the OpenAnnotate (Chen, et al., 2021; Gao, et al., 2024). As shown in* ***Fig. R21*** *and* ***Fig. R22****,* ***EpiGePT-3D consistently outperformed baseline methods across different cell lines and distance ranges after fine-tuning****. For instance, in the 0-20 kbp range of K562 cells, EpiGePT-3D achieved an average auROC that was 8.5% higher than DeepTACT, the best-performing baseline method. It is worth noting that the overall performance of all three methods declined slightly after the liftover, which we attribute to limitations in the liftover process. Specifically, when anchoring on the original interaction center, some regions could not be successfully lifted over (e.g., due to deletions), thereby reducing the reliability of the ground truth and impacting model performance even after fine-tuning and retraining.*

*Regarding the comparison between EpiGePT-3D and EpiGePT, this has already been validated in the original manuscript (****Fig. 2d-e****). The results demonstrate that EpiGePT-3D achieves an improvement by directly leveraging self-attention for selecting enhancer-promoter (E-P) pairs. We evaluated EpiGePT’s performance for predicting chromatin interactions using three different settings: EpiGePT-3D (setting B), where chromatin interaction data was integrated into the model as constraints during training, and EpiGePT finetuned, where chromatin interaction data was used as labels for fine-tuning the model. These represent two distinct applications of the model. To investigate the contribution of incorporating the 3D genome in EpiGePT-3D, the most straightforward and fair approach is to utilize self-attention scores before and after incorporation to filter E-P pairs. This comparison has already been conducted in the original manuscript (****Fig. 3d****). In addition, following the reviewer’s comment, we compared finetuned EpiGePT and EpiGePT-3D by concatenating the token-level features obtained from the model at the anchors of the corresponding chromatin interactions and training a MLP as the prediction layer. As shown in* ***Fig. R21-R22****, although this approach may not directly compare the biological relevance of the self-attention scores between the two models, EpiGePT-3D still demonstrated a slight improvement in the mean performance in most crossvalidation settings. This further supports the notion that incorporating 3D genome data can enhance the model’s interpretability, especially in elucidating gene regulatory relationships.*

*In conclusion, based on the reviewer’s suggestions, we have revised Fig. 3h to make the scales and values clearer, and the specific performance metrics have been organized into tables included in the revised supplementary materials. More importantly, we have conducted further experiments to compare the finetuned performance of EpiGePT-3D with baseline methods (****Fig. R21-22****), and we clarified the details regarding the comparison between EpiGePT-3D and EpiGePT. We thank the reviewer for this detailed and constructive comment again, which has enhanced the comprehensiveness of the results and the quality of the manuscript.*

***Table R5.*** *Comparison of the performance between fine-tuned EpiGePT and baseline methods in predicting HiChIP loops on the K562 cell line (positive-to-negative sample ratio of 1:1).*

*Metric	Model	0-20kbp	20-40kbp	40-64kbp*

| *Accuracy* | *EpiGePT*  *(finetuned)* | ***0.950 ± 0.013*** | ***0.959 ± 0.007*** | ***0.979 ± 0.008*** |
| --- | --- | --- | --- | --- |
|  | *Kmer-MLP* | *0.709 ± 0.016* | *0.707 ± 0.010* | *0.735 ± 0.015* |
|  | *DeepTACT* | *0.751 ± 0.042* | *0.795 ± 0.058* | *0.829 ± 0.015* |
| *auROC* | *EpiGePT*  *(finetuned)* | ***0.990 ± 0.002*** | ***0.990 ± 0.003*** | ***0.998 ± 0.001*** |
|  | *Kmer-MLP* | *0.786 ± 0.020* | *0.783 ± 0.017* | *0.814 ± 0.011* |
|  | *DeepTACT* | *0.866 ± 0.020* | *0.889 ± 0.030* | *0.908 ± 0.008* |
| *auPR* | *EpiGePT*  *(finetuned)* | ***0.988 ± 0.003*** | ***0.988 ± 0.005*** | ***0.997 ± 0.001*** |
|  | *Kmer-MLP* | *0.784 ± 0.030* | *0.774 ± 0.028* | *0.805 ± 0.018* |
|  | *DeepTACT* | *0.876 ± 0.014* | *0.888 ± 0.034* | *0.907 ± 0.012* |

***Table R6.*** *Comparison of the performance between fine-tuned EpiGePT and baseline methods in predicting HiChIP loops on the GM12878 cell line (positive-to-negative sample ratio of 1:1).*

*Metric	Model	0-20kbp	20-40kbp	40-64kbp*

| *Accuracy* | *EpiGePT*  *(finetuned)* | ***0.937 ± 0.002*** | ***0.937 ± 0.005*** | ***0.928 ± 0.022*** |
| --- | --- | --- | --- | --- |
|  | *Kmer-MLP* | *0.631 ± 0.025* | *0.683 ± 0.024* | *0.685 ± 0.011* |
|  | *DeepTACT* | *0.798 ± 0.067* | *0.905 ± 0.033* | *0.903 ± 0.009* |
| *auROC* | *EpiGePT*  *(finetuned)* | ***0.982 ± 0.004*** | ***0.984 ± 0.003*** | ***0.985 ± 0.003*** |
|  | *Kmer-MLP* | *0.694 ± 0.028* | *0.750 ± 0.028* | *0.758 ± 0.005* |
|  | *DeepTACT* | *0.886 ± 0.056* | *0.962 ± 0.020* | *0.960 ± 0.010* |
| *auPR* | *EpiGePT*  *(finetuned)* | ***0.980 ± 0.004*** | ***0.982 ± 0.004*** | ***0.982 ± 0.004*** |
|  | *Kmer-MLP* | *0.689 ± 0.048* | *0.747 ± 0.016* | *0.764 ± 0.007* |
|  | *DeepTACT* | *0.885 ± 0.057* | *0.959 ± 0.016* | *0.952 ± 0.011* |

***Table R7.*** *Comparison of the performance between fine-tuned EpiGePT and baseline methods in predicting HiChIP loops on the K562 cell line (positive-to-negative sample ratio of 1:2).*

*Metric	Model	0-20kbp	20-40kbp	40-64kbp*

| *Accuracy* | *EpiGePT*  *(finetuned)* | ***0.944 ± 0.015*** | ***0.961 ± 0.005*** | ***0.969 ± 0.003*** |
| --- | --- | --- | --- | --- |
|  | *Kmer-MLP* | *0.764 ± 0.014* | *0.779 ± 0.015* | *0.790 ± 0.010* |
|  | *DeepTACT* | *0.807 ± 0.021* | *0.853 ± 0.007* | *0.876 ± 0.014* |
| *auROC* | *EpiGePT*  *(finetuned)* | ***0.992 ± 0.002*** | ***0.993 ± 0.001*** | ***0.995 ± 0.002*** |
|  | *Kmer-MLP* | *0.815 ± 0.014* | *0.833 ± 0.013* | *0.851 ± 0.008* |
|  | *DeepTACT* | *0.885 ± 0.011* | *0.919 ± 0.003* | *0.934 ± 0.006* |
| *auPR* | *EpiGePT*  *(finetuned)* | ***0.982 ± 0.004*** | ***0.984 ± 0.002*** | ***0.988 ± 0.004*** |
|  | *Kmer-MLP* | *0.720 ± 0.021* | *0.730 ± 0.024* | *0.757 ± 0.015* |
|  | *DeepTACT* | *0.828 ± 0.020* | *0.869 ± 0.004* | *0.894 ± 0.007* |

***Table R8.*** *Comparison of the performance between fine-tuned EpiGePT and baseline methods in predicting HiChIP loops on the GM12878 cell line (positive-to-negative sample ratio of 1:2).*

*Metric	Model	0-20kbp	20-40kbp	40-64kbp*

| *Accuracy* | *EpiGePT*  *(finetuned)* | ***0.922 ± 0.028*** | ***0.940 ± 0.008*** | ***0.918 ± 0.026*** |
| --- | --- | --- | --- | --- |
|  | *Kmer-MLP* | *0.712 ± 0.010* | *0.727 ± 0.007* | *0.726 ± 0.011* |
|  | *DeepTACT* | *0.899 ± 0.020* | *0.921 ± 0.032* | *0.914 ± 0.023* |
| *auROC* | *EpiGePT*  *(finetuned)* | ***0.984 ± 0.001*** | ***0.983 ± 0.003*** | ***0.984 ± 0.003*** |
|  | *Kmer-MLP* | *0.735 ± 0.013* | *0.765 ± 0.007* | *0.772 ± 0.020* |
|  | *DeepTACT* | *0.956 ± 0.015* | *0.972 ± 0.018* | *0.974 ± 0.005* |
| *auPR* | *EpiGePT*  *(finetuned)* | ***0.968 ± 0.002*** | ***0.958 ± 0.007*** | ***0.963 ± 0.007*** |
|  | *Kmer-MLP* | *0.628 ± 0.019* | *0.641 ± 0.012* | *0.644 ± 0.025* |
|  | *DeepTACT* | *0.917 ± 0.023* | *0.943 ± 0.031* | *0.947 ± 0.005* |

***Fig. R21. Comparison of the performance between fine-tuned EpiGePT-3D, EpiGePT and baseline methods in predicting HiChIP loops****. (a) ROC curves (5-fold cross validation) comparing the performance of EpiGePT-3D, EpiGePT and baseline methods in predicting chromatin interactions (HiChIP loops) on the K562 cell line (positive-to-negative sample ratio of 1:1). (b) ROC curves (5-fold cross validation) comparing the performance of EpiGePT-3D, EpiGePT and baseline methods in predicting chromatin interactions (HiChIP loops) on the*

*GM12878 cell line (positive-to-negative sample ratio of 1:1).*

***Fig. R22. Comparison of the performance between fine-tuned EpiGePT-3D, EpiGePT and baseline methods in predicting HiChIP loops****. (a) PR curves (5-fold cross validation) comparing the performance of EpiGePT-3D and baseline methods in predicting chromatin interactions (HiChIP loops) on the K562 cell line (positive-to-negative sample ratio of 1:1). (b) PR curves (5-fold cross validation) comparing the performance of EpiGePT-3D, EpiGePT and baseline methods in predicting chromatin interactions (HiChIP loops) on the GM12878 cell line (positive-to-negative sample ratio of 1:1).*

***Reference***

*Alvarez-Jarreta, J., et al. (2024) VEuPathDB: the eukaryotic pathogen, vector and host bioinformatics resource center in 2023. Nucleic acids research, 52(D1), D808-D816. Avsec, Ž., et al. (2021) Effective gene expression prediction from sequence by integrating longrange interactions. Nature methods, 18(10), 1196-1203.*

*Barrett, T., et al. (2012) NCBI GEO: archive for functional genomics data sets—update.*

*Nucleic acids research, 41(D1), D991-D995.*

*Bernstein, B.E., et al. (2010) The NIH roadmap epigenomics mapping consortium. Nature biotechnology, 28(10), 1045-1048.*

*Biology, C.S.-C., et al. (2023) CZ CELLxGENE Discover: A single-cell data platform for scalable exploration, analysis and modeling of aggregated data. BioRxiv, 2023.2010.*

*2030.563174.*

*Breschi, A., Gingeras, T.R. and Guigó, R. (2017) Comparative transcriptomics in human and mouse. Nature Reviews Genetics, 18(7), 425-440.*

*Chen, S., et al. (2021) OpenAnnotate: a web server to annotate the chromatin accessibility of genomic regions. Nucleic Acids Research, 49(W1), W483-W490.*

*Consortium, E.P. (2012) An integrated encyclopedia of DNA elements in the human genome.*

*Nature, 489(7414), 57. d Galbraith, E., sc Merleau, N. and mcdonald Smith, B. (2023) The human cell count and size distribution. Proceedings of the National Academy of Sciences, 120(39), e2303077120. Ernst, J. and Kellis, M. (2017) Chromatin-state discovery and genome annotation with ChromHMM. Nature protocols, 12(12), 2478-2492.*

*Gao, Z., Jiang, R. and Chen, S. (2024) OpenAnnotateApi: Python and R packages to efficiently annotate and analyze chromatin accessibility of genomic regions. Bioinformatics Advances, 4(1), vbae055.*

*Heinz, S., et al. (2010) Simple combinations of lineage-determining transcription factors prime cis-regulatory elements required for macrophage and B cell identities. Molecular cell, 38(4), 576-589.*

*Kulakovskiy, I.V., et al. (2018) HOCOMOCO: towards a complete collection of transcription factor binding models for human and mouse via large-scale ChIP-Seq analysis. Nucleic acids research, 46(D1), D252-D259.*

*Liu, Q., et al. (2022) DeepCAGE: incorporating transcription factors in genome-wide prediction of chromatin accessibility. Genomics, Proteomics & Bioinformatics, 20(3), 496-507. Lizio, M., et al. (2015) Gateways to the FANTOM5 promoter level mammalian expression atlas. Genome biology, 16, 1-14.*

*Nair, S., et al. (2019) Integrating regulatory DNA sequence and gene expression to predict genome-wide chromatin accessibility across cellular contexts. Bioinformatics, 35(14), i108i116.*

*Raney, B.J., et al. (2024) The UCSC genome browser database: 2024 update. Nucleic Acids Research, 52(D1), D1082-D1088.*

*Yates, A.D., et al. (2022) Ensembl Genomes 2022: an expanding genome resource for nonvertebrates. Nucleic acids research, 50(D1), D996-D1003.*

*Zeng, W., et al. (2023) HiChIPdb: a comprehensive database of HiChIP regulatory interactions.*

*Nucleic acids research, 51(D1), D159-D166.*

*Zhou, W., et al. (2017) Genome-wide prediction of DNase I hypersensitivity using gene expression. Nature communications, 8(1), 1-17.*

**Second round of review**

**Reviewer 1**

I congratulate the authors to an incredibly detailed re-analysis of their dataset and for clarifying all my issues. My concerns have been fully addressed and I can now recommend an editorial decision to publish this work.

**Reviewer 2**

The authors have adequately addressed my comments. I therefore recommend accepting the manuscript.
